# Special Issue: Newborn Health in Uganda

**DOI:** 10.3402/gha.v8.27574

**Published:** 2015-03-31

**Authors:** Kate Kerber, Stefan Peterson, Peter Waiswa

**Affiliations:** 1 Save the Children, Cape Town, South Africa; 2 Makerere University, Kampala, Uganda; 3 Karolinska Institutet, Stockholm, Sweden; 4 Uppsala University, Uppsala, Sweden; 5 Iganga/Mayuge Health Demographic Surveillance Site, Kampala, Uganda

Over the past decade, birth and the first month of life has gained visibility as a critical time to intervene to continue momentum for child survival given that almost half of child deaths are now in the neonatal period (0–28 days after birth) (1). Investment in a healthy birth gives a triple return as this is the moment of greatest risk for women, stillbirths, and newborns and also crucial for child development and human capital (1). Yet despite great potential for mortality reduction, progress remains slow, with neonatal mortality reducing at about half the speed of maternal mortality or child mortality after the first month (1). The slowest progress has been in sub-Saharan Africa: at current rates of change it will be over a century before an African newborn has the same chance of survival as a baby born in Europe or North America (1). This gap reflects ongoing low visibility in comparison to the massive burden to families, to the health system, and to future development potential. Furthermore, this gap reflects the pervasive myth that newborn deaths are inevitable, a lack of visible successes for programmes at scale and a lack of leadership (2, 3).

The results of the Uganda Newborn Study (UNEST) as described by Waiswa et al. (4) and the other papers in this supplement are important for Uganda with implications for the rest of the continent, encapsulating many of the evidence shifts from the last decade, and giving clear messages to accelerate progress (4). UNEST was influenced by the 2005 *Lancet* neonatal survival series which identified highly cost-effective packages of interventions that could avert more than two-thirds of all neonatal deaths (5). At that time the majority of births in Africa and Asia were at home and an important message was that an estimated one third of neonatal deaths, especially in high mortality settings, could be prevented at community level (5). All of the well-known community-based trials at the time were from South Asia, and most were smaller scale efficacy trials, highlighting the need for contextual adaptation and a focus on effectiveness. In response to the 2005 *Lancet* neonatal series, Uganda held the first national stakeholder meeting on newborn survival, and undertook a situation analysis. A priority gap identified was the lack of locally relevant evidence regarding use of existing community and facility systems to address the main causes of neonatal deaths. With funding from The Bill & Melinda Gates Foundation through Save the Children's Saving Newborn Lives programme, researchers in Uganda partnered with national policymakers and district leaders to conduct UNEST as a two-arm cluster randomised trial evaluating a package of home visits during pregnancy and the postnatal period, with improved facility-based care effecting both the intervention and control arms (4).

The content of the intervention package was adapted from South Asian evidence, linking with concurrent adaptations in the Africa Newborn Network of trials in five other countries (6). The main cadre engaged in UNEST was a community health worker (CHW), locally called a Village Health Team (VHT) member. This worker was recruited by their own community in line with national policy (developed alongside UNEST as described by Waiswa et al. (7)) and trained to identify pregnant women and make five home visits: two before and three in the first week after birth. The package was entirely preventive, with no home delivery care or home-based treatment of infections. Facility quality improvement included minimal upgrades to basic equipment, training in obstetric and newborn care, and strengthening management procurement, monitoring and supervision processes as described by Namazzi et al. (8). Linkages were made between community and facility care including targeted messages for home-care and referrals (9).

UNEST took place in rural eastern Uganda within the Iganga-Mayugye health and demographic surveillance site, a member of the INDEPTH Network of 52 field sites in Africa, Asia and Oceania (10). Data collection was based both on household surveys, and the health and demographic surveillance sites. Whilst births and deaths were tracked, UNEST was not powered to detect mortality change and was focused on coverage change. So what can we learn from UNEST?

**1. Community care is pro-poor in this rural African context, but scalability depends on recognition of community care as a part of the health system with consistent funding and supervision**: The UNEST results demonstrate that home visits were possible to achieve with relatively high coverage (over 40% on the first day after birth and almost two-thirds visited in the first week), and that home behaviours could be changed by this interaction. Immediate and exclusive breastfeeding were significantly higher in the intervention arm compared to control (72.6% vs. 66.0%; *p*<0.016 and 81.8% vs. 75.9%; *p*<0.042, respectively). Skin-to-skin care after birth and cord cutting with a clean instrument were marginally higher (80.7% vs. 72.2%; *p*<0.071 and 88.1% vs. 84.4%; *p*<0.023, respectively). Half (49.6%) the mothers in the intervention arm delayed bathing their baby by more than 24 hours, compared to 35.5% in the control arm (p<0.001). Dry umbilical cord care was significantly higher in intervention areas (63.9% vs. 53.1%; p<0.001). However, whilst skilled attendance at delivery increased by 21% in the intervention arm, it also increased in the control arm (by 19%) and there was no difference in care-seeking for newborn illness, which was high (around 95%) in both arms (4). This underlines the importance of a randomised, control arm to detect changes related to the intervention, especially in a rapidly changing health system context (11).

Importantly, in contrast to the marked inequity around facility-based care at birth and for illness, these home visits were pro-poor, with more women in the poorest quintile, who are most at risk, visited by a CHW compared to families in the least poor quintile (4).

Whilst these findings are encouraging, a key question remains regarding scalability. This trial, while implemented mainly within the existing system and designed together with national and district-level stakeholders (7), was still a trial, and relatively small-scale. Encouraging indicators of sustainability was the 100% retention of the CHWs and acceptability by families and facility-based health workers (12). A multi-country economic analysis is in process using the Cost of Integrated Newborn Care (COINCare) tool, designed by the South African Medical Research Council in collaboration with Save the Children's Saving Newborn Lives which will present the detailed cost of this package when adapted for scale-up at high coverage.

However, even with relatively low cost and high CHW retention, expanding this package may be challenging given the insecurity of district budget support for the VHTs, which in many cases relies on donor support. Current donor investments in scale-up of VHTs for Integrated Community Case Management of childhood illness provides an opportunity for incorporating scale-up of maternal and newborn home visits, something government and partners are keen to test (13). In addition, the careful attention to supervisory systems and linking communities to facilities was in many cases led by the UNEST team and would be critical to institutionalise and sustain in the routine system.

**2. Quality facility-based care is crucial for impact:** Whilst facility birth coverage increased and care-seeking for sick newborns was remarkably high, the process data reported by Namazzi et al. underlines that even with quality improvement of facility care for birth and for small and sick newborns, there were many systemic challenges with staff shortages and attrition, supply chain failures for drugs and equipment despite management and logistics support (9). This quality gap in both public and private health facilities (14) is a major barrier to saving lives for women and newborns since the highest impact care is at facility level (15), and the reality of the quality gap has been shown in other African Newborn Network studies in Ghana (16) and elsewhere. Addressing the quality and equity gaps for care at birth and for small and sick newborns is the top priority of the *Every Newborn Action Plan* (17).

Improving the quality of facility-based care around the time of birth is especially critical to reducing maternal deaths and stillbirths, but this should be done with concurrent interventions to address demand-side barriers. In UNEST, Kiguli et al. (18) and Nalwadda et al. (9) sought to understand the sociocultural context around the time of birth and the reasons for not seeking care promptly, in order to design and implement interventions effectively.

**3. Innovations can address key challenges:** Other locally driven innovations were delivered by UNEST. The study piloted a social autopsy module for stillbirths and newborn deaths, assigning each death to various delays across the health system (19). A need for better identification and follow-up of small newborns led to the development and use of a foot length card for use in homes during VHT visits (20) in partnership with the African Newborn Network site in Tanzania (21).

While policies and attention have tended to focus on the public sector, this series of papers also reports on differences observed between the public and private facilities, with the knowledge that a large proportion of families are delivering babies and seeking treatment in the private sector (14). The proportion of births taking place in private facilities reduced over the course of the trial and private facilities did not show convincingly better services, underlining the need for context-specific data rather than blanket statements on private and public sector comparisons.

With more data coming from a variety of settings that have tested and rolled out community-based home visit packages, lessons are emerging on innovative methods of helping families prepare for a safe and clean birth (22), as well as different ways to ensure families receive the multiple interactions needed in order to extract maximum benefit from these integrated services (23).

**4. Local leadership is key and requires intentional strategies:** A key challenge to progress for newborn survival, especially in Africa, has been lack of leaders, and lack of designated programme managers (2). UNEST provides a model of local capacity-building through high quality research informing national policy (7) and higher education: during the course of the study 3 PhDs were completed with another 3 ongoing and 16 MSc and MPH degrees were awarded to individuals working on different UNEST components. Individuals from the Iganga-Mayugye surveillance site have provided leadership to a Maternal and Newborn Working Group within the INDEPTH Network to strengthen data and research capacity across sites. A much more intentional approach to leadership development for newborn care and for RMNCH is needed, building African Centres of Excellence (17).

**Figure:**
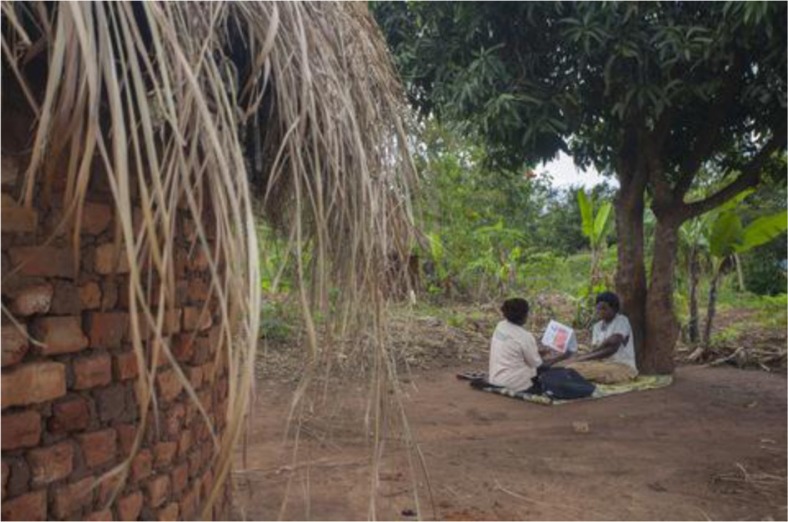
Ian Hurley/Save the Children, Uganda.

Newborns around the world today face a more certain future than they did 10 years ago, but for over 15,000 families, the loss of a baby due to stillbirth or neonatal death remains a daily reality. The *Every Newborn Action Plan*, ratified at the World Health Assembly in 2014 (24) is focusing attention on unfinished business of the Millennium Development Goals and on the crucial time of birth in the lifecycle as being key to the post-2015 development agenda. Many countries, including Uganda, have committed at the highest level to doing more for women and babies. Uganda has gone further than many countries with a national newborn steering committee, standard for care at all levels and an increasing voice from parliamentarians, but there remains a policy-practice gap (25). Let us learn from this evidence, and add more as we move faster for our smallest and most vulnerable citizens.
