# Supplementary material for: Special Issue: Newborn Health in Uganda
Source: Glob Health Action. 2015 Mar 31;8:10.3402/gha.v8.27574. doi: 10.3402/gha.v8.27574 (PMC4385224; doi:10.3402/gha.v8.27574)
Supplement: Related texts

# Sub article: Effect of the Uganda Newborn Study on care-seeking and care practices: a cluster-randomised controlled trial

**DOI:** 10.3402/gha.v8.24584

**Published:** 2015-03-31

**Authors:** Peter Waiswa, George Pariyo, Karin Kallander, Joseph Akuze, Gertrude Namazzi, Elizabeth Ekirapa-Kiracho, Kate Kerber, Hanifah Sengendo, Patrick Aliganyira, Joy E. Lawn, Stefan Peterson, on behalf of the Uganda Newborn Study Team

**Affiliations:** 1 Department of Health Policy, Planning and Management, School of Public Health, College of Health Science, Makerere University, Kampala, Uganda; 2 Global Health, Department of Public Health Sciences, Karolinska Institutet, Stockholm, Sweden; 3 Iganga-Mayuge Health and Demographic Surveillance Site, Iganga-Mayuge, Uganda; 4 Malaria Consortium, London, UK; 5 Save the Children, Cape Town, South Africa; 6 MARCH Unit, London School of Hygiene and Tropical Medicine, London, UK; 7 International Maternal and Child Health Unit, Department of Women's and Children's Health, Uppsala University, Uppsala, Sweden

**Keywords:** community health workers, health system strengthening, kangaroo mother care, maternal care, newborn care, neonatal mortality, randomised controlled trial, Uganda

## Abstract

**Background:**

Care for women and babies before, during, and after the time of birth is a sensitive measure of the functionality of any health system. Engaging communities in preventing newborn deaths is a promising strategy to achieve further progress in child survival in sub-Saharan Africa.

**Objective:**

To assess the effect of a home visit strategy combined with health facility strengthening on uptake of newborn care-seeking, practices and services, and to link the results to national policy and scale-up in Uganda.

**Design:**

The Uganda Newborn Study (UNEST) was a two-arm cluster-randomised controlled trial in rural eastern Uganda. In intervention villages volunteer community health workers (CHWs) were trained to identify pregnant women and make five home visits (two during pregnancy and three in the first week after birth) to offer preventive and promotive care and counselling, with extra visits for sick and small newborns to assess and refer. Health facility strengthening was done in all facilities to improve quality of care. Primary outcomes were coverage of key essential newborn care behaviours (breastfeeding, thermal care, and cord care). Analyses were by intention to treat. This study is registered as a clinical trial, number ISRCTN50321130.

**Results:**

The intervention significantly improved essential newborn care practices, although many interventions saw major increases in both arms over the study period. Immediate breastfeeding after birth and exclusive breastfeeding were significantly higher in the intervention arm compared to the control arm (72.6% vs. 66.0%; *p*=0.016 and 81.8% vs. 75.9%, *p*=0.042, respectively). Skin-to-skin care immediately after birth and cord cutting with a clean instrument were marginally higher in the intervention arm versus the control arm (80.7% vs. 72.2%; *p*=0.071 and 88.1% vs. 84.4%; *p*=0.023, respectively). Half (49.6%) of the mothers in the intervention arm waited more than 24 hours to bathe the baby, compared to 35.5% in the control arm (*p*<0.001). Dry umbilical cord care was also significantly higher in intervention areas (63.9% vs. 53.1%, *p*<0.001). There was no difference in care-seeking for newborn illness, which was high (around 95%) in both arms. Skilled attendance at delivery increased in both the intervention (by 21%) and control arms (by 19%) between baseline and endline, but there was no significant difference in coverage across arms at endline (79.6% vs. 78.9%; *p*=0.717). Home visits were pro-poor, with more women in the poorest quintile visited by a CHW compared to families in the least poor quintile, and more women who delivered at home visited by a CHW after birth (73.6%) compared to those who delivered in a hospital or health facility (59.7%) (*p*<0.001). CHWs visited 62.8% of women and newborns in the first week after birth, with 40.2% receiving a visit on the critical first day of life.

**Conclusion:**

Consistent with results from other community newborn care studies, volunteer CHWs can be effective in changing long-standing practices around newborn care. The home visit strategy may provide greater benefit to poorer families. However, CHW strategies require strong linkages with and concurrent improvement of quality through health system strengthening, especially in settings with high and increasing demand for facility-based services.

Newborn survival has recently emerged as a global and national public health issue, driven in part by the fourth Millennium Development Goal (MDG) for child survival. Under-five mortalities are estimated to have reduced by 47% globally between 1990 and 2012, while deaths in the first month of life (the neonatal period) declined by 37% (1). Each year in Uganda there are an estimated 35,000 newborn deaths (1), with an additional 40,000 babies stillborn (2). The proportion of under-five deaths that occur in the neonatal period has increased, from 22% in 1990 to 33% in 2012 (1). With a growing proportion of under-five deaths occurring amongst newborns, child survival will increasingly be determined by national and global success in reducing newborn deaths.

The previous decade saw major advances in the evidence base for newborn survival, particularly following publication of *The Lancet* neonatal series in 2005 (3). Increasing frequency and reliability of estimates for neonatal mortality and cause of death have led to improved planning and efforts to track progress (3). In addition, there is a growing body of evidence for effective interventions and packages to address the three main causes of newborn mortality (intrapartum-related deaths, complications of prematurity, and severe infections) in weak health systems.

Studies from South Asia have demonstrated that interventions can be delivered cost-effectively using community health workers (CHWs), with a significant impact on neonatal mortality and newborn care practices (4–8). The early positive results of these trials culminated in development of a World Health Organization (WHO)–United Nations Children's Fund joint statement on home visits for newborn care (9). The recommendation was for three visits in the first week of life, with the first visit as soon as possible following delivery. The content of each visit should include examination of the mother and baby for danger signs and promotion of healthy behaviours, including early and exclusive breastfeeding, keeping the baby warm, hand washing, umbilical cord and skin care, identifying conditions requiring additional care, and counselling on when to take a newborn to a health facility (9).

In comparison to the context in much of sub-Saharan Africa, many of the South Asian trials took place in settings with very high baseline neonatal mortality, lower facility delivery rates, lower levels of care-seeking, and established CHW systems. Given these differences, there was a need to test whether these models of care could achieve the same outcomes locally. New studies from Ghana and South Africa have since provided evidence on adapted community-based newborn care packages in Africa, with promising results but less dramatic impact (10, 11).

In Uganda, the community component of the health system is led by volunteers under the national Village Health Team (VHT) strategy (12). The VHT strategy aims to have a team of 5–6 CHWs per village responsible for community mobilisation and preventive care. In 2008, the Ministry of Health began the process of revitalising and expanding the VHT programme to include newborn care and case management for older children, with 1–2 VHT members specifically dedicated to maternal, newborn, and child health. To provide policy makers with information on a scalable package for newborn care in this context, the Uganda Newborn Study (UNEST) was designed as a cluster-randomised controlled trial to evaluate a home visit package with health facility strengthening within existing health system constraints, with the objective of assessing the effect of the UNEST intervention on essential newborn care practices and care-seeking. This is the first article in a supplement reporting on the UNEST results.

## Methods

### Setting

UNEST was implemented in Iganga and Mayuge districts in eastern Uganda, within the Iganga-Mayuge Health and Demographic Surveillance Site (HDSS). The HDSS was established in 2004 in collaboration between the two districts, Makerere University, Uganda, and Karolinska Institutet, Sweden. The HDSS is predominately rural, comprising 65 villages and a total population of approximately 70,000 at the time of the study. Thirteen peri-urban villages form the Iganga Town Council. The main economic activity is subsistence farming. Other occupations include small-scale businesses, such as grain milling, market vending and motorcycle transport, and civil service employment. The predominant ethnic group in the HDSS is the Basoga, a Bantu-speaking group, which makes up 10% of Uganda's population. The HDSS is served by one 100-bed hospital and at least 19 government-run and private-sector health centres that offer delivery services (13). A rising proportion of women in the Central East region – over two-thirds of them – deliver at health facilities (14).

The cluster unit for the study was the village. Each of the 63 villages in the HDSS was randomly allocated to the intervention or control arm, without any stratification or matching due to the relatively large number of study units. Computer-generated restricted randomisation was done in a one-to-one ratio by an independent epidemiologist from the London School of Hygiene and Tropical Medicine. A total of 31 villages were allocated to the intervention arm and 32 to the control arm. More information on the study setting and design are available elsewhere (15).

### Participants and design

The trial included all consenting pregnant women and their newborns residing in the HDSS between September 2009 and August 2011. A team of data collectors linked to the HDSS conducted a baseline household survey to establish coverage of maternal and newborn care behaviours and practices. The baseline survey was conducted between March and August 2008 and included women with a live birth within 4 months of the survey. Information on household asset ownership, care received during pregnancy, childbirth and the postnatal period, and nutritional indicators were collected.

Data for the endline survey were collected between September and November 2011 amongst women who had a live birth within 12 months of the survey (Fig. 1). The primary outcomes of the study were improved coverage of services for antenatal care (ANC), birth preparedness, skilled attendance at delivery, and postnatal care, as well as increases in healthy practices including breastfeeding, thermal care, and hygiene. The study was not powered to detect mortality differences; however, routine birth and death reports were collected as part of household surveillance in the HDSS, but are not reported on here. Prospective data on pregnancies and their outcomes were collected between 2006 and 2010 through routine surveillance in the HDSS. In 2011, a cross-sectional pregnancy history study was conducted amongst 10,540 women aged 15–49, and details are reported elsewhere (16). Village-based scouts notified verbal autopsy interviewers of deaths, including maternal deaths, stillbirths, and neonatal deaths, as they occurred.

**Fig. 1:** UNEST trial profile.

Meetings were held in each district in August 2008 to introduce UNEST and explain the proposed randomisation process to village members and prospective CHWs. The UNEST package was intended to be an integrated intervention package (Fig. 2), based on extensive formative research (17–22), and developed and implemented in close collaboration with national policy makers and experts and the district health management teams of the trial districts. Following a design workshop, the intervention was piloted between November 2008 and February 2009. Building on the pilot, 61 CHWs from the intervention clusters were recruited by the community with the aim of identifying individuals with the following attributes: empathy; experience of similar problems and situations; respected in the local community; and considered to be a natural helper or someone that community members would naturally go to in the event of a problem. Women were preferred, although males were also accepted (women and men can serve as VHT members according to the national strategy).

**Fig. 2:** UNEST conceptual framework. Source: Adapted from Kerber et al. (22).

The CHWs were then trained for 5 days on the intervention package, which included identification of pregnant women in their community and undertaking two home visits during pregnancy and three visits after birth at or as close to days 1, 3, and 7 as possible. Each cluster had at least one CHW, with most villages having two, in line with the national VHT strategy (23), amounting to one CHW per 100–150 households on average. More details, including the selection, training, and supervision of CHWs as well as the content of each visit can be found in the trial protocol (15). After the initial training no additional off-site trainings were conducted, but knowledge and skills were reinforced during quarterly supervisory meetings and through directly observed supervision.

We found this strategy to be effective in imparting and retaining skills (24, 25). CHWs’ incentives were simple, and included a t-shirt, briefcase, certificate, and official commission following their training. The CHWs were not paid a salary by the study, but received a travel refund after supervision meetings.

While UNEST was initially envisioned to be a community-based intervention, the formative research identified relatively high rates of care-seeking at health facilities with low quality care (18). In response, efforts were made to design the intervention to ensure that all 20 public and private health facilities in and around the study area were strengthened through a 6-day in-service training, provision of a once-off catalytic supply of equipment and medicines, as well as collaboration with the district health team to continuously improve the quality of care provided to mothers and newborns (13, 15). Training modules included goal-oriented ANC, managing maternal complications, infection prevention, managing normal labour and partograph use, neonatal resuscitation, care of the sick newborn, and extra care for the small baby using kangaroo mother care. Space for newborn care, including designated kangaroo mother care beds, was set up in the referral sites. Further details of the health system strengthening are provided elsewhere (13). In the comparison villages, women and their newborns had access to the standard health services, overseen by the district health team, in addition to the improved health facilities.

The trial protocol was approved by Makerere University School of Public Health and the Uganda National Council of Science and Technology. In addition, approval was sought from the district authorities and local leaders in the communities where the study was conducted. The study had a data safety monitoring board comprising local and international maternal and newborn experts which met annually. The trial also had a local advisory board which consisted of academics, national policy and programme managers, and development partners. These met quarterly under the auspices of the Uganda National Newborn Steering Committee. The study was registered as randomised controlled trial ISRCTN50321130.

### Statistical analysis

The number of clusters was fixed *a priori* as the existing villages within the HDSS. Study investigators reviewed data collection tools for accuracy and completeness. Data were double entered in databases with consistency and quality checked. We used an intention to treat approach, where we compared summary variables in the intervention and control arms with adjustment for clustering. Statistical analyses were conducted with Stata version 12. We calculated means and proportions of the background characteristics and compared them with *t* tests or *χ* ^2^ tests as appropriate to assess differences at baseline. Using the *svy* command in Stata (version 12), primary sampling units and strata for the data set were defined to account for the cluster-randomised design. At baseline and endline an asset index score was constructed using principal component analysis to rank households according to asset ownership and then divided into quintiles. Details of how the index was built are described elsewhere (19). The effect of the health system strengthening was assessed using a ‘before–after’ comparison.

## Results

### Care during pregnancy and childbirth

Women's age, parity, and household wealth were similar at baseline across the intervention and control arms (Table 1). Women accessing health facilities for ANC and for delivery services increased in both the intervention and control clusters (Table 2). Whereas almost all women attended ANC at least once in both the intervention (98.9%) and control arm (99.2%) (*p*=0.440), slightly more women did so four or more times in the intervention arm (47.9%) compared to the control arm (43.6%) (*p*=0.165). The median number of ANC visits in both arms was 3. The proportion of women who had at least one home visit during pregnancy was 68.2% in the intervention arm compared to 7.3% in the control arm (*p*<0.001). The median number of pregnancy home visits per woman in the intervention arm was 2.4. The median duration of pregnancy at the time of the first CHW home visit was 4.9 months [standard deviation (SD) 1.9], for the second visit 6.8 months (SD 1.4) and for the third visit 8.0 months (SD 1.3). Women in the intervention arm were more likely to report taking actions to prepare for birth compared to those in the control arm (82.9% vs. 72.7%; *p*=0.003), and this practice more than doubled amongst women in both groups over the course of the study.

**Table 1:** Selected baseline characteristics of women by study arm

	Intervention (*n*=194)		Control (*n*=201)	
	
	*n*	%	*n*	%
Age (years)
<19	16	8.3	20	10.0
19–25	62	32.0	69	34.3
26–30	61	31.4	49	24.4
>30	55	28.4	63	31.3
Parity
1–2	71	36.5	77	38.3
3–4	45	23.2	48	23.9
5 or more	78	40.2	76	37.8
Wealth quintile
Lowest	21	10.8	28	13.9
Second	27	13.9	38	18.9
Middle	52	26.8	36	17.9
Fourth	38	19.6	46	22.9
Highest	26	13.4	28	13.9
Missing	30	15.5	26	12.4

Note: Data are means; percentages are based on cluster averages.

**Table 2:** Care during pregnancy and childbirth

	Intervention		Control		
	
	Baseline (*n*=194) %	Endline (*n*=894) %	Baseline (*n*=201) %	Endline (*n*=893) %	*p*^a^
One or more ANC visits	93.8	98.9	94	99.2	0.440
Four or more ANC visits	29.9	47.0	28.9	43.6	0.165
Prepared for birth^b^	32	82.9	30.9	72.7	0.003
Knowledge of two or more pregnancy-related danger signs	41.8	32.7	41.8	38	0.126
One or more home visit during pregnancy	–	68.2	–	7.3	<0.001
Delivered in health facility	70.6	78.0	68.7	77.7	0.939
Skilled attendant at delivery	58.2	79.6	59.7	78.9	0.826

Note: Percentages based on cluster averages.

a *P*-values calculated with *t*-test to compare differences between intervention and control clusters at endline.

b Birth preparedness refers to acquiring gloves, plastic to deliver on, instruments for cutting and tying cord, cotton wool, and saving money for transport and fees.

The proportion of women who reported having a skilled attendant at delivery did not differ between the intervention and control arm at endline (79.6% vs. 78.9%; *p*=0.826). However, this proportion increased by 21.3 and 19.2% compared to baseline in the intervention and control arm, respectively, and came entirely from increases in use of facilities in the public sector. There was a 13% before–after decrease in women from intervention clusters who reported giving birth in private health facilities (from 29.9 to 16.7%), compared to a 2.5% reduction in the control arms (from 20.4 to 17.9%). Similarly, the use of traditional birth attendants (TBAs) dropped by 5.7% in the intervention arm but remained stagnant in the control arm. Clean delivery practices were high in both arms and did not differ significantly between the intervention and control group.

### Postnatal care contact and practices

Overall 62.8% of the women in the intervention arm were visited by a CHW in the first week after birth compared to 5.8% in the control arm (*p*<0.001). More women who delivered at home and with a TBA were visited by a CHW after birth (73.6%) compared to those who delivered in a hospital or health facility (59.7%) (*p*<0.001) (Table 3).

**Table 3:** Home visits in first week after birth by place of delivery in the intervention arm (*n*=894)

	*n* (%)	*p*^a^
Amongst all births, at least one home visit in the first week after birth	894	
Yes	561 (62.8)	
No	332 (37.1)	
Don't know/missing	1 (0.1)	
Amongst those with at least one home visit in the first week after birth	561	
Babies born at a health facility	416 (59.7)	
Babies born with a TBA/home	145 (73.6)	0.002
Amongst those whose first home visit took place on day 1	228	
Babies born at a health facility	161 (23.4)	
Babies born at home or with a TBA	67 (34.2)	0.004
Women counselled on well baby practices by a CHW after birth, of those who received a home visit	373 (66.5)	
Women counselled on well baby practices by a CHW after birth		
Babies born at a health facility	305 (54.8)	
Babies born at home or with a TBA	64 (32.7)	0.002

Note: Percentages based on cluster averages in the intervention clusters only.

a Calculated with *t*-test (intervention arm vs. control arm at endline).

Of the 561 women in the intervention arm who had a visit by a CHW in the first week after birth, 228 (40.6%) had their first visit on day 1, and only 11.8% or 66 women had their first visit on day 7. Women were more likely to receive a home visit in the first week if they delivered at home (73.6%) compared to those who delivered in a health facility (59.7%) (*p*<0.002). By day 7, the proportion of women visited by a CHW did not differ by place of delivery. Amongst women who gave birth in a health facility and received a CHW home visit in the first week, only 8.2% were visited first on day 1 and 30% were seen on day 2, suggesting that birth in a health facility is associated with a delayed CHW home visit, even though 70% of women were discharged from the facility within 24 hours of delivery.

The proportion of babies who were breastfed within the first hour after birth was significantly higher in the intervention arm compared to the control arm (72.6% vs. 66.0%; *p*=0.0116) (Table 4). This practice increased by 20% from baseline in both arms. The proportion of babies who were exclusively breastfed in the neonatal period was also significantly higher in the intervention arm (81.8%) compared to the control arm (75.9%) (*p*=0.042). Exclusive breastfeeding increased from baseline by 14% and 20% in the intervention and control arm, respectively. The proportion of babies placed skin-to-skin with their mothers immediately after birth was higher in the intervention arm (80.7% vs. 72.2%; *p*=0.071) and amongst those born in health facilities than those born at home or elsewhere.

**Table 4:** Early postnatal practices related to breastfeeding, thermal care, and hygiene

	Intervention				Control				
	
	Baseline (*N*=194)		Endline (*N*=894)		Baseline (*N*=201)		Endline (*N*=893)		
			
	*n*	%	*n*	%	*N*	%	*n*	%	*p*^a^
Optimal feeding
Baby put to the breast within 1 hour of birth	101	52.1	647	72.6	94	46.8	586	66.0	0.016
Baby given colostrum	b	–	835	93.4	b	–	814	91.2	0.086
Baby exclusively breastfed in first month of life	130	67.0	731	81.8	114	56.7	678	75.9	0.042
Thermal protection
Baby placed skin-to-skin with mother within 1 hour of birth	b		721	80.7	b		663	74.2	0.071
Baby dried immediately after birth	b		882	98.7	b		873	97.8	0.754
Baby wrapped immediately after birth	160	82.5	890	99.6	171	85.1	891	99.8	0.562
First bath delayed ≥6 hours after birth	63	32.5	824	92.2	40	19.8	765	85.7	<0.001
First bath delayed ≥24 hours after birth	4	2.06	443	49.6	4	1.99	317	35.5	<0.001
Hygienic care
Cord cut with clean instrument^c^	100	51.6	788	88.1	118	58.7	754	84.4	0.074
Nothing applied to umbilical cord after cutting	81	41.6	571	63.9	114	56.7	474	53.1	0.002

Note: Percentages based on cluster averages.

a Calculated with *t*-test (intervention arm vs. control arm at endline).

b Indicator not collected in baseline survey.

c New blade or boiled blade.

Women in intervention villages reported following better thermal care practices than their counterparts in control areas (Table 4). Almost half (49.6%) of the mothers in the intervention group delayed the baby's first bath for at least 24 hours, compared to 35.5% in the control arm (*p*<0.001). This proportion increased by 47% and 33% between baseline and endline in the intervention and control arms, respectively. The proportion of babies whose bath was delayed for more than 6 hours was also significantly higher in the intervention arm at 92.2% versus 85.7% in the control arm (*p*<0.001). The delayed bathing practice increased by more than 50% in both arms, with no difference in delayed bathing according to place of delivery. Most babies were dried immediately after birth, and this practice was equally high (98.7 and 97.8%) in the intervention and control arms and did not vary between babies born at health facilities or at home. Similarly, the practice of wrapping the baby immediately after delivery was high at baseline but still increased by more than 15% in both arms.

Hygienic cord care practices were better amongst families in the intervention clusters. The proportion of babies whose cord was cut with a clean instrument (new razor blade or boiled tool) was higher in the intervention arm compared to the control arm (88.1% vs. 84.4%; *p*=0.074), but not significantly so (Table 4). The practice increased over the course of the intervention by more than 35% in both arms. The proportion of babies who had nothing put on the umbilical cord stump was 63.9% in the intervention arm, significantly higher than in the control arm at 53.1% (*p*=0.002). This practice increased by 22% from baseline in the intervention arm, whereas it decreased by 3% in the control arm.

Almost all babies reported to have experienced at least one danger sign were taken outside the home for care (95.0% in the intervention arm and 94.1% in the control arm). More than half of all mothers with sick newborns went to a private provider, with drug shops being the first point of care for 24.9 and 28.0% in the intervention and control arms, respectively (*p*=0.412). A total of 162 babies (9.1%) were born with low birthweight (LBW) based on either documentation of baby's weight at birth or the mother's perception that her baby was very small or smaller than average. A significantly higher proportion of LBW babies in the intervention arm were given kangaroo mother care compared to those in the control arm (22.4% vs. 9.3%; *p*=0.089). The proportion of mothers who recognised LBW as a danger sign and sought extra care because of the small size of their baby was higher in the intervention than in the control arm, but the difference was not significant (23.7% vs. 14.0%; *p*=0.234).

### Home visits and CHW workload

Overall CHWs made a total of 4,772 home visits. Each CHW saw an average of 26 mothers per year and made 78 home visits, or 1.5 home visits per week. Each mother and baby received on average 3 of the targeted 5 home visits. The number of CHW visits to mothers with small babies compared to normal weight babies did not vary significantly, implying that LBW babies did not get extra visits – contrary to what the intervention promoted. The average length of a home visit was 82 min, with no significant difference in the time spent by CHWs during either pregnancy or postnatal home visits. Retention of CHWs was 100% during the study implementation period.

## Discussion

Over 2 years of implementation, UNEST achieved significant improvements in birth preparedness and essential newborn care practices, including breastfeeding, hygienic cord care, and thermal protection – practices associated with reduced neonatal mortality. These and other interventions, including ANC and skilled attendance at delivery, recorded increases between baseline and endline. The general improvement in maternal and newborn care practices across both the intervention and control arms may be explained at least in part by the health facility strengthening which impacted both trial arms, but also by the secular trend towards improved maternal and newborn care. CHWs selected by their communities with district-led training and supervision were able to identify and visit almost all the pregnant women, especially those from the poorest families and those who delivered at home or with TBAs. These results have important policy implications in Uganda and in similar settings where CHW programmes for maternal and newborn care are being designed or scaled up.

These findings demonstrate the power of CHWs to effect change in behaviours around maternal and newborn care, and are similar to those reported elsewhere (10, 26). Of the 10 mother-led interventions (birth preparedness, 3 practices related to optimal feeding, 4 thermal care practices, and 2 hygienic cord care practices), 8 reached over 80% coverage in the intervention area. For the intervention and control clusters combined, coverage of the seven practices measured at both baseline and endline was an average of 29% higher post-intervention, demonstrating increased awareness of and demand for newborn care. Contrary to expectations, we found improvements even in practices that formative research indicated may be difficult to change, such as delaying bathing. We are confident that most of the changes observed are mainly due to the CHW intervention, although we are aware some are due to the health facility strengthening, and delivery care is mainly due to a secular trend towards improved institutional births. However, while dry cord care increased and was significantly better in intervention clusters, this coverage remained lowest of all essential newborn care practices, suggesting challenges in improving it. Recent WHO and national recommendations on application of topical antiseptics such as chlorhexidine to the umbilical cord stump (27, 28) may replace the application of common harmful substances, and could be integrated into the CHW messaging during pregnancy and postnatal visits.

Complications of preterm birth are now the second leading cause of all child deaths globally, and the third leading cause in Uganda, after malaria and pneumonia (29). CHW home visits were associated with babies born with LBW receiving kangaroo mother care, demonstrating that awareness during pregnancy is key. In the hospital where kangaroo mother care was introduced, 85% of the 547 babies admitted to the unit were discharged alive (13). Still, this low-tech, caregiver-led intervention reached fewer than 1 in 5 preterm babies. CHWs did not manage to make extra visits to LBW babies, contrary to their training. In order to maximise scalability and limit procurement, the CHWs were not equipped with weighing scales and lacked a reliable mechanism to identify these small babies, particularly those born at home and not weighed at birth. A study nested within UNEST validated a foot length card for use by CHWs (30), which has since been taken up through the national VHT strategy. Future CHW studies must have special attention to care for small babies as a critical part of newborn care.

Care-seeking for routine and extra care services increased in both the control and intervention arms, with an increase in demand for public sector pregnancy and delivery services. ANC and institutional deliveries increased overall, but the proportion of women giving birth at private facilities decreased, with most of the decline amongst women in the intervention arm. Care-seeking for sick newborns was much higher than seen in other settings, but echoes qualitative research indicating that compliance with referral by VHTs is high (31, 32). In contrast to the more common public sector delivery services, private care, mainly through small-scale drug shops, was the first point of service for sick newborns. An assessment of essential newborn care in private facilities in the UNEST areas demonstrated that private health facilities did not perform significantly better than public health facilities, despite the additional cost of these services (33). After facility strengthening, including training and support to management, equipment levels remained high – but maintaining supply of even the most basic medications was a challenge, with less than 40% of health facilities reporting no stock-outs (13). While government engagement is necessary to maintain quality public sector services, strategies are also needed to engage and ensure accountability within the private sector.

Achievements in improved newborn care practices were realised with modest CHW effectiveness in carrying out the intended visit schedule. Only 54.0% of the women in the intervention arm received two or more visits during pregnancy and only 62.8% received a home visit from a CHW in the first week after birth. Even though women were discharged from health facilities within 24 hours, CHWs saw these women later on average than women who delivered at home, perhaps due to lack of notification of delivery and their return home. Mothers in the poorest wealth quintiles were more likely to benefit from these early visits, as were those who delivered at home or with TBAs. While this indicates a desirable pro-poor emphasis, the trend towards increasing institutional deliveries nationally and throughout much of the continent necessitates a platform that works for all women, with stronger communication links to the facilities where women give birth and bring their newborns for care. Here, m-health interventions could be useful as communication tools that allow for support to families at a distance.

While a trial in this well-defined geographical area may represent an efficacy trial, many features of the intervention strategy appear to be scalable with no loss of effectiveness. UNEST utilised district structures to select, train, and supervise CHWs, rather than study staff. Both CHWs and their supervisors did not receive designated salaries, but travel refunds and stipends at a more scalable cost. The cost per mother visited (all visits) stood at US $25 and per home visit at US $8.30. The cost of CHWs is affordable and reduces with scale-up, because the initial set-up costs are not repeated. However, CHW retention was high and more recruitment and retraining would be required over the long term.

The quality and frequency of supervision that can be given by the health workers may vary by setting, and should be taken into consideration. The CHWs hired for UNEST were recruited anew because revitalisation of the national VHT strategy was just beginning when UNEST started. While UNEST CHWs did not have additional responsibilities, their home visit load was generally low and conducive to integration into the broader maternal, newborn, and child health role of the VHTs. Incentivisation and CHW motivation needs to be carefully considered, as reflected in another article in this series (34). To this end the UNEST training package, home visit schedule and behaviour change counselling materials were incorporated into the national VHT and Integrated Community Case Management packages while the study was taking place (35).

One limitation of this study is the differing recall periods of the baseline (4 months) and endline (12 months) surveys for services that occurred before and around the time of birth, although large-scale household surveys include longer recall periods of up to 5 years. An additional limitation is that these surveys only captured women who had live births in both the baseline and endline surveys. The pregnancy home visits, birth-preparedness counselling, and facility quality of care improvements are likely to have had an impact on stillbirths as well as neonatal deaths.

The study was not powered to measure an effect on mortality, although improvements in coverage of care are similar to those seen with a modest mortality impact (10). While the HDSS allowed for documentation of the number and causes of newborn deaths, it was apparent that pregnancy outcomes were being missed in the two census rounds per year. Further analysis and validation of mortality data from household surveys is on-going.

While the improvements in care indicators in both arms could be an effect of the health system strengthening, it could also be an effect of spill-over of the knowledge from one intervention village to the next control village, since there were no buffer villages between intervention and control areas, or a secular trend in Uganda which needs to be corroborated by further research. The scope for potential increase in some of the preventive behaviours was low, because the coverage of these behaviours was already high.

## Conclusion

National attention to newborn survival and health has increased, with UNEST strategically placed to influence key policies and strategies linked to the national VHT strategy and improvements in quality of care at health facilities. Home visits from CHWs are associated with improved essential newborn care practices, regardless of place of delivery. However, there is a key concern around a mismatch between utilisation and quality that results in avoidable deaths.

Additional efforts to prevent the three main causes of neonatal deaths, particularly complications of preterm birth, are needed, linking community efforts to facility quality of care improvement in both the public and private sector. National attention and policies are necessary but not sufficient steps to save newborn lives as well as to prevent maternal deaths and stillbirths. Closing the policy–practice gap at district level is needed to improve maternal and newborn survival and health.

## References

[1] UNICEF, WHO, The World Bank, United Nations (2013). Levels and trends in child mortality: report 2013.

[2] Cousens S, Blencowe H, Stanton C, Chou D, Ahmed S, Steinhardt L (2011). National, regional, and worldwide estimates of stillbirth rates in 2009 with trends since 1995: a systematic analysis. Lancet.

[3] Lawn JE, Kinney MV, Black RE, Pitt C, Cousens S, Kerber K (2012). Newborn survival: a multi-country analysis of a decade of change. Health Policy Plan.

[4] Bang AT, Reddy HM, Deshmukh MD, Baitule SB, Bang RA (2005). Neonatal and infant mortality in the ten years (1993 to 2003) of the Gadchiroli field trial: effect of home-based neonatal care. J Perinatol.

[5] Kumar V, Mohanty S, Kumar A, Misra RP, Santosham M, Awasthi S (2008). Effect of community-based behaviour change management on neonatal mortality in Shivgarh, Uttar Pradesh, India: a cluster-randomised controlled trial. Lancet.

[6] Baqui AH, Arifeen SE, Williams EK, Ahmed S, Mannan I, Rahman SM (2009). Effectiveness of home-based management of newborn infections by community health workers in rural Bangladesh. Pediatr Infect Dis J.

[7] Darmstadt GL, Choi Y, Arifeen SE, Bari S, Rahman SM, Mannan I (2010). Evaluation of a cluster-randomized controlled trial of a package of community-based maternal and newborn interventions in Mirzapur, Bangladesh. PLoS One.

[8] Bhutta ZA, Soofi S, Cousens S, Mohammad S, Memon ZA, Ali I (2011). Improvement of perinatal and newborn care in rural Pakistan through community-based strategies: a cluster-randomised effectiveness trial. Lancet.

[9] WHO, UNICEF, USAID, Children, St. WHO-UNICEF Joint Statement (2009). Home visits for the newborn child: a strategy to improve survival.

[10] Kirkwood BR, Manu A, ten Asbroek AH, Soremekun S, Weobong B, Gyan T (2013). Effect of the newhints home-visits intervention on neonatal mortality rate and care practices in Ghana: a cluster randomised controlled trial. Lancet.

[11] Tomlinson M, Doherty T, Ijumba P, Jackson D, Lawn J, Persson LA (2014). Goodstart: a cluster randomised effectiveness trial of an integrated, community-based package for maternal and newborn care, with prevention of mother-to-child transmission of HIV in a South African township. Trop Med Int Health.

[12] Mbonye AK, Sentongo M, Mukasa GK, Byaruhanga R, Sentumbwe-Mugisa O, Waiswa P (2012). Newborn survival in Uganda: a decade of change and future implications. Health Policy Plan.

[13] Namazzi G, Waiswa P, Nakakeeto M, Nakibuuka VK, Namutamba S, Najjemba M (2014). Strengthening health facilities for maternal and newborn care: experiences from rural eastern Uganda. Glob Health Action.

[14] Uganda Bureau of Statistics (UBOS), Macro International Inc (2011). Uganda demographic and health survey 2010.

[15] Waiswa P, Peterson SS, Namazzi G, Ekirapa EK, Naikoba S, Byaruhanga R (2012). The Uganda Newborn Study (UNEST): an effectiveness study on improving newborn health and survival in rural Uganda through a community-based intervention linked to health facilities – study protocol for a cluster randomized controlled trial. Trials.

[16] Kadobera D (2014). Experiences of identifying pregnant women and pregnancy outcomes in Iganga-Mayuge Health and Demographic Surveillance Site. Glob Health Action.

[17] Waiswa P, Nyanzi S, Namusoko-Kalungi S, Peterson S, Tomson G, Pariyo GW (2010). ‘I never thought that this baby would survive; I thought that it would die any time’: perceptions and care for preterm babies in Eastern Uganda. Trop Med Int Health.

[18] Waiswa P, Kallander K, Peterson S, Tomson G, Pariyo GW (2010). Using the three delays model to understand why newborn babies die in Eastern Uganda. Trop Med Int Health.

[19] Waiswa P, Peterson S, Tomson G, Pariyo GW (2010). Poor newborn care practices – a population based survey in Eastern Uganda. BMC Pregnancy Childbirth.

[20] Larsson EC, Waiswa P, Thorson A, Tomson G, Peterson S, Pariyo G (2009). Low uptake of HIV testing during antenatal care: a population-based study from Eastern Uganda. AIDS.

[21] Waiswa P, Kemigisa M, Kiguli J, Naikoba S, Pariyo GW, Peterson S (2008). Acceptability of evidence-based neonatal care practices in rural Uganda – implications for programming. BMC Pregnancy Childbirth.

[22] Kerber KJ, de Graft-Johnson JE, Bhutta ZA, Okong P, Starrs A, Lawn JE (2007). Continuum of care for maternal, newborn, and child health: from slogan to service delivery. Lancet.

[23] Health Education and Promotion Division (2010). Village Health Team strategy and operational guidelines.

[24] Nalwadda CK, Waiswa P, Kiguli J, Namazzi G, Namutamba S, Tomson G (2013). High compliance with newborn community-to-facility referral in Eastern Uganda: an opportunity to improve newborn survival. PLoS One.

[25] Kayemba Nalwadda C, Guwatudde D, Waiswa P, Kiguli J, Namazzi G, Namutumba S (2013). Community health workers – a resource for identification and referral of sick newborns in rural Uganda. Trop Med Int Health.

[26] Prost A, Colbourn T, Seward N, Azad K, Coomarasamy A, Copas A (2013). Women's groups practising participatory learning and action to improve maternal and newborn health in low-resource settings: a systematic review and meta-analysis. Lancet.

[27] Hodgins S, Pradhan YV, Khanal L, Upretti S, Pratap KCN (2013). Chlorhexidine for umbilical cord care: game-changer for newborn survival?. Glob Health Sci Pract.

[28] WHO Guidelines Review Committee (2013). Guidelines on maternal, newborn, child, and adolescent health: Recommendations on newborn health.

[29] UNICEF and WHO (2014). Fulfilling the health agenda for women and children: countdown to 2015 for maternal, newborn and child survival.

[30] Nabiwemba E, Marchant T, Namazzi G, Kadobera D, Waiswa P (2013). Identifying high-risk babies born in the community using foot length measurement at birth in Uganda. Child Care Health Dev.

[31] Nalwadda Kayemba C, Naamala Sengendo H, Ssekitooleko J, Kerber K, Kallander K, Waiswa P (2012). Introduction of newborn care within integrated community case management in Uganda. Am J Trop Med Hyg.

[32] Nalwadda CK, Waiswa P, Guwatudde D, Kerber K, Peterson S, Kiguli J (2014). ‘As long as the umbilical cord gets off, the child ceases to be called a newborn’: sociocultural beliefs and newborn referral in rural Uganda. Glob Health Action.

[33] Waiswa P, Akuze J, Peterson S, Kerber K, Tetui M, Forsberg BC (2014). Differences in essential newborn care at birth between private and public health facilities in eastern Uganda. Glob Health Action.

[34] Okuga M, Kemigisa M, Namutamba S, Namazzi G, Waiswa P (2014). Engaging community health workers in maternal and newborn care in eastern Uganda. Glob Health Action.

[35] Waiswa P, Namazzi G, Kerber K, Peterson S (2014). Designing for action: adapting and implementing a community-based newborn care package to affect national change in Uganda. Glob Health Action.

# Sub article: Designing for action: adapting and implementing a community-based newborn care package to affect national change in Uganda

**DOI:** 10.3402/gha.v8.24250

**Published:** 2015-03-31

**Authors:** Peter Waiswa, Gertrude Namazzi, Kate Kerber, Stefan Peterson

**Affiliations:** 1 Department of Health Policy, Planning and Management, School of Public Health, College of Health Science, Makerere University, Kampala, Uganda; 2 Global Health, Department of Public Health Sciences, Karolinska Institutet, Stockholm, Sweden; 3 Saving Newborn Lives, Save the Children, Cape Town, South Africa; 4 International Maternal and Child Health Unit, Department of Women's and Children's Health, Uppsala University, Uppsala, Sweden

**Keywords:** newborn health, maternal health, community health worker, pregnancy, postnatal care, Uganda, formative research, health policy

## Abstract

**Background:**

There is a lack of literature on how to adapt new evidence-based interventions for maternal and newborn care into local health systems and policy for rapid scale-up, particularly for community-based interventions in low-income settings. The Uganda Newborn Study (UNEST) was a cluster randomised control trial to test a community-based care package which was rapidly taken up at national level. Understanding this process may help inform other studies looking to design and evaluate with scale-up in mind.

**Objective:**

This study aimed to describe the process of using evidence to design a community-based maternal and newborn care package in rural eastern Uganda, and to determine the dissemination and advocacy approaches used to facilitate rapid policy change and national uptake.

**Design:**

We reviewed UNEST project literature including meeting reports and minutes, supervision reports, and annual and midterm reports. National stakeholders, project and district staff were interviewed regarding their role in the study and perceptions of what contributed to uptake of the package under evaluation. Data related to UNEST formative research, study design, implementation and policy influence were extracted and analysed.

**Results:**

An advisory committee of key players in development of maternal and newborn policies and programmes in Uganda was constituted from many agencies and disciplines. Baseline qualitative and quantitative data collection was done at district, community and facility level to examine applicability of aspects of a proposed newborn care package to the local setting. Data were summarised and presented to stakeholders to adapt the intervention that was ultimately tested. Quarterly monitoring of key activities and events around the interventions were used to further inform implementation. The UNEST training package, home visit schedule and behaviour change counselling materials were incorporated into the national Village Health Team and Integrated Community Case Management packages while the study was ongoing.

**Conclusions:**

Designing interventions for national scale-up requires strategies and planning from the outset. Use of evidence alongside engagement of key stakeholders and targeted advocacy about the burden and potential solutions is important when adapting interventions to local health systems and communities. This approach has the potential to rapidly translate research into policy, but care must be taken not to exceed available evidence while seizing the policy opportunity.

Community-based strategies for maternal and newborn health may prevent up to one-third of the world's annual 2.9 million newborn deaths if universal coverage is achieved (1). A number of community-based trials from Asia demonstrated the potential of task shifting to community health workers (CHWs) for newborn care (2–6), leading to a 2009 Joint Statement on Home Visits for Newborn Care from the World Health Organization (WHO), United Nations Children's Fund (UNICEF) and other partners (7). The recommendations in the Joint Statement were taken up by many countries, including 20 out of 46 countries in sub-Saharan Africa (8). However, few studies have evaluated the implementation effectiveness of home visits for newborn care, and only one study in Ghana has reported on both the process of adaptation (9) and evaluation of the package (10).

Closing the gap from research to practice requires adapting interventions and packages to local settings (11). This is particularly true for interventions that must address strongly held beliefs and practices and tackle complex cultural barriers to care-seeking. There is a lack of guidance in customising interventions for the critical pregnancy, childbirth and postnatal periods. Designing a new package of care requires local contextual information on existing practices and knowledge of the key influentials (12). It is difficult to know in advance how much adaptation of a trial-tested intervention or health system package will be necessary while still maintaining fidelity of the intervention and key elements which play a role in the pathways to behaviour change and ultimately mortality reduction.

To help address this gap in knowledge, the Uganda Newborn Study (UNEST) was designed as a two-arm cluster randomised control trial, taking place in two rural districts in eastern Uganda (13). In intervention, villages volunteer CHWs were trained to identify pregnant women and make five home visits (two during pregnancy and three in the first week after birth) to offer preventive and promotive care and counselling, with extra visits for sick and small newborns to assess and refer. Health facility strengthening was done in all facilities to improve quality of care. Primary outcomes were coverage of key essential newborn care behaviours (breastfeeding, thermal care and cord care). The intervention significantly improved essential newborn care practices, although many key behaviours and practices saw major increases in both arms over the study period. The home visit package was pro-poor, with more women in the poorest quintile visited by a CHW compared to families in the least poor quintile, and more women who delivered at home visited by a CHW after birth compared to those who delivered in a hospital or health facility.

We describe how evidence was used to design and implement this home visit and health facility strengthening strategy for newborn care through UNEST, and how this led to rapid policy and programme uptake, which began influencing scale-up in the country even before the trial was completed. This paper is the second in a series on the UNEST results.

## Design

As a qualitative analysis to determine factors resulting in diffusion and uptake of key elements of the UNEST cluster randomised control trial, we reviewed UNEST internal project literature. This included project reports, meeting minutes, decision trees, supervision reports, and midterm and final reports. In addition, we reviewed and categorised the range of published literature linked to UNEST, including peer-reviewed articles, information brochures, newspaper articles and video documentaries. In-depth interviews were conducted with all members of the UNEST Advisory Committee (*n*=6), conducted by an experienced social scientist with the help of a note-taker. Interviews took place at the respondent's place of work, and focused on understanding current policy, challenges, opportunities and perceptions of stakeholder willingness to adopt Asian models of newborn care.

We also interviewed project and district staff, as well as those involved in newborn care at national level and international experts involved in UNEST design and implementation. Using the WHO and ExpandNet framework on designing projects with scaling-up in mind (14), we analysed the steps and decisions taken based on existing evidence outside Uganda and the local context, particularly in relation to the initial design workshop. Activities, products, key milestones and decisions were plotted on a timeline and analysed thematically.

## Results

The development of the UNEST package followed a linear process, starting with a literature review which helped define the knowledge gaps and local needs. Baseline data collection and formative research were undertaken to describe the current situation in maternal and newborn health and potential barriers to uptake of behaviours and practices. Based on this information, a context-specific package was developed and pilot tested, with refinements made before full implementation. Stakeholder participation at each stage of this process as well as creative engagement of various dissemination outlets enabled rapid adoption and uptake of the package.

### Literature review and package parameters

One of the objectives of UNEST was to adapt India's SEARCH home visit model (2) for use in Uganda. A literature review was done mainly focusing on community newborn interventions, mainly in Asian settings. At the time, the recent release of *The Lancet* Neonatal Series was instrumental in framing the package in terms of the overall burden and causes of death, the survival benefit associated with each intervention, affordability and prioritising community-based care (15, 16). In addition, we explored the Uganda-specific literature and guidelines around maternal and newborn care.

Two major contextual differences were acknowledged arising out of the Ugandan literature. The first was the existence of the historical Village Health Team (VHT) system, which provided a platform for CHW recruitment without the need for creation of a new cadre. The team dynamic of 5–6 members with one focused on maternal and child health was not seen elsewhere. Secondly, dissimilar to the SEARCH model, it was decided that the package would not include sepsis case management at community level, given current clinical guidelines and perceived accessibility of existing curative health services to families. There was considerable discussion and negotiation on this point. The UNEST design phase overlapped with a review of the national Essential Medicines List, which had the potential to include injectable antibiotics for newborn sepsis at the lower-level health facilities, but at the time this intervention was not thought to have sufficient evidence to warrant a significant change in policy.

We also assessed policies and guidelines with regard to community mobilisation and creating demand for maternal, newborn and child health services, drawing heavily on previous work done on breastfeeding counselling and promotion (17–19). There was a notable gap here with a lack of community awareness of and demand for services for preterm and low-birthweight babies. Prior to the UNEST pilot, a network of researchers involved in seven community-based newborn care studies in Ethiopia, Ghana, Malawi, Mozambique, South Africa, Tanzania and Uganda were convened by Save the Children's Saving Newborn Lives programme. The network met in 2006 and 2007 to discuss design issues and formative research, and again in 2009 to review results of the pilot phase and address implementation and monitoring issues. Based on the review of evidence as well as discussions with policy makers and technical experts, it was decided that the package would include preventive home visits in pregnancy and the first week of life using the VHTs linked to the existing district health teams (DHTs).

In order to promote sustainability and potential for replication, policy makers requested that the intervention not include home treatment for neonatal sepsis, community weighing of babies, or taking the baby's temperature. Instead, they recommended strengthening community-facility linkages and referral of sick babies or babies born at home to health facilities.

### Baseline data collection and formative research

Baseline field data collection involved qualitative and quantitative approaches. Formative research built on a similar process and questions used in that underpinning the Newborn Home Intervention Study (Newhints) in the Brong Ahafo region of Ghana (9) (Table 1). Objectives of the formative research were multifaceted, and included:

1. To explore the community's knowledge and perceptions on home care practices, response to neonatal danger signs, care-seeking behaviour and barriers to key practices and care in order to inform behaviour change communication materials and strategies;
2. To investigate health system and socio-economic factors associated with neonatal deaths and the quality of maternal and newborn basic and emergency care in health facilities in order to inform health system strengthening; and
3. To explore the feasibility and acceptability of different supervision and motivation models for CHW visits to pregnant women and newly delivered mothers, and strategies to better link households to health care.

In-depth interviews were conducted with health managers, health workers and community members to understand perceptions of quality and accessibility of maternal and newborn health services and potential barriers and facilitators for behaviour change around key practices.

**Table 1:** Questions to be answered by the formative research and links to intervention design

Aim	Areas of focus	Link to intervention design
Identify gaps in family knowledge and practice of neonatal care	Which priority newborn care behaviours are currently practised at household level and which are not?	Identify priority practices to promote during home visits
Identify causes of newborn death and modifiable factors	What are the biological causes of newborn deaths locally? Which are the social contributors to newborn death? How can these be overcome?	Ensure interventions address the biological causes, social causes and underlying delays related to newborn deaths
Identify whether certain behaviours are more or less likely to be changed	Can the key behaviours be changed? Which resources are available and which are not? Who are the key influencers What are the best channels for facilitating behaviour change?	Identify priority practices for the intervention; identify delivery channels and target audience
Characterise CHWs: availability, role in proposed intervention and their management	Are there CHWs currently? Who are they? How are they selected? What do they do? What caseload is feasible? Can CHWs facilitate problem solving? Are they generally accepted? How can CHWs be motivated?	Guide CHW selection, motivation and supervision
Explore how the intervention logistics can be managed	How can pregnant women be identified and at what stage of pregnancy? Is it possible to visit all women at home during pregnancy and after birth? Is it appropriate for TBAs to serve as CHWs?	Guide identification of primary targets
What is the current quality of maternal and neonatal health services?	Which equipment, supplies and skills are available for neonatal care in health facilities? What causes of death explain the current in-facility mortality?	Guide health facility strengthening and supply-side intervention
Explore how referral can be ensured	Can pregnant women and newly delivered mothers and newborns be referred from community to local health centre when sick? How can linkages with facilities be ensured? What are the current barriers to referral and how can these be overcome?	Guide design of strategies to ensure compliance with referral

Quantitative data collection at baseline involved review of verbal autopsy data from the Iganga-Mayuge Health and Demographic Surveillance Site (HDSS) in order to understand the local causes of newborn deaths and contributing delays (20). In addition, a population-based cross-sectional study was done to establish the baseline levels of maternal and newborn care among 395 newly delivered mothers with babies aged 1–4 months in the HDSS. We assessed the knowledge of maternal and newborn care of 52 health workers using a standardised questionnaire. We also conducted an assessment in 16 health facilities in and surrounding the HDSS to determine the availability of infrastructure, human resources, records, medicines and equipment important for neonatal survival. The facility assessment survey also aimed to identify whether practices and messages were consistent with desired behaviours and whether the quality of care needed to be improved.

The formative findings and baseline data have been published elsewhere (20–23). Table 2 summarises the key findings from the formative research and how this was used to design the intervention. Briefly, some weaknesses were found in terms of both demand for and supply of service, and the linkages between the two were weak. Verbal and social autopsy studies identified major delays at home and in the community associated with stillbirths and neonatal deaths, but also significant limitations in the health facility capacity for newborn care. While community home visits remained the major focus of the trial, based on these gaps a key decision was made to implement and monitor a substantial health systems strengthening component at health facility level. The formative research identified supervision and motivation of CHWs as a critical barrier to address, especially amongst those CHWs previously employed by other projects. Interviews revealed that the CHWs in operation at baseline felt that the work was tiring and time-consuming, and were dissatisfied with the supervision and motivation received for their work (on average 2 days per week).

**Table 2:** Interventions designed to address gaps identified in the formative research

Desired behaviour or practice	Gap identified in formative research	Intervention to address the gap	Context-specific adaptation
Every pregnant woman attends ANC at least 4 times	Only 30% of women had 4 or more ANC visits	CHWs would be trained and supported to identify p. early and provide health education to promote regular ANC attendance	Single-visit ANC coverage already high but extra emphasis placed on importance of multiple visits Extra effort made to identify pregnant women early and track identified vs expected pregnancies to make sure CHWs were capturing women who might otherwise not attend ANC, or would attend late
Every family prepares for birth	Lack of preparation for birth	CHWs would be trained and supported to identify pregnancy early and support family in birth preparation	Lack of materials for birth identified as key barrier for receiving care; mama kits and saving money targeted as key messages during counselling
Every woman delivers in a health facility with a skilled attendant	Only 42% of women delivered from a health facility	CHWs would be trained and supported to identify pregnant women and promote health facility delivery as part of birth preparedness	Increasing demand through CHW visits alone did not seem sufficient to bridge this gap; health facility strengthening component developed largely in response to quality of care concerns at facility
Every woman is aware of maternal and neonatal danger signs	Only 35% of women knew at least 3 maternal danger signs; 48% knew at least 3 newborn danger signs	CHWs would be trained and supported to provide health education regarding maternal and newborn danger signs and care-seeking	Local concepts and terms were used to describe danger signs and to differentiate clinical signs of severe illness from traditional illnesses considered to be severe, e.g. false teeth
Every newborn receives immediate essential care	Babies receiving: Optimal thermal care: 42% Optimal neonatal feeding: 57% Optimal cord care: 38%	CHWs would be trained and supported to counsel on immediate newborn care during pregnancy visits, identify newly delivered mothers and visit them at home on day 1, 3 and 7 and support postnatal care	Given the high facility delivery rate, the strategy to reach mothers and babies on day 1 focused on home deliveries with an attempt to connect the health facility and CHW with information that the pair had been discharged
Every woman and baby receives quality care on a continuum from home to health facility, and back	Of those babies that died, 50% were related to delays at home, and 30% were related to delays in receiving care at health facilities Only 30% of health workers had knowledge of newborn care Health facilities lacked equipment, drugs and supplies for newborn care	CHWs would be trained and supported to identify newly delivered mothers, support postnatal care, screen for danger signs and facilitate referral Health facilities strengthened through training, provision of basic drugs and equipment, and maternal and perinatal audit introduced	A referral slip given to the mother by the CHW was introduced to counteract the long waiting times at health facilities in this setting. When health workers were sensitised about these referral cards they were more likely to see the mother-baby pair quickly The mix of public and private facilities required a unified approach with both sectors targeted with health facility strengthening interventions

ANC=antenatal care.

### Intervention package design and pilot

The formative research findings were presented at an intervention design workshop in Jinja, eastern Uganda, in September 2008. The primary goal for the intervention design workshop was to apply the gathered evidence to ensure that the designed package, and its components were applicable and appropriate in the context of rural eastern Uganda; that the intervention was consistent with national policies; and that it would be feasible and sustainable. We therefore invited to the workshop the following: representatives from national and regional levels of the Ministry of Health (the key neonatal policy makers and programme coordinators), the two DHTs of Iganga and Mayuge districts where the study was to be done, and non-governmental organisations and United Nations agencies as well as experts in neonatal health, behaviour change communication and those working with community volunteers. Technical assistance for the design workshop was provided by a researcher from the Newhints study in Ghana. Findings from the formative research were presented to invited stakeholders. In addition, the *a priori* UNEST design as per submitted funding protocol, which included piloting the use of community injectable antibiotics and provision of supplies such as thermometers and weighing scales to CHWs, were also presented and discussed. Stakeholders were split into three groups to discuss the community component; health facility strengthening; and the monitoring and evaluation framework. Each group had a facilitator, and they discussed until a consensus was reached. The groups presented conclusions in plenary, and again facilitated discussions were held until consensus was reached.

The CHW package is shown in Table 3. In line with existing Ugandan guidelines in policy but not routinely implemented, CHWs were recruited from the village or parish, trained for 1 week, and supervised by existing health workers from the nearest public sector health unit. In total, 61 CHWs were selected by their communities with the aim of identifying individuals with empathy, experience of similar problems and situations, who were respected in the local community, and considered to be a natural helper or someone that community members would naturally go to in the event of a problem. Female CHWs were preferred although males were also accepted. More details, including the selection, training and supervision of CHWs as well as the content of each visit, can be found elsewhere (13, 24).

**Table 3:** Timing and content of CHW home visit package

Home visits during pregnancy	
1st home visit Timing: First trimester or as early as possible	Negotiation for ANC, at least 4 ANC visits at health facility Birth preparedness; prepare for a health facility delivery with mother and family members Screen for danger signs and facilitate referral Counsel on family planning Health education
2nd home visit Timing: Third trimester, 7–8 months’ gestation	Reinforce birth preparedness Encourage delivery in health facility Counselling on: Maternal and newborn danger signs Family planning Immediate newborn care practices (optimal feeding practices, hygienic cord care, thermal protection)
Postnatal home visits 3rd home visit Timing: Within 24 hours after delivery, or as soon after discharge from the facility as possible	Screen for and counsel on maternal and newborn danger signs, facilitate referral in case of danger signs Counsel on and demonstrate thermal care (skin-to-skin, wrapping and infrequent bathing) Counsel on exclusive breastfeeding including attachment and positioning. If the mother chooses not to breastfeed, the CHW should determine why and support accordingly Counsel on and demonstrate hygienic practices including hand washing and clean cord care Counsel on ITN use, good nutrition, rest for mother and family planning Refer for immunisation (if applicable) and facilitate birth registration
4th home visit Timing: Third day after delivery	Follow-up on issues noted from previous visits Screen for maternal and newborn danger signs and if present facilitate referral Counselling as per previous visit
5th home visit Timing: Seventh day after delivery	Follow-up on issues noted from previous visits Screen for maternal and newborn danger signs and if present facilitate referral Counselling as per previous visit Promote access to under-five clinics and family planning at 6 weeks
Sick and/or small babies Two additional home visits	Follow-up to ensure compliance with referral, or identify reasons why additional care was not sought Provide counselling on extra care for sick and/or small babies, particularly kangaroo mother care

Based on the key messages arising from the formative research and agreed upon at the design workshop, a set of pictorial counselling cards on maternal and newborn health was developed to engage family members. A card with pictorial reminders of the main messages and a space to record the date of the next visit and the CHWs’ contact details was developed, to be left with the family after the first home visit. The family was also provided a template for a birth- preparedness plan for use during counselling. A danger sign screening card was developed to facilitate easier identification of sick newborn babies, as well as a referral card to encourage prompt care-seeking, with space to record the reason for referral in order to assist the health workers, as well as a feedback note to the CHW to be filled in by the health worker who attended to the sick newborn.

CHWs were to be given a set of materials to facilitate their work, and provide credibility and motivation. This included an identity card; a bag for materials; a t-shirt; notebook; counselling and screening cards; referral forms; registers; birth-preparedness forms as well as cards to leave with families. They would also be provided with report forms for monthly reports and a ‘mama kit’ (clean delivery kit) for demonstration to mothers on the key requirements needed for delivery, which the women could purchase at the clinic or through community sellers.

The issue of payment and supervision of CHWs was amongst the biggest decisions made by the UNEST team. In the past, VHTs were not paid by the Government but were dependent on project funds for remuneration, resulting in a non-holistic VHT service. At the same time, non-payment was not deemed acceptable by current and potential CHWs. Through discussions with the national stakeholders working on the VHT strategy as well as international experts engaging in similar work, a decision was taken that all CHWs be paid a token allowance, labelled a transport allowance, to the amount of 10,000 Ugandan shillings (about US$ 5 at the time of the study) monthly, to be given at supervision meetings or refresher trainings. Supervision of CHWs in other projects in Uganda ranged from weekly (mainly in drug distribution trials or projects) to quarterly (as per the Government mandate). It was felt by health workers, CHWs and the study team that less frequent supervision left the CHWs demoralised, but weekly supervision was unsustainable without project-specific staff. Given that the supervisors comprised parish mobilisers, subcounty inspectors and health workers from nearby clinics who had other tasks in their daily remit, a monthly group supervision meeting and directly-observed supervision visits were put in place. As CHWs became more comfortable in their role, supervision was stepped down to take place on a quarterly basis. This strategy has been found to be effective in imparting and retaining skills (25, 26).

The CHW training used materials adapted for Uganda from the regional workshop on Home-Based Care for Mothers and Newborns organised by UNICEF Eastern and Southern African Region in Nairobi, Kenya. The training was conducted by a team of Ugandan health professionals who had attended the training of trainers course, with involvement of DHT staff from the two districts. Many of the trainers were involved in developing training materials and the design workshop. The training for CHWs was skills-based, and focused on promoting key selected practices from formative research and the recommendations of the workshop. While the VHT strategy requires CHWs to be living in the community in which they serve, this carried a potential risk of CHWs not being seen as qualified to give health advice, especially on such a sensitive thematic area as maternal and newborn care – typically the purview of elder women in the family and traditional birth attendants (TBAs). In order to solidify community acceptability, following training all CHWs were to be commissioned as *omuwi w'amagezi*, literally meaning a person who gives health advice.

The health systems strengthening component of the trial was also identified. In addition to home visits by CHWs during pregnancy and after delivery, interventions for demand creation included sensitisation of community leaders and TBAs to the role of the CHWs and the importance of skilled care during childbirth. To address issues of supply, health managers and administrators of private clinics and pharmacies would be engaged and informed of the new home visit package. Health workers in all facilities with a reasonable maternity caseload (15–20 per month) would be given a once-off training in newborn care. In addition, it was decided that basic equipment and supplies (e.g. partographs, weighing scale, neonatal Ambu bag and masks for resuscitation) and a catalytic, once-off supply of drugs for newborn care were to be provided to health facilities. Full details of the intervention are described elsewhere (13, 24).

Following the intervention design workshop, meetings to adapt the UNICEF materials were held with support from the Ministry of Health. Other materials of reference came from the Ministry of Health, WHO, Ghana and Malawi; these were scrutinised, analysed, modified and adapted to the local rural Ugandan context. Guided by a principle of designing randomised control trials, a decision was taken that the pilot study would be conducted in Ibulanku subcounty outside the HDSS in order to avoid later-stage contamination. Based on results of the pilot, the package was further refined.

### Implementation of the package, stakeholder engagement and national uptake

Following the pilot, CHWs were recruited and trained within the HDSS. They started home visits with only counselling cards and forms. Later the CHW bags, inclusive of the demonstration mama kit, were obtained and distributed to each CHW. The chairmen of each village in which CHWs resided were invited to the deployment meeting. Results of in-depth interviews with local council leaders, facility-based health workers and families identified that CHWs were highly appreciated in the community and seen as important contributors to maternal and newborn health at grassroots level (27). CHWs themselves reported being highly motivated to do their work, arising out of the fact that they were selected by and trained in the community and provided a service which linked to the formal health system. Intrinsic motivators (e.g. community appreciation and the prestige of being ‘a doctor’), monetary (such as a small transport allowance) and material incentives (e.g. bicycles, bags) were important motivators to varying degrees.

There was engagement of policymakers and decision leaders from village to national level. Several forms of engaging were used, including meetings, brochures, newspaper publications, television interviews, documentary videos, scientific publications, presentations at conferences, and field visits. These efforts were based on resources and relationships already existing within the study team, but new relationships were made as necessary to ensure that key decision-makers were informed. Reports and materials were regularly shared with key stakeholders. At the district level, an implementation committee consisting of representatives from the two DHTs and research team met regularly throughout the design and pilot phase to make operational plans about community entry, sensitisation and selection of CHWs, training, supervision, the motivation system for CHWs and other intervention activities. The committee also looked at the proposed workloads for CHWs to assess how feasible they were and how they compared with volunteer CHWs in other settings with similar training. Using a standardised tool developed specifically to assess financial and human costs of newborn care services, time spent by the CHW on home visits, activities related to the programme, and other health-promotion activities was assessed through a self-reported survey of 18 randomly selected CHWs (29% of total CHWs in the intervention villages). Similarly, seven supervisors (38% of total supervisors in the intervention villages) were randomly selected and asked to record time spent on supervision. The relatively low average time spent per home visit (82 min) and number of visits per week (1.5) were used as advocacy messages to demonstrate to DHTs and national policymakers that home visits are a feasible addition and do not constitute a massive time commitment on the part of the volunteers.

The multidisciplinary advisory body to the Ministry of Health on newborn health issues, the National Newborn Steering Committee (NNSC), established in 2006, provided an obvious platform for sharing information, receiving critical feedback and learning about similar efforts under way in other areas of the country. The NNSC undertook a field visit to UNEST to learn more about the home visit package as well as to provide technical input to project staff and the implementation committee. The implementation committee meetings as well as NNSC discussions were used as an opportunity to gain consensus on key decisions and to vet products as well as identify next steps in the design process. Workshops involving external technical experts further aided this process.

At the start of the UNEST implementation the Ministry of Health was also developing guidelines, materials and policies for revitalisation of the national VHT strategy, which called for 5–6 CHWs in each village with 1–2 devoted to maternal, newborn and child health activities. Additionally, the national adaptation of integrated community care management (ICCM) of childhood illness was ongoing, but was only capturing services for children aged 2–59 months. The UNEST team took advantage of this opportunity to share experiences and materials. As a result, the national VHT and ICCM packages include the home visit schedule, key messages around newborn care, and UNEST behaviour change communication materials. These packages are being scaled-up for nationwide use at community level. The facility-based training materials on newborn care have also been used in trainings around the country and incorporated into the national service standards for newborn care (28). Finally, a documentary video highlighting newborn care within UNEST was adopted by the Ministry of Health to be used as an advocacy tool at meetings and on national television.

## Discussion

UNEST achieved significant improvements in birth preparedness and the essential newborn care practices, including breastfeeding, hygienic cord care and thermal protection – practices that are associated with reduced neonatal mortality. The general improvement in these and other practices and service coverage across both the intervention and control areas may be explained at least in part by the health facility strengthening which impacted both trial arms, but also by the secular trend towards improved maternal and newborn care. CHWs who were selected by their communities with district-led training and supervision were able to identify and visit almost all the pregnant women, especially those from the poorest families and those who delivered at home or with TBAs.

This analysis shows how evidence was used to adapt new interventions proven elsewhere into the Uganda health system policy and programme context, and how this led not only to an adapted intervention but also to rapid policy adaptation and scale-up of components of UNEST while the study was still going on. Key success factors included identifying high-impact best practices, rigorous formative research and pilot testing to develop a package that fits the local context, wide stakeholder engagement and involvement, and taking advantage of a policy window with consistent messages. These findings may have implications for how interventions found to be effective in other contexts can be adapted into local policies and programmes. However, caution is required so as not to oversell the effectiveness of a package prior to availability of local evidence.

Evidence from outside of Uganda, particularly the Asian community-based newborn care trials, were extremely influential in increasing attention for the overlooked burden of newborn mortality in the country. Policy guidelines and training materials from UNICEF, the WHO and others on newborn care were also widely disseminated. The third paper in *The Lancet* Neonatal Series – which greatly influenced UNEST and newborn care in Uganda overall – presents a framework for systematically scaling-up newborn care that has been followed closely (Panel 1 ) (29). While other countries focused primarily on strengthening community level platforms (30), the decision to systematically address community and facility issues in UNEST has set the stage for newborn health programming nationwide. The decision for CHWs to undertake a preventive/promotive health package and not treat neonatal sepsis makes sense in this context, but perhaps limits the scope of impact of these workers. Identifying newborns with danger signs and ensuring prompt care-seeking was challenged by the routine visit schedule (i.e. passive case-finding). Compliance with referral for sick newborns was thought to be high, but also associated with the perceived quality of care at the nearest health facility and outside the control of the CHW (27). This is also an issue where CHWs provide curative services; preventive and promotive messages given through routine counselling visits may not receive as high priority (31).

Panel 1 **Steps to scale-up neonatal health**

Step 1: Assess the situation and create a policy environment conducive to neonatal health

Step 2: Achieve optimum neonatal care within the constraints of the situation

- Start with outreach or family-community care if the health system is not strong
- Identify and address missed opportunities within the formal healthcare system
- Coordinate across programmes relevant to neonatal health

Step 3: Systematically scale-up neonatal care

- Strengthen supply
- Improve demand
- Overcome supply and demand obstacles

Step 4: Monitor coverage and measure effect and cost

The formative research together with the quantitative baseline data collection identified key knowledge gaps and informed an iterative process to design a package of interventions specifically tailored to the context at the time. Experienced researchers, including external technical assistance were required for this component building on similar formative research in Ghana (9) as well as Nepal (32). Triangulation of results with findings from other assessments in Uganda identified some of the differences in the local settings and would be advisable, although not essential, upon introduction in other districts.

The decision to engage a wide group of stakeholders from the outset allowed for increased ownership and the ability to elicit expert experience. These stakeholders participated in designing the intervention, behaviour change materials and health worker training materials. The engagement of the NNSC was particularly strategic given their prominent role in newborn health policies and programming (33). In the absence of such a body, substantially more effort would have been required at national level to rally interested or potentially interested partners and to join up other efforts working on the same or similar goals through health systems strengthening and/or VHTs, for example, ICCM of childhood illness. One challenge encountered with district-level stakeholders outside the study area was the concern that the intervention might not be replicable given the study site within the HDSS. At the local level involvement of the DHTs and health facility staff in monitoring and supervision is likely to have increased sustainability of the intervention, and having DHTs involved in dissemination and speaking in various fora provided a sense that the package was not wholly led by researchers but owned by the whole community. The intervention has since been taken up in six nearby districts.

The success of UNEST in rapidly influencing policy could be attributed firstly to the stakeholders’ active engagement, but also to the existing policy window for home-based preventive and promotive newborn care. At the time there was demand from national-level stakeholders and external pressure to develop effective low-cost interventions to reduce neonatal mortality in order to accelerate efforts towards achieving Millennium Development Goal 4. Thus, in designing UNEST there was an *a priori* goal of designing an intervention that would inform policy and lead to rapid scale-up. The remit was expanded somewhat following the baseline findings, so that it also allowed for health systems strengthening and addressing low quality of facility-based care. However, major decisions were taken which limited the intensity of the intervention in order to keep it in line with national policy guidelines. Both CHW and health worker trainings were no more than a week in duration, and CHW supervision was by health workers from nearby health facilities rather than UNEST project staff. Also in line with existing guidelines, the CHWs were not paid and instead were given transport refunds and other small tokens as incentives.

Despite the rigorous design and pilot phase, ongoing process monitoring and, to a certain degree, adaptation is also important. For instance, CHWs preferred doing pregnancy home visits as opposed to postnatal visits in the first few days after birth. When supervisors were provided this information, they were able to adjust guidance, leading to sustained improvements in both pregnancy and postnatal home visits. Another challenge was that families and CHWs were not adequately identifying small babies, given that CHWs were not given a weighing scale because of the prohibitive cost. A separate study was conducted to design and validate a foot length card that CHWs carry with them and use during the first postnatal visit. The foot length card has been taken up for use within the national VHT roll-out (34). Where these package adaptations occur in a study setting, particularly a randomised design, this makes the intervention an evolving one, which needs to be taken into account in trial design and reporting.

The uptake of elements of the UNEST package prior to completion of the trial reflects the success of this approach, but also the favourable policy window for both newborn care at both facility and community levels. Researchers should be careful not to oversell the intervention and to identify which elements may be specific to evaluation of the package and which are necessary for expansion. Additionally, there should be scope to re-evaluate the package when results are available. The cost of scaling-up the package to both the health system and families is also important to consider.

## Conclusions

Interventions proven elsewhere and recommended by international experts require local adaptation if they are to fit into local health systems, be accepted by the community and taken to national scale. Understanding local epidemiology, sociocultural context and barriers to seeking care can play an important role in designing an integrated package of health system improvements. The use of evidence combined with local testing and engagement of key stakeholders at community, district and national level was critical to adapting and testing the UNEST intervention package that has been taken up widely and integrated into broader community-based and facility-level packages of care.

## References

[1] Darmstadt GL, Walker N, Lawn JE, Bhutta ZA, Haws RA, Cousens S (2008). Saving newborn lives in Asia and Africa: cost and impact of phased scale-up of interventions within the continuum of care. Health Policy Plan.

[2] Bang AT, Bang RA, Reddy HM (2005). Home-based neonatal care: summary and applications of the field trial in rural Gadchiroli, India (1993 to 2003). J Perinatol.

[3] Manandhar DS, Osrin D, Shrestha BP, Mesko N, Morrison J, Tumbahangphe KM (2004). Effect of a participatory intervention with women's groups on birth outcomes in Nepal: cluster-randomised controlled trial. Lancet.

[4] Baqui AH, El-Arifeen S, Darmstadt GL, Ahmed S, Williams EK, Seraji HR (2008). Effect of community-based newborn-care intervention package implemented through two service-delivery strategies in Sylhet district, Bangladesh: a cluster-randomised controlled trial. Lancet.

[5] Kumar V, Mohanty S, Kumar A, Misra RP, Santosham M, Awasthi S (2008). Effect of community-based behaviour change management on neonatal mortality in Shivgarh, Uttar Pradesh, India: a cluster-randomised controlled trial. Lancet.

[6] Bhutta ZA, Soofi S, Cousens S, Mohammad S, Memon ZA, Ali I (2011). Improvement of perinatal and newborn care in rural Pakistan through community-based strategies: a cluster-randomised effectiveness trial. Lancet.

[7] WHO, UNICEF, USAID, Children, St. WHO-UNICEF Joint Statement (2009). Home visits for the newborn child: a strategy to improve survival.

[8] Countdown to 2015 for Maternal, NaCS (2013). Accountability for maternal, newborn and child survival.

[9] Hill Z, Manu A, Tawiah-Agyemang C, Gyan T, Turner K, Weobong B (2008). How did formative research inform the development of a home-based neonatal care intervention in rural Ghana?. J Perinatol.

[10] Kirkwood BR, Manu A, ten Asbroek AH, Soremekun S, Weobong B, Gyan T (2013). Effect of the newhints home-visits intervention on neonatal mortality rate and care practices in Ghana: a cluster randomised controlled trial. Lancet.

[11] Kilbourne AM, Neumann MS, Pincus HA, Bauer MS, Stall R (2007). Implementing evidence-based interventions in health care: application of the replicating effective programs framework. Implement Sci.

[12] Bahl R, Martines JC, Kirkwood BR, Pelto P (2008). Introduction: using research for improved delivery of newborn health interventions. J Perinatol.

[13] Waiswa P, Pariyo G, Kallander K, Akuze J, Namazzi G, Ekirapa-Kiracho E (2014). Effect of the Uganda Newborn Study on care-seeking and care practices: a cluster-randomised controlled trial. Glob Health Action.

[14] WHO (2011). Beginning with the end in mind: planning pilot projects and other programmatic research for successful scaling up.

[15] Darmstadt GL, Bhutta ZA, Cousens S, Adam T, Walker N, de Bernis L (2005). Evidence-based, cost-effective interventions: how many newborn babies can we save?. Lancet.

[16] Lawn JE, Cousens S, Zupan J (2005). 4 million neonatal deaths: when? Where? Why?. Lancet.

[17] Lawn J, Kerber K (2006). Opportunities for Africa's newborns: practical data, policy and programmatic support for newborn care in Africa.

[18] Edmond K, Bahl R, Tawiah-Agyemang C, Lawn J, Kirkwood B (2006). Breast is still best: new evidence of the potential to save at least 1.4 million lives. The Commonwealth Health Ministers Reference Book.

[19] Tawiah-Agyemang C, Kirkwood BR, Edmond K, Bazzano A, Hill Z (2008). Early initiation of breast-feeding in Ghana: barriers and facilitators. J Perinatol.

[20] Waiswa P, Kallander K, Peterson S, Tomson G, Pariyo GW (2010). Using the three delays model to understand why newborn babies die in Eastern Uganda. Trop Med Int Health.

[21] Waiswa P, Nyanzi S, Namusoko-Kalungi S, Peterson S, Tomson G, Pariyo GW (2010). I never thought that this baby would survive; I thought that it would die any time’: perceptions and care for preterm babies in Eastern Uganda. Trop Med Int Health.

[22] Waiswa P, Peterson S, Tomson G, Pariyo GW (2010). Poor newborn care practices – a population based survey in Eastern Uganda. BMC Pregnancy Childbirth.

[23] Waiswa P, Kemigisa M, Kiguli J, Naikoba S, Pariyo GW, Peterson S (2008). Acceptability of evidence-based neonatal care practices in rural Uganda – implications for programming. BMC Pregnancy Childbirth.

[24] Waiswa P, Peterson SS, Namazzi G, Ekirapa EK, Naikoba S, Byaruhanga R (2012). The Uganda Newborn Study (UNEST): an effectiveness study on improving newborn health and survival in rural Uganda through a community-based intervention linked to health facilities – study protocol for a cluster randomized controlled trial. Trials.

[25] Nalwadda CK, Waiswa P, Kiguli J, Namazzi G, Namutamba S, Tomson G (2013). High compliance with newborn community-to-facility referral in Eastern Uganda: an opportunity to improve newborn survival. PLoS One.

[26] Kayemba Nalwadda C, Guwatudde D, Waiswa P, Kiguli J, Namazzi G, Namutumba S (2013). Community health workers – a resource for identification and referral of sick newborns in rural Uganda. Trop Med Int Health.

[27] Okuga M, Kemigisa M, Namutamba S, Namazzi G, Waiswa P (2014). Engaging community health workers in maternal and newborn care in eastern Uganda. Glob Health Action.

[28] Ministry of Health (2010). Newborn implementation framework: standards for newborn health care services.

[29] Knippenberg R, Lawn JE, Darmstadt GL, Begkoyian G, Fogstad H, Walelign N (2005). Systematic scaling up of neonatal care in countries. Lancet.

[30] Lawn JE, Kinney MV, Black RE, Pitt C, Cousens S, Kerber K (2012). Newborn survival: a multi-country analysis of a decade of change. Health Policy Plan.

[31] Nalwadda Kayemba C, Naamala Sengendo H, Ssekitooleko J, Kerber K, Kallander K, Waiswa P (2012). Introduction of newborn care within integrated community case management in Uganda. Am J Trop Med Hyg.

[32] Morrison J, Osrin D, Shrestha B, Tumbahangphe KM, Tamang S, Shrestha D (2008). How did formative research inform the development of a women's group intervention in rural Nepal?. J Perinatol.

[33] Mbonye AK, Sentongo M, Mukasa GK, Byaruhanga R, Sentumbwe-Mugisa O, Waiswa P (2012). Newborn survival in Uganda: a decade of change and future implications. Health Policy Plan.

[34] Nabiwemba E, Marchant T, Namazzi G, Kadobera D, Waiswa P (2013). Identifying high-risk babies born in the community using foot length measurement at birth in Uganda. Child Care Health Dev.

# Sub article: Engaging community health workers in maternal and newborn care in eastern Uganda

**DOI:** 10.3402/gha.v8.23968

**Published:** 2015-03-31

**Authors:** Monica Okuga, Margaret Kemigisa, Sarah Namutamba, Gertrude Namazzi, Peter Waiswa

**Affiliations:** 1 Department of Health Policy, Planning and Management, School of Public Health, Makerere University, Kampala, Uganda; 2 Private Consultant, Kampala, Uganda; 3 Global Health, Department of Public Health Sciences, Karolinska Institutet, Stockholm, Sweden; 4 Iganga-Mayuge Health Demographic Surveillance Site, Iganga-Mayuge, Uganda

**Keywords:** newborn health, maternal health, community health worker, pregnancy, postnatal care, Uganda

## Abstract

**Background:**

Community health workers (CHWs) have been employed in a number of low- and middle-income countries as part of primary health care strategies, but the packages vary across and even within countries. The experiences and motivations of a multipurpose CHW in providing maternal and newborn health have not been well described.

**Objective:**

This study examined the perceptions of community members and experiences of CHWs around promoting maternal and newborn care practices, and the self-identified factors that influence the performance of CHWs so as to inform future study design and programme implementation.

**Design:**

Data were collected using in-depth interviews with six local council leaders, ten health workers/CHW supervisors, and eight mothers. We conducted four focus group discussions with CHWs. Respondents included 14 urban and 18 rural CHWs. Key themes explored included the experience of CHWs according to their various roles, and the facilitators and barriers they encounter in their work particular to provision of maternal and newborn care. Qualitative data were analysed using manifest content analysis methods.

**Results:**

CHWs were highly appreciated in the community and seen as important contributors to maternal and newborn health at grassroots level. Factors that positively influence CHWs included being selected by and trained in the community; being trained in problem-solving skills; being deployed immediately after training with participation of local leaders; frequent supervision; and having a strengthened and responsive supply of services to which families can be referred. CHWs made use of social networks to identify pregnant and newly delivered women, and were able to target men and the wider family during health education activities. Intrinsic motivators (e.g. community appreciation and the prestige of being ‘a doctor’), monetary (such as a small transport allowance), and material incentives (e.g. bicycles, bags) were also important to varying degrees.

**Conclusions:**

There is a continued role for CHWs in improving maternal and newborn care and linking families with health services. However, the process for building CHW programmes needs to be adapted to the local setting, including the process of training, deployment, supervision, and motivation within the context of a responsive and available health system.

Globally 2.9 million babies die during their first month of life (the newborn period), and an additional 2.6 million babies are stillborn each year. About three-quarters of newborn deaths occur within the first week of life, with 25–45% occurring within the first 24 hours of life (1, 2). Technical agreement has advanced around what to do to improve health and survival for mothers and their babies in the poorest countries, but how to achieve these improvements remains a challenge. One approach has been the reinvigoration of comprehensive primary health care, including renewed recognition of the importance of community ownership and expanded use of health extension and community health workers (CHWs) (3).

The need for community-level access to care is highest in low-income countries. In Uganda, nearly all women (94%) access antenatal care (ANC) at least once, but deliveries at a health facility, while increasing, remain much lower (57%) (4). Only 59% of births are attended by a skilled health provider, with lower coverage in rural areas (4). One study, which looked at the location of neonatal deaths in eastern Uganda, revealed that 54% of newborns died outside of a health facility (5).

The feasibility of a community-based strategy in effectively reducing neonatal mortality within settings of weak health systems, low service utilisation, and high neonatal mortality has been demonstrated in trials and demonstration projects in Asia (6–9). In trials in Asia, home visits by trained CHWs to promote preventive care as well as to provide curative care reduced neonatal mortality by about 30% (10). However, there are little data on experiences of integrating CHWs into health systems to improve maternal and newborn outcomes in settings in sub-Saharan Africa.

Knowledge gaps exist in defining how to sustainably implement and scale-up community-based interventions for maternal, newborn, and child health, and how these interventions not only improve home care practices but also create demand for healthcare services in the broader health system. The Uganda Newborn Study (UNEST) was among the first community-based trials in sub-Saharan Africa to test a package of home visits during pregnancy and the postnatal period using CHWs linked to health system strengthening for maternal and newborn care (11, 12).

This paper is the third in a series on the impact and findings of UNEST. This qualitative study sought to document experiences within UNEST of CHWs in promoting healthy home behaviours and demand for maternal and newborn care services, to identify community perceptions of this CHW role, and to identify and explore the factors that influence the performance of CHWs in both urban and rural settings in order to inform future programming.

## Methods

### Setting

This study was carried out in the Iganga/Mayuge Health Demographic Surveillance Site (HDSS), located in Iganga and Mayuge districts in south-eastern Uganda. The HDSS covers an area of 155 km^2^ and has a population of about 70,000 people, at the time of the study, in 65 villages, 18 parishes, and 12,000 households. It is a largely rural area, with the main economic activity being subsistence farming. Within the HDSS, there is one government hospital, 15 health centres, 24 private clinics, and other informal health providers, mainly traditional birth attendants and drug shops. These informal health providers are mainly found in small trading centres.

### Intervention

The UNEST package was developed through formative research which included a literature review, consultation with technical experts, policy makers, local leaders, and community-based data collection to inform the intervention design and development of counselling and behaviour change materials, the details of which have been described elsewhere (12). Sustainability and scale-up feasibility were considerations from the outset of the study design phase. The intervention delivered in the cluster randomised control trial comprised a community-based home visit package which linked to health facility services. CHWs were recruited from the community (Box 1), based on criteria stipulated in the national Village Health Team guidelines: a regular/permanent resident of the community, literate and willing to work as a volunteer (13). A preference was given to mature females already doing some community health work. They were trained to identify pregnant women and make two pregnancy visits and three postnatal visits in the first week after birth, providing health education on pregnancy, preparation for childbirth and newborn care (Box 2). More details of this study can be found in the published UNEST protocol paper (12).

Box 1 **CHW selection, training and deployment**

**Selection process**

- CHW post advertised according to criteria set using Ministry of Health Village Health Team criteria
- Community identifies a selection team
- Candidates interviewed and CHW post filled

**CHW training**

- Five-day training on preventive and promotive maternal and newborn care and counselling skills
- Teaching methods include participatory discussion, practical demonstrations and role-playing

**CHW deployment and supervision**

- CHWs were given a set of materials including a register, picture-based counselling cards on birth preparedness and maternal and newborn care, an example of the ‘mama kit’ (delivery kit) and monthly reporting forms
- For easy identification they were given branded t-shirts and identity cards
- Directly observed supervision visits and group supervision meetings took place monthly until competency was reached, and quarterly thereafter
- Supervisors were nurses/midwives from the local health unit
- ‘Super’ CHWs nominated as leaders were tasked with mobilising and encouraging fellow CHWs and serving as supervisors where health workers were not available
- CHWs were instructed to identify pregnant women in their catchment area, make two home visits during pregnancy and three home visits in the first week after delivery with extra visits for women or newborns with complications

Box 2 **Content of care during CHW home visits**

***Home visits during pregnancy***

*First pregnancy visit (Target: as early as possible)*

- Counsel on and refer for ANC including tetanus immunisation and malaria prevention
- Counsel on birth preparedness and clean delivery practices
- Counsel on and assess danger signs of pregnancy, refer if present
- Counsel on and refer for HIV testing

*Second pregnancy visit (Target: in third trimester)*

- Counsel on birth preparedness and clean delivery practices
- Counsel on and assess danger signs of pregnancy, refer if present
- Counsel on newborn care practices immediately following delivery (e.g. placing the baby skin-to-skin, ensuring warmth, initiating breastfeeding, hygienic cord care)
- Counsel on newborn danger signs

***Home visits in the postnatal period***

*First postnatal visit (Target: birth day to day 3)*

- Counsel on and assess maternal and newborn danger signs, refer if present
- Promote thermal care (skin-to-skin placement, delayed bathing, and wrapping)
- Support for exclusive breastfeeding
- Encourage cleanliness especially cord care

*Second postnatal visit (Target: day 5–7 after birth)*

- Counsel on and assess maternal and newborn danger signs, refer if present
- Refer for immunisation
- Counsel mother on breastfeeding and postnatal family planning
- Reinforce need to seek care or call CHW for support

*Extra care for sick and/or very small newborns*

- Follow-up visit post-referral, or two extra home visits if referral is not accepted
- Extra counselling on thermal care
- Extra support for breastfeeding
- Extra attention to hygiene

### Data collection

Data were collected through in-depth interviews (IDIs) and focus group discussions (FGDs). Participants included facility-based health workers; members of the District Health Team and local council (village leaders); recipients of CHW services (mothers with children less than 6 months of age); and CHWs selected from both urban and rural areas (Table 1). Interview guides and data collection tools were pretested for each group of respondents and standardised. Key themes explored included the perceptions of CHWs amongst community members, and the experiences of CHWs in carrying out their various roles as well as the facilitators, barriers, and achievements they encountered through their work.

**Table 1:** Overview of study participants and key themes explored

Type of participant	Role in the intervention	Number interviewed	Data collection method	Focus of data collection
District Health Management Team	Selection of CHWs, participate in training and group supervision	2	IDI	Experiences interacting with CHWs Perceptions of technical competency, ability to make linkages and support, continuity of services; effectiveness of CWHs’ work; sustainability, barriers and suggestions to overcome barriers to CHW approach Community behaviours around pregnancy and newborn care
Facility-based health workers (nurses and midwives)	CHW supervisors	6	IDI
Local community leaders (urban and rural)	Sensitisation of communities to CHWs	6	IDI
Community health workers (CHWs)	Home visitors		IDI, FGD	Roles, facilitating factors, achievements, challenges, motivations, expectations, changes associated with their work, experience with health workers
Urban		14	
Rural		18	
Mothers of children under 6 months	Clients of CHWs		IDI	Experiences interacting with CHWs Experience of danger signs and response
Seen at least once by CHW		4	
Seen by CHW and accepted referral		4		

### Data analysis

Interview data were transcribed and analysed using manifest content analysis (14). Field notes, contact summary sheets and transcripts of the tape-recorded interviews and FGDs were read to identify key themes. Pattern coding was done by conducting close and repeated readings of the field notes and contact summary sheets in order to discover patterns within the emerging themes, so as to deduce smaller analytical units to further elaborate the themes. Comparison was made of data from the different participants to explore commonalities of experiences and observations in view of the emerging themes. Results were presented thematically.

Ethical clearance was obtained from Makerere University School of Public Health Higher Degrees Research and Ethics Committee and the Uganda National Council of Science and Technology. In addition, approval was sought from the district authorities and local leaders in the communities where the study is being conducted. Informed verbal and written consent was sought from all study respondents.

## Results

### CHW selection, training, deployment and supervision

While women were the preferred candidates for the CHW role, in practice a number of communities selected men as CHWs. Participants reported that indeed most of the active CHWs and all the CHW leaders (‘super CHWs’) selected by the CHWs themselves were men. CHWs were trained near their homes in a mix of English and the local language, mainly using what was described as a ‘hands on’ approach. The CHWs were also deployed immediately after training and were introduced to the community by the local leaders.

Given the short (7-day) training and immediate deployment, a number of CHW participants reported that they felt ‘not ready’ and needed more supervision than they received. The main challenges to CHW confidence reported by health providers and other community stakeholders were perceived low levels of education and social status of the CHWs in the community, which affected their acceptability at the start of the intervention. At the outset, CHWs were seen as ‘just local people’ who are ‘ordinary’, without any medical training. Said one rural female CHW: ‘Local people used to say we are just from the same village, what do we have to talk to them about?’

Discussions with health workers, community leaders, and CHWs all pointed to the social status of CHWs as a source of tension in the delivery of their services. The CHWs lacked confidence in approaching households of people they considered to be of ‘higher social status’, such as those who were wealthier or more educated, including the families of health workers, sometimes leading to limited or no interaction at all:

When you look at the scenario, it is a bit complicated … it is just like a family talk or a friendly talk as CHWs disseminate information, and the barriers could be the condition they are working in may not be favourable; some families may seem higher than the volunteer, so they may look at the information they are giving as not worth it as regards to the social status or as regards to education status. The poor young man or women feels threatened; yet the project targets the whole community. So, that is a major barrier. (Member, District Health Team)

However, CHWs reported that given time this barrier was overcome with supervision and increased confidence. In UNEST, support supervision was provided first through monthly directly observed supervision visits with each CHW separately and monthly group meetings. The monthly group supervision was later shifted to a quarterly basis once CHWs were considered to be performing adequately in their role. Community members gradually gained trust in the CHW work and began to open up and seek CHWs’ assistance:

As I told you, at first the community members used not to value the CHWs; these days it is the community that explains to those who are hesitant to get the service from us, our importance as CHWs has improved and people who have sick children also come to seek advice from us, which was not the case before. (Male CHW, urban)

At first the people were [*looking down on*] us since we were staying in the same community … they say ‘this person who trained for one week’, but now they appreciate. You would reach a home and they ignore you and they tell you to leave the place, and they tell you as they don't have time to attend to you, but these days they have changed. I think they learnt the importance slowly by slowly. And those we have given referrals so far have appreciated our work and they have done a very big job of sensitising the rest in the communities. Because at first I had a lady whom I used to visit … and she told me she never wanted me to visit her. But later during the course of her pregnancy she got complications with the pregnancy and her legs got swollen … I referred her to Nakavule and they treated her. Since then she knows the importance of CHWs and whenever she could get complications with her body she could come and seek advice from me. (Male CHW, urban)

### Changing beliefs and practices and around pregnancy and newborn care

Pregnancy, childbirth and the newborn period is surrounded by many cultural beliefs and traditional practices that could serve as a barrier to CHW work. Despite this challenge, CHWs were perceived as successful in changing certain practices during pregnancy and for newborn care (Table 2). CHWs revealed that initially it was very difficult for them to identify pregnant women easily, partly due to the local Kisoga culture which dictates that pregnancy be kept a secret. Both health workers and CHWs reported that more women were attending ANC earlier in their pregnancies, with the exception of younger women and older women who were more likely to keep the pregnancy hidden for longer.

**Table 2:** Changes attributed to CHWs’ activities

Change described	Examples given of CHW-enforced behaviours associated with the change
Changes during pregnancy and labour	
Increased ANC attendance	Pregnant women and husbands/partners informing CHWs of a pregnancy; more women attending ANC during the first trimester
Increase in birth- preparedness activities	Husbands/partners save money, provide women with money for emergencies, transport, and babies’ needs
	Women buy mama kit items
	Women ask CHWs to educate them in presence of husbands
Increased knowledge on pregnancy health issues	Women attend to their health needs during pregnancy
	Women try to eat healthy/balanced diets
More likely to seek care	Women recognise danger signs
	Women initiate contact with CHW or health facility in case of swelling, general weakness, or if a decrease in baby's movements felt
	More deliveries at health facilities
Improved health worker/client relationship	Women experience a caring attitude from health workers, as the latter are less constrained when women have mama kits and ANC cards
Health workers recognise referrals	Women with CHW referral slips are seen faster at hospital or health unit
Changes during postnatal period	
Increased awareness of needs of newly delivered mothers and babies	Women put only salty water on the baby's umbilical cord rather than animal dung and herbs
	Bathing is delayed instead of immediately practised
	Men also acknowledge the importance of these practices
	CHWs are sought outside of routine visits regarding danger signs
More use of health facilities	More women taking their newborn babies for postnatal care, including immunisation
	Seeking medical care from qualified persons
	Newly delivered mothers reaching out to CHWs when self or infant is unwell
Improved breastfeeding	Immediate breastfeeding at birth and continuous breastfeeding
	Delayed introduction of other feeds
	More women giving colostrum

CHWs capitalised on social networks to identify pregnant women who would become new clients, learn about births and disseminate information. Husbands were reported to approach male CHWs in particular when their wives fell pregnant. Some women reported getting to know the CHWs through social ties including extended family members and friends. It was further reported that even when fetching water or during social gatherings, community members would inform CHWs about a women with signs of pregnancy, or to report that a woman was in labour. Participants associated several changes to birth preparations in response to the CHW intervention. In particular, the role of men in supporting their expectant wives was mentioned. When the husband was part of counselling during pregnancy, decision making around saving money and seeking care was perceived to be easier:

There is a very big change because now women buy things to use in a health facility, and when you go back to them for the second visit and ask these women to show you the things she can show you, the things you told her to buy. (Male CHW, urban)

I think maternal mortality has reduced because as you know if a mother comes and she doesn't have anything, at times the health workers may not take any interest to handle her, but if she has everything then the care is also given, because she has everything she needs to examine her – she has the gloves, she has everything. The health workers definitely doesn't have a second thought of where to get the gloves or now where will I get the polythene sheet? He or she will examine the woman and attend to her. (Health worker)

Men generally used not to take it serious but now they prepare, and because we teach them before delivery they save or work hard so that they can buy what is necessary during delivery. (Female CHW, rural)

CHWs revealed that it was helpful to start introducing newborn care concepts during pregnancy visits rather than waiting until the baby had arrived. Mothers noted that they learned things about caring for the baby that they didn't know previously, including newborn danger signs and the urgency around seeking care for them. Breastfeeding messages were well received, and support for attachment and positioning was appreciated by mothers. CHWs and health workers reported that practices were more likely to be sustained if supported culturally, regardless of what healthcare staff were promoting.

Participants noted that while communities appreciate the education received, some of the suggested behaviours have been slow to take root. In particular, practices around cord care were difficult to change completely. Women in this study reported that previously they used powder, ash and dung on the baby's umbilical cord in order to make it heal faster. While practices shifted away from placing these types of substances on the cord, the message to place nothing on the cord was not prioritised by CHWs, and even when delivered it was not well received by mothers. Even health workers promoted cleaning the cord with plain water: ‘What has changed is the fact that before, people would apply all sorts of things to the cord, but right now we are told not to apply anything on the cord apart from cleaning it with water’ (Mother, rural).

Changes in behaviour around thermal care were also associated with CHW visits. Women, especially first-time mothers, reported that they were taught how to wrap babies for warmth and noted the importance of delaying bathing and not keeping the babies in wet bedding. However, not all of the targeted practices were equally accepted: skin-to-skin care was not frequently mentioned as a thermal care practice, but rather CHWs and mothers revealed that advice was given to buy cotton clothes, baby hats, and socks to keep the baby warm. However, the few CHWs and mothers who had promoted and practised skin-to-skin care for low-birthweight babies were animated proponents of the practice, having witnessed its dramatic results in babies previously thought to be beyond assistance.

### Referral and linking with health facilities

CHWs revealed that during each routine visit they screen women and newborns for danger signs and refer if needed, sending the mother to a health facility with a referral note so that the health workers see them quickly. Overall health workers considered CHWs to be technically strong in providing knowledge and skills to the families and providing services in accordance with client expectations. Follow-up and feedback were regarded by mothers who had been referred as very important in creating accountability for referral compliance and instilling confidence in the service. Participants including District Health Team members noted that when a health facility is poorly staffed and equipped, there is less motivation for women to comply with referral:

I sent there a mother for delivery, but reaching [the health unit] it was around 5 pm; she couldn't receive the services … She had to go to another health facility and she later came up to [the district hospital]. (Female CHW, rural)

So, the CHW sends a mother and she doesn't receive the services and she comes back; the next time you try to send her to the health facility, it creates a barrier. (Member, District Health Team)

### Barriers and challenges experienced by CHWs

CHWs were faced with a number of challenges that influenced their performance (Table 3). At the operational level CHWs were challenged by a lack of transport, particularly for those with larger areas to cover, which affected their ability to reach their clients at targeted times and in the case of complications. They also revealed that when they propose the use of the ‘mama kit’, families always ask where they could buy the recommended materials because they are not always available. UNEST was implemented in an area where communities are exposed to other health-related research activities. These interventions, which also use community members as volunteers, have different implementation frameworks and support mechanisms for volunteers and ways of engaging communities. As a result, CHWs were faced with demands for material support from families which had received such support from previous studies.

**Table 3:** Overview of challenges faced by CHWs and proposed solutions

Challenge raised	Implications for the intervention	Proposed solution
Transport	Lack of transport, translating into delays in reaching their clients, or not reaching them at all	Request for provision of transport or transport allowances
Mama kits not available	Some pregnant women expect CHWs to provide mama kits	Ensure mama kits are available and in designated clinics and drug shops and subsidised where possible
Confusion about CHW services Research fatigue	An earlier intervention study included the provision of soap and other things to mothers. This created expectations of the same from subsequent home-based interventions.	Encourage harmonised approaches to community activities and for all activities to follow District Health Team protocol
Research fatigue	Given the high level of studies in the intervention area, some women ignored CHWs, citing overuse	HDSS sites need to ensure that they exclude households from multiple or back-to-back studies
Apprehension from some local leaders	Some local leaders felt threatened by the recruitment of volunteers not directly under their supervision	Build trust and establish good rapport with key influentials
Lack of CHW confidence	Few women sought CHW services early on; delayed appreciation for CHW services	Engage local leaders early on in mobilisation and mediation roles as well as community sensitisation Use community structures to clarify roles Develop strong supervision from the outset Provide refresher training and additional opportunities for CHWs to gain skills
Competing demands on CHW time	Target time for home visits not met; clients with danger signs may not approach the CHW with danger signs because the CHW may not be available at the required time	Group counselling during pregnancy with follow-on visits by those who require additional attention Provide incentives for on-time visits Improve links with health workers and local health units
Low CHW uptake, particularly in urban areas and among younger CHW	Women are more educated with more competing demands on their time; CHWs do not feel as welcomed	Increase recognition of CHWs by local leaders in community fora to build confidence in their services Advertise CHW services on the radio and through other media
Cultural barriers around early disclosure of pregnancy	Delayed service delivery	Continued community sensitisation around the importance of early care-seeking Map social networks and identify key informants
Poor linkage between CHWs and local health facility	Women arrive for care when facilities are closed	Improve links between CHWs and local health facilities Ensure CHWs know the services and the times at which these services are available at the facilities

CHWs indicated that at the beginning they were received with mixed feelings by local leaders, who felt threatened. This is despite the fact that the local leaders were involved in the mobilisation for the selection of CHWs. In addition, CHWs were initially viewed as fellow community members with limited expertise to teach about health matters. Specific households in the community regarded as having high status, such as politicians, rich households, and homes of health personnel, were avoided with the thought that they did not really need their service. On a personal level CHWs admitted that despite their desire to fully serve the communities, they also have personal demands on their time, particularly the CHWs in urban areas.

The top performance motivator noted by CHWs was financial benefit, which in the UNEST intervention was limited to a small stipend linked to supervision visits. Other factors mentioned were improved social status (including being called *musawo*, or doctor), a sense of civic responsibility, material benefits such as the kit and t-shirt, and the supervision received. Some of the CHWs saw volunteering as a temporary alternative to unemployment. It offered them an opportunity to put their skills to use in an area where they are needed.

## Discussion

This qualitative study nested in one of the first trials to evaluate home visits during pregnancy and the newborn period in sub-Saharan Africa reveals shifts in the way community members and health workers perceive CHWs within a routine health system structure. Over a short period of time, a new cadre of mostly male CHW was accepted and able to navigate long-held beliefs and practices relating to maternal and newborn care. However, to be successful certain conditions must be met during their selection, training, deployment, supervision, and motivation, in order to overcome the barriers experienced and result in a sustainable, effective community-based service.

Overall social influence, trust, and culture had a strong bearing on community adoption of the behaviours and practices promoted by CHWs. Once trust was established, pregnant women and their families were willing to listen to the CHWs and to respond to referral. A key component to establishing this trust was ensuring male involvement. This was most pronounced in terms of birth preparedness, as men still dominate economic power and related decision making in many households in Uganda (15).

Social influence on health behaviour is a recurrent theme in health promotion literature. This includes the importance of engaging the various social structures that exist in the community, like traditional leadership structures, women's groups, and faith-based groups. Wide engagement has the potential to broaden the ownership of the programme and gain extra support for the CHWs as well as for families (16, 17). The importance of community connections to identify pregnant women and new deliveries suggests that social networks could be strategically mapped and utilised to reach audiences and to disseminate messages. The lack of success in identifying pregnancies among high-risk groups of older and younger women points to the need for CHWs to not only rely on house to house visits, but on other forms of community mobilisation and awareness on services available for families expecting a newborn.

While CHWs were for the most part able to deliver the messages, they learned during training, some of the messages were not accepted as readily as others. Completely changing practices around umbilical cord care, for example, was a challenge. Formative research is critical to identifying the local understanding of chosen messages and barriers to practising uptake. Interventions such as kangaroo mother care for preterm babies, while practised by few participants, generated enthusiastic converts even amongst mothers with normal weight babies and points to an opportunity to use mothers as peer counsellors and champions. Identifying newborns with danger signs and ensuring prompt care-seeking was challenged by the routine visit schedule (i.e. passive case finding). Compliance with referral was thought to be high but also associated with the perceived quality of care at the nearest health facility.

The way CHWs are selected, trained, managed, and supported is central to the quality of services that they deliver (18). Community involvement in the selection process was considered important in CHWs being accepted, a finding confirmed by several other studies (9, 19, 20). However, in urban areas it was difficult to achieve widespread community involvement. Involving local stakeholders in training was also used to ensure buy-in and accountability for the quality and content of the training. The length of training was quite short given the scope of the CHWs’ responsibilities, resulting in a greater emphasis being placed on routine supervision. The immediate deployment of CHWs following training resulted in greater retention of messages, but also meant that CHWs felt unprepared. Initially scheduling monthly supervision was sufficient, until demand for services increased and the CHWs were confident in their role. The use of existing government health workers as supervisors resulted in technically strong support, in addition to creating an additional link between the community and health facility. However, facility-based health workers were not always available, and in some cases motivated CHWs served as peer supervisors. The lack of refresher trainings was compensated for by on-the-job training and mentorship, and this was effective in ensuring knowledge and skill retention (21). If CHWs were included as a permanent feature in the health services, there would be more experienced CHWs who could mentor the newly trained ones for a few weeks, so that when they start in their own communities they have more confidence.

While CHWs noted that financial incentives were key to job performance, intrinsic motivation and job satisfaction were also driving forces. They considered appreciation from community members and the supervision they received from formal health workers as important motivators. Several studies in Nepal and other parts of Africa (Gambia, Ethiopia) show that community acknowledgement is a critical motivation (19, 22, 23). Studies from Tanzania and South Africa also found that money was the top motivator while non-monetary incentives act as enablers (24). A recent meta-analysis on lay health workers found financial compensation to be complicated; some unsalaried lay health workers wanted regular payment, while others were concerned that payment might threaten their social status or lead recipients to question their motives (25). One concern with the volunteer-based position is that some CHWs viewed their role as a temporary alternative to being unemployed. While retention was not a major problem in this study, if run over a longer period of time or scaled up more widely it might pose a problem for CHW reliability.

There are some limitations to this study. Our assessment relied on knowledge, attitudes, and perceptions of CHWs, mothers, health workers, and key stakeholders pertaining to the UNEST intervention. The content of care provided by CHWs was not independently verified through observation of a home visit, although CHW competency is reported elsewhere (21). The CHWs were those who were still active at the end of the implementation phase, so there is no information from CHWs who left their posts. In addition, the caregivers interviewed were limited to those with live children, because of the additional sensitivity involved in talking to parents whose children had died. Despite these limitations, this study examined the implementation experience and identified areas for strengthening the maternal and newborn component of CHW programmes that can be applied in Uganda and similar settings.

This study raises several questions for additional research. Given issues around CHW confidence, the optimal duration of training and intervals for refresher training and/or on-the-job mentorship needs to be further established. The specific challenges faced by CHWs working in urban areas are also an area of very little research, with implications for successful programme scale-up. A major factor that will affect the scale-up of UNEST is the capacity of districts to integrate CHWs into routine systems. In the case of UNEST, district authorities and health workers at health facilities were involved in all aspects of the project, from design to implementation, evaluation, and dissemination. However, owing to limited fiscal and decision space, districts currently do not have additional resources to effectively support, scale-up, and sustain the initiative. Future programmes should explore innovative mechanisms to achieve this, including ensuring that all activities are planned and budgeted for in annual district plans.

## Conclusion

There is potential in the role of CHWs in improving maternal and newborn care in Uganda. However, selection of these workers is a sensitive process that requires a tailored approach for urban compared to rural areas and community involvement in order to foster trust, support, and acceptability. It is essential that CHW training includes problem-solving skills and guidance on integrating technical knowledge with cultural sensitivities, so that service delivery is context-specific. Strengthening links between facility-based health workers and CHWs as well as improving quality of facility care cannot be overlooked.

## References

[1] Lawn JE, Cousens S, Zupan J (2005). 4 million neonatal deaths: When? Where? Why?. Lancet.

[2] WHO (2012). World health statistics.

[3] Lawn JE, Rohde J, Rifkin S, Were M, Paul VK, Chopra M (2008). Alma-Ata 30 years on: revolutionary, relevant, and time to revitalise. Lancet.

[4] UBOS (2011). Uganda demographic and health survey 2011.

[5] Waiswa P, Kallander K, Peterson S, Tomson G, Pariyo GW (2010). Using the three delays model to understand why newborn babies die in eastern Uganda. Trop Med Int Health.

[6] Kumar V, Mohanty S, Kumar A, Misra RP, Santosham M, Awasthi S (2008). Effect of community-based behaviour change management on neonatal mortality in Shivgarh, Uttar Pradesh, India: a cluster-randomised controlled trial. Lancet.

[7] Baqui AH, Arifeen SE, Williams EK, Ahmed S, Mannan I, Rahman SM (2009). Effectiveness of home-based management of newborn infections by community health workers in rural Bangladesh. Pediatr Infect Dis J.

[8] Bang AT, Reddy HM, Deshmukh MD, Baitule SB, Bang RA (2005). Neonatal and infant mortality in the ten years (1993 to 2003) of the Gadchiroli field trial: effect of home-based neonatal care. J Perinatol.

[9] Bhutta ZA, Soofi S, Cousens S, Mohammad S, Memon ZA, Ali I (2011). Improvement of perinatal and newborn care in rural Pakistan through community-based strategies: a cluster-randomised effectiveness trial. Lancet.

[10] World Health Organization, Department of Child and Adolescent Health and Development, UNICEF (2009). Home visits for the newborn child: a strategy to improve survival: WHO/UNICEF Joint Statement.

[11] Waiswa P, Pariyo G, Kallander K, Akuze J, Namazzi G, Ekirapa-Kiracho E (2014). Effect of the Uganda Newborn Study on care-seeking and care practices: a cluster-randomised controlled trial. Glob Health Action.

[12] Waiswa P, Peterson SS, Namazzi G, Ekirapa EK, Naikoba S, Byaruhanga R (2012). The Uganda Newborn Study (UNEST): an effectiveness study on improving newborn health and survival in rural Uganda through a community-based intervention linked to health facilities – study protocol for a cluster randomized controlled trial. Trials.

[13] Health Education and Promotion Division (2010). Village health team strategy and operational guidelines.

[14] Graneheim UH, Lundman B (2004). Qualitative content analysis in nursing research: concepts, procedures and measures to achieve trustworthiness. Nurse Educ Today.

[15] Timša L, Marrone G, Ekirapa E, Waiswa P (2014). Strategies for helping families prepare for birth: experiences from eastern central Uganda. Glob Health Action.

[16] Sauerborn R, Nougtara A, Diesfeld HJ (1989). Low utilization of community health workers: results from a household interview survey in Burkina Faso. Soc Sci Med.

[17] Twumasi PA, Freund PJ (1985). Local politicization of primary health care as an instrument for development: a case study of community health workers in Zambia. Soc Sci Med.

[18] Rifkin SB (2009). Lessons from community participation in health programmes: a review of the post Alma-Ata experience. Int Health.

[19] Amare Y (2009). Non-financial incentives for voluntary community health workers: a qualitative study.

[20] Haines A, Sanders D, Lehmann U, Rowe AK, Lawn JE, Jan S (2007). Achieving child survival goals: potential contribution of community health workers. Lancet.

[21] Kayemba Nalwadda C, Guwatudde D, Waiswa P, Kiguli J, Namazzi G, Namutumba S (2013). Community health workers – a resource for identification and referral of sick newborns in rural Uganda. Trop Med Int Health.

[22] Glenton C, Scheel IB, Pradhan S, Lewin S, Hodgins S, Shrestha V (2010). The female community health volunteer programme in Nepal: decision makers’ perceptions of volunteerism, payment and other incentives. Soc Sci Med.

[23] Nyanzi S, Manneh H, Walraven G (2007). Traditional birth attendants in rural gambia: beyond health to social cohesion. Afr J Reprod Health.

[24] Kironde S, Klaasen S (2002). What motivates lay volunteers in high burden but resource-limited tuberculosis control programmes? Perceptions from Northern Cape province, South Africa. Int J Tuberc Lung Dis.

[25] Glenton C, Colvin CJ, Carlsen B, Swartz A, Lewin S, Noyes J (2013). Barriers and facilitators to the implementation of lay health worker programmes to improve access to maternal and child health: qualitative evidence synthesis. Cochrane Database Syst Rev.

# Sub article: Strategies for helping families prepare for birth: experiences from eastern central Uganda

**DOI:** 10.3402/gha.v8.23969

**Published:** 2015-03-31

**Authors:** Līga Timša, Gaetano Marrone, Elizabeth Ekirapa, Peter Waiswa

**Affiliations:** 1 Global Health, Department of Public Health Sciences, Karolinska Institutet, Stockholm, Sweden; 2 Department of Health Policy, Planning and Management, School of Public Health, Makerere University, Kampala, Uganda

**Keywords:** newborn health, maternal health, community health worker, birth preparedness, antenatal care, Uganda

## Abstract

**Background:**

Promotion of birth preparedness and raising awareness of potential complications is one of the main strategies to enhance the timely utilisation of skilled care at birth and overcome barriers to accessing care during emergencies.

**Objective:**

This study aimed to investigate factors associated with birth preparedness in three districts of eastern central Uganda.

**Design:**

This was a cross-sectional baseline study involving 2,010 women from Iganga [community health worker (CHW) strategy], Buyende (vouchers for transport and services), and Luuka (standard care) districts who had delivered within the past 12 months. ‘Birth prepared’ was defined as women who had taken all of the following three key actions at least 1 week prior to the delivery: 1) chosen where to deliver from; 2) saved money for transport and hospital costs; and 3) bought key birth materials (a clean instrument to cut the cord, a clean thread to tie the cord, cover sheet, and gloves). Logistical regression was performed to assess the association of various independent variables with birth preparedness.

**Results:**

Only about 25% of respondents took all three actions relating to preparing for childbirth, but discrete actions (e.g. financial savings and identification of place to deliver) were taken by 75% of respondents. Variables associated with being prepared for birth were: having four antenatal care (ANC) visits [adjusted odds ratio (OR_A_)=1.42; 95% confidence interval (CI) 1.10–1.83], attendance of ANC during the first (OR_A_=1.94; 95% CI 1.09–3.44) or second trimester (OR_A_=1.87; 95% CI 1.09–3.22), and counselling on danger signs during pregnancy or on place of referral (OR_A_=2.07; 95% CI 1.57–2.74). Other associated variables included being accompanied by one's husband to the place of delivery (OR_A_=1.47; 95% CI 1.15–1.89), higher socio-economic status (OR_A_=2.04; 95% CI 1.38–3.01), and having a regular income (OR_A_=1.83; 95% CI 1.20–2.79). Women from Luuka and Buyende were less likely to have taken three actions compared with women from Iganga (OR_A_=0.72; 95% CI 0.54–0.98 and OR_A_=0.37; 95% CI 0.27–0.51, respectively).

**Conclusions:**

Engaging CHWs and local structures during pregnancy may be an effective strategy in promoting birth preparedness. On the other hand, if not well designed, the use of vouchers could disempower families in their efforts to prepare for birth. Other effective strategies for promoting birth preparedness include early ANC attendance, attending ANC at least four times, and male involvement.

Maternal and child health and well-being have been prioritised in the 15 years through Millennium Development Goals 4 and 5 (1). Significant progress has been achieved globally in reducing mortality amongst mothers and children since 1990 (1), but more innovative strategies are needed to reduce bottlenecks to care-seeking, especially at the time of delivery (2). In Uganda, neonatal mortality remains high and has seen slower declines than maternal and under-5 mortality (3). The country has experienced varying rates of progress in improving the quality of care around the time of delivery, especially in rural areas where services are harder to access (1, 4). Even though most of the women attend antenatal care (ANC) during pregnancy, only 48% of mothers make at least four visits (1, 3). Despite an increase in the number of births assisted by skilled attendants since 1995 (1), the quality of care available may be low, with lack of equipment and skilled providers (3).

In the field of maternal health three basic delays have been identified as major bottlenecks in the provision and use of obstetric (5) and childcare services (6, 7): delays in deciding to seek care, reaching points of care, and receiving quality care at points of delivery. The promotion of birth preparedness and raising awareness of the possible complications and danger signs (DS) is one of the main strategies to overcome these delays and enhance the timely utilisation of skilled care, especially in low-resource settings (8, 9). Birth preparedness is defined as every pregnant woman and her family making a decision beforehand about the place of birth, service provider, and health facility, and having selected key items prior to delivery. It is generally thought that awareness of DS during the various stages of pregnancy and childbirth, and knowledge on when to seek care can reduce the first delay (5). Birth preparedness activities to overcome the second and third delays include planning where to give birth, identifying a birth attendant, planning for transportation, saving money, buying birth materials, and finding a blood donor (9). Each of these components is important and can be crucial in determining mothers’ and newborns’ survival. It is therefore essential to help pregnant women, their families, and the whole community to plan individually and together in order to ensure that no life is lost due to scarcity of birth preparedness items.

In Uganda, promotion of birth preparedness during counselling, and sensitising on DS are key interventions in the Minimum Health Care Package provided through ANC services since 2000 (10). However, it has been reported that counselling on birth preparedness is often not offered or, when offered, the service does not meet the national guidelines (11). In Uganda, only half (51%) of women are informed about pregnancy-related complications during ANC visits (3), with significantly lower coverage amongst rural women and those with less education (11).

To bridge access and demand creation gaps and delays, Uganda is scaling up a number of strategies, including use of community health workers (CHWs) or village health team (VHT) members (4, 10, 12, 13), and vouchers for transport or free services (14), among others. However, there is a dearth of evidence on how various supply and demand side strategies are associated with birth preparedness actions. The aim of this paper was to investigate strategies used to promote birth preparedness in order to increase access to maternal and newborn care. To achieve this, we assessed variables associated with birth preparedness in three districts of eastern central Uganda which use three different implementation strategies for promotion of safe births: use of CHWs (12), use of transport and service vouchers (14), and the routine government system which promotes birth preparedness counselling only at health facilities during ANC. The study was conducted as a baseline for a scale-up programme to improve maternal and newborn care learning from two successful pilot projects (12–15).

## Materials and methods

### Setting and study design

This study used a cross-sectional design in three districts (Buyende, Luuka, and Iganga) of the eastern central region of Uganda. The total fertility rate in this region is above the national average (3). Almost a quarter (24%) of 15–19-year-old females are pregnant or have a child, and 42% of women have an unmet need for family planning (3). During ANC visits just 32% of women are counselled about pregnancy-related DS; 67% deliver with the assistance of health professionals; and 29% have a postnatal check within 22 days of delivery (3).

The three districts were selected because they were close to each other, have people who speak the same language, and have similar cultural practices. However, Iganga has about 4% of its population classified as semi-urban as opposed to the other two that are completely rural (Table 1).

**Table 1:** Demographic and healthcare characteristics for the three study districts

Characteristics	Buyende	Luuka	Iganga
Residence	Rural, remote	Rural	Mostly rural
Population	248,000^a^	243,200^a^	466,200^a^
Health facilities (HF)	21	31	52
HF level III with MNC	4	6	6
HF level IV with MNC	1	1	1
Interventions	Voucher scheme	none	UNEST

MNC=maternal and newborn care.

a From health system assessment 2011 (16).

Buyende district had been home to the Safe Deliveries Study, which promoted access to care through use of vouchers (14). Pregnant women received vouchers for health services (to deliver in the nearest health facility, to attend ANC, and for postnatal care); and for transportation (to use local means, e.g. motorcycle, bicycle) to reach health facilities for ANC, delivery, and one postnatal care visit. The package promoted was comprehensive and included counselling on preparation for childbirth, but in practice was dominated by the vouchers. Provision of these vouchers removed access barriers and led to significantly increased use of maternal, newborn, and obstetric care services (14).

Iganga district was the setting of the Uganda Newborn Study (UNEST) which employed CHWs to promote maternal and newborn care through making home visits (12). CHWs conducted five home visits: two during the pregnancy, and three during the first week after delivery. During each pregnancy visit, CHWs counselled and showed women and families items they needed to have or steps to take as part of their birth preparations. In addition, they informed women about DS during pregnancy, and if present, the CHWs referred women to a health facility. On subsequent visits CHWs followed up how many and which actions had been taken to prepare for childbirth (12).

Luuka district had no additional intervention beyond the standard of care provided by the routine package of the Ministry of Health.

### Sampling and data collection

Multistage sampling was performed by selecting parishes at the subcounty level and then villages within these parishes. In total, 17 parishes and 39 villages were included in the study (Fig. 1). Buyende had five subcounties and one parish was randomly selected. From each parish, subsequently three villages were chosen and about 53 mothers were interviewed from each village. Luuka had seven subcounties and seven parishes randomly chosen; in each parish two villages were selected for interviewing (about 56 mothers from each village). In Iganga, we randomly selected five parishes from five subcounties; and two villages per parish were picked (about 40 mothers from each village).

**Fig. 1:** Sampling procedure in three study districts in eastern central Uganda.

All women who had delivered a child in the past 12 months and were living in one of the selected villages were listed and selected. Exclusion criteria included a woman being ill at the time of the study, but we found none fitting that criterion. In total, 2,011 women were interviewed, but one woman was excluded from further analysis due to lack of data on outcome variables. Informed consent was obtained from the participants. The study was approved by the Makerere University School of Public Health Institutional Review Board and the Uganda National Council of Science and Technology.

A structured questionnaire with information on ANC, delivery and postnatal care, family planning, socio-demographic factors, and socio-economic data was used. The survey tool was translated into the local Lusoga language and was piloted and changed accordingly. The questionnaire was created following validated JHPIEGO guidelines (9). Thirty locally recruited field assistants with a minimum of secondary school education underwent a 3-day training (including 1 day of piloting) on data collection. Each interview lasted for about an hour. Data were collected from October 2011 to January 2012.

### Data analysis

Data were coded, entered, cleaned, and analysed using SPSS version 21.0 (17). A ‘birth prepared’ woman was defined as a woman who had taken three actions at least 1 week prior to the delivery: 1) chosen where to deliver; 2) saved money for transport and hospital costs; and 3) bought key birth materials (razor, thread, cover sheet, and gloves). These actions do not constitute the sum total of possible activities related to birth preparedness, but were chosen for analysis purposes and because they aligned with messages promoted in the three different settings during pregnancy.

If the respondent or her husband's main occupation was running a business or having salaried work, then she was coded as having ‘regular income’. On the other hand, if the main occupation of both respondent and husband was having a daily-wage job or being a farmer, then she was coded as having ‘irregular income’. Marital status was dichotomised within the categories ‘married’ and ‘not married’; widows, single, and divorced women belonged to the latter group. Education had three categories: ‘no formal education’, ‘primary’ which included finished or started primary schooling, and ‘secondary’ which contained secondary and higher schooling. The age groups of respondents were stratified as follows: under the age of 19 (less experienced women); 20–39 years old; older than 40 years (women who are experienced, but might be already in a risk group); and women who did not know their age.

The following were the independent variables: district, number of household members, number of children, sequence of pregnancy, marital status, mother's age group, religion, mother's education, husband's education, mother's income, husband's income, receiving information about DS, number of ANC visits, trimester during which the first ANC visit was undertaken, receiving care from a healthcare provider during the pregnancy, quintile of asset ownership, decision maker, and accompanied by husband to the place of delivery. For logistical reasons, no further verification of responses was done.

First, absolute and relative frequencies for all categorical dependent and independent variables were calculated. Second, the relationship between the outcome (birth preparedness) and selected independent variables was tested by Pearson's Chi-square test. Subsequently, all variables with *p* value<0.2 were inserted in a stepwise logistical regression model to investigate the association by obtaining the adjusted odds ratios (OR_A_) with 95% confidence intervals (CIs), thereby adjusting for confounders and effect modifiers likely to influence the outcomes. Absence of multi-colinearity between independent variables in the final model was tested and confirmed. Four different multivariable logistical regression models were tested: all cases together and one for each of the three study districts separately.

## Results

### Descriptive results

Most women in the study sample were married, more than half were Christian, and approximately one-third were Muslim (Table 2). The median age of the respondents was 25±6.6 years (ranging from 12 to 53 years). The proportion of husbands with at least secondary education was double the proportion of women with this level of education (39.1 vs. 20.2%). Most respondents and their husbands had irregular income, and 12.7% of women had no formal education.

**Table 2:** Socio-demographic characteristics of the sample

Characteristic	Frequency *n* (%)
District (*n*=2,010)	
Buyende	819 (40.7)
Luuka	797 (39.7)
Iganga	394 (19.6)
Marital status (*n*=2,007)	
Not married	179 (8.9)
Married	1,828 (91.1)
Respondent's age (*n*=2,006)	
≤ 19 years	326 (16.3)
20–39 years	1,507 (75.1)
≥ 40 years	69 (3.4)
Do not know	104 (5.2)
Religion (*n*=2,009)	
Christian	1,379 (68.6)
Muslim	612 (30.4)
Other	18 (0.9)
Respondent's education (*n*=2,008)	
No school	256 (12.7)
Primary	1,347 (67.1)
≥ Secondary	405 (20.2)
Respondent's income (*n*=2,006)	
Irregular	1,861 (92.8)
Regular	145 (7.2)
Husband's education (*n*=1,753)	
No school	108 (6.2)
Primary	959 (54.7)
≥ Secondary	686 (39.1)
Husband's income (*n*=1,753)	
Irregular	1,334 (76.1)
Regular	419 (23.9)

Median parity of the sample was 4±2.8 (range 1–15 children). Overall, 16.4% were primiparous mothers (Table 3). Median ANC attendance was 3±1.4 times (range 0–17 visits). Less than half of the women attended an ANC clinic at least four times, and just one-third of respondents went for their first session during the first trimester. One-fifth of pregnant women were told about the possible complications during the pregnancy period (2% of respondents were told about DS and 18% got information about DS and where to seek help). Very few respondents reported having been visited by a healthcare provider (doctor, nurse, CHW, or traditional birth attendant) during their pregnancy. In most families, the husband was reported as the decision maker regarding the woman's and newborn's health, and just 7.8% of women stated that they decided for themselves when to seek care and from where. Six of ten respondents reported they were accompanied by their husbands to the place of delivery (Table 3).

**Table 3:** Reproductive health and socio-economic patterns of the sample

Characteristics	Frequency *n* (%)
Number of children (*n*=2,009)	
1	329 (16.4)
2–3	597 (29.7)
4–5	434 (21.6)
≥6	649 (32.3)
ANC attendance (*n*=1,990)	
0–3 times	1,057 (53.1)
≥4 times	933 (46.9)
When was first ANC visit – trimester (*n*=1,967)	
1st	668 (34.0)
2nd	1,131 (57.5)
3rd	168 (8.5)
Told about pregnancy DS and where to seek help if they occur (*n*=2,005)	
None	1,595 (79.6)
Yes – DS	43 (2.1)
Yes – both	367 (18.3)
Visited by healthcare provider during pregnancy (*n*=2,010)	
No visits	1,935 (96.3)
Health professional	38 (1.9)
CHW/VHT	32 (1.6)
Other	5 (0.2)
Delivered at health facility (*n*=2,009)	
No	586 (29.2)
Yes	1,418 (70.8)
Decision making regarding mother's and newborn's health (*n*=2,008)	
Herself	157 (7.8)
Husband	1,674 (83.4)
Together	29 (1.4)
Other	148 (7.4)
Escorted by husband to place of delivery (*n*=1,887)	
No	739 (39.2)
Yes	1,148 (60.8)

CHW/VHT=community health worker/village health team.

### Birth preparedness across districts

As there were specific interventions carried out in the districts, a comparison was performed to evaluate distribution of different independent variables and birth preparedness in three study areas. Statistically significant differences observed across the districts include ANC attendance of at least four visits, first ANC session during the first trimester, a home visit during the pregnancy period, and receiving counselling on pregnancy-related DS, which were more common in Iganga than in the other districts (Fig. 2). Women from Iganga (CHW intervention) were more likely to take all three birth preparedness steps compared to women in Luuka (standard care) or Buyende (vouchers scheme) (Fig. 3). Although very few women performed all three preparation activities (Table 4), discrete birth preparedness actions were taken frequently. Fewer than 7% of women had not taken any actions to prepare for delivery.

**Fig. 2:** ANC practices among respondents (%) of three study districts. **DSP**=danger signs during pregnancy; *statistically significant differences (*p*<0.05).

**Fig. 3:** Respondents (%) taking individual and all three birth preparedness actions in the study districts. *Statistically significant differences (*p*<0.05).

**Table 4:** Birth preparedness^a^ characteristics

Characteristics	Frequency *n* (%)
Birth prepared^b^ (*n*=2,010)
Yes	497 (24.7)
No	1,513 (75.3)
Steps taken (*n*=2,010)
None	148 (7.4)
1 out of 3 steps	465 (23.1)
2 out of 3 steps	900 (44.8)
All 3 steps	497 (24.7)

a Birth preparedness steps: Identification of location for the delivery, saving for transport and other expenses, and procurement of four essential birth materials (razor, thread, sheet, and gloves).

b ‘Birth prepared’ was deemed a woman who had performed all three steps of birth preparedness by not later than 1 week before delivery.

Variables associated with birth preparedness in the logistical regression analyses varied among the three districts. The determinants of birth preparedness in Buyende district included attending ANC four times or more, counselling on DS and place of referral, contact with a healthcare professional during the pregnancy, coming from a household with highest asset ownership, and the respondent having a regular income (Table 5). However, if the husband was reported as the only decision maker regarding health issues, the odds of a woman being prepared for childbirth were reduced.

**Table 5:** Multivariable logistic regression analysis for associations with birth preparedness

	Pooled sample			Buyende			Luuka			Iganga		
			
	OR_A_	95% CI	*p*	OR_A_	95% CI	*p*	OR_A_	95% CI	*p*	OR_A_	95% CI	*p*
District
Buyende	**0.37**	0.27–0.51	0.0001									
Luuka	**0.72**	0.54–0.98	0.035									
Iganga	Ref											
ANC attendance
0–3 times	Ref			Ref			a			Ref		
≥ 4 times	**1.42**	1.10–1.83	0.007	**2.20**	1.38–3.51	0.001				**1.66**	1.05–2.61	0.030
When was 1st ANC visit – trimester
1st	**1.94**	1.09–3.44	0.024	a			**3.23**	1.22–8.60	0.019	a		
2nd	**1.87**	1.09–3.22	0.023				2.59	0.98–6.82	0.054			
3rd	Ref						Ref					
Told about pregnancy DS and where to seek help if they occur
None	Ref			Ref			Ref			a		
Yes – DS	0.49	0.20–1.20	0.119	0.00	0.00	0.999	0.41	0.12–1.42	0.158			
Yes – both	**2.07**	1.57–2.74	0.0001	**2.04**	1.21–3.44	0.007	**2.15**	1.41–3.28	0.0001			
Visited by healthcare provider during pregnancy
No visits	a			Ref			a			a		
Health professional				**14.40**	2.70–76.84	0.002						
CHW/VHT				0.00	0.00	0.999						
Other				0.00	0.00	1.000						
Decision making regarding mother's and newborn's health
Herself	a			Ref			b			a		
Husband				**0.29**	0.14–0.61	0.001						
Partners together				0.24	0.04–1.38	0.109						
Others				0.40	0.13–1.18	0.097						
Escorted by husband to the place of delivery
No	Ref			a			Ref			a		
Yes	**1.47**	1.15–1.89	0.003				**1.70**	1.16–2.50	0.007			
Quintiles of asset ownership index
1 (poorest)	Ref			Ref			a			a		
2	1.04	0.69–1.57	0.859	1.91	0.95–3.83	0.070						
3	1.12	0.74–1.69	0.589	1.43	0.67–3.06	0.361						
4	1.26	0.84–1.89	0.265	1.94	0.95–3.96	0.07						
5	**2.04**	1.38–3.01	0.0001	**3.69**	1.80–7.60	0.0001						
Respondent's income
Irregular	Ref			Ref			a			a		
Regular	**1.83**	1.20–2.79	0.005	**2.25**	1.09–4.64	0.029						
Husband's income
Irregular	a			a			a			Ref		
Regular										**2.04**	1.25–3.31	0.004

a Not significantly associated with the outcome

b OR and 95% CI with *p* value<0.05 are highlighted in bold.

In Luuka, variables that predicted birth preparedness were ANC visit during the first trimester, counselling on DS and place of referral, and husband escorting wife to the place of delivery. In Iganga, district birth preparedness was associated with at least four ANC sessions and the husband having a regular income.

When all cases were analysed together, we found the following to be independent variables associated with birth preparedness. Those coming from Iganga reported attending at least four ANC sessions, having an initial ANC visit during the first or the second trimester, being counselled on pregnancy-related DS and place of referral in case of complications, being accompanied by the husband to the place of delivery, coming from a household with highest asset ownership, and regular income.

## Discussion

Our findings show that the use of CHWs is an effective strategy in promoting birth preparedness. On the other hand, supply-side strategies such as use of vouchers to reduce delays in access to care and increase utilisation may hinder families from preparing for birth, if not well designed. A study in Western Uganda found that a low level of DS awareness among respondents combined with lack of preparation led to lower health-seeking behaviour, which could result in increased maternal and newborn mortality and morbidity (18). However, overall we found birth preparedness to be low in the three districts. Although discrete steps were taken by 75% of all women, the three actions under consideration were only carried out by 25% of respondents. In one study in Western Uganda, 91% of participants reportedly had saved money for delivery (18). This is high in comparison to other studies, where savings ranged from 36% in Ethiopia to 83% in Burkina Faso (19–23).The proportion of women taking all three identified actions was similar to another study in Western Uganda, which found 35% of respondents accomplishing at least three out of four actions (18).

We observed that three-fourth of the women in our sample population had financial savings, slightly more in Iganga and Luuka as compared to Buyende. The fact that areas with CHWs had better birth preparedness than areas with vouchers is interesting. It is suggestive that CHWs are able to change long-held cultural norms such as not preparing for the unborn child, probably because they are able to engage more on a one-to-one basis with individual women and with families on a more sustained basis, as compared to health workers in health facilities during ANC. Other findings also show the effectiveness of CHWs in terms of increasing referral compliance, and that training and support supervision can make them highly competent (13).

We identified that areas with vouchers had lower birth preparedness compared to those with CHWs. By nature the voucher scheme has one major aim, which is increasing utilisation of health facilities; it rarely includes empowerment of families to prepare for birth. Future voucher schemes need to be designed with empowerment of women, families, and communities as part of a comprehensive package; otherwise they can lead to dependency.

Besides use of CHWs, there were other effective strategies for promoting birth preparedness, including attending ANC at least four times, having an initial ANC visit during the first or the second trimester, being counselled on pregnancy-related DS, and male involvement. Obviously, there is a time limit for actions when ANC is sought later in pregnancy. However, only 34% of women across the three districts had their first ANC visit during the first trimester, and significant sociocultural barriers exist around revealing pregnancy early (11, 24). Studies in Ethiopia (22) and India (25) also found that ANC attendance of at least four times was associated with being prepared for birth.

Socio-economic status of the family was associated with being prepared for delivery in our study, that is, respondents or their husbands with regular income and those women from households in the highest asset ownership quintile. Education level did not contribute to birth preparedness in this study population, despite its importance elsewhere (18, 20, 25, 26). This could be due to the low levels of education generally across the population.

This study provides important information about the characteristics associated with care-seeking around the crucial time of childbirth in eastern Uganda. Two districts with and one district without any prior interventions were involved. However, there are several limitations to our study. All data collected relied on the respondents’ ability to recall their actions. To reduce recall bias only women who had delivered within the past 12 months were invited to participate. The same procedure was used in other studies in Western Uganda and elsewhere (26–28). Additional reporting bias could have occurred by the respondents giving answers perceived to be the best practice. The cross-sectional design of this study does not provide any causality inferences between the outcome and various independent variables, it only demonstrates associations.

## Conclusions

Engaging CHWs and other local structures may be an effective strategy in promoting birth preparedness. On the other hand, if not well designed, the use of vouchers could disempower families from making preparations for birth other than acquiring a voucher. Other effective strategies for promoting birth preparedness in this setting include early ANC attendance, attending ANC at least four times, and male involvement.

## References

[1] WHO, UNICEF (2012). Countdown to 2015. Maternal, newborn and child survival. Building a future for women and children. http://www.countdown2015mnch.org/documents/2012Report/2012-Complete.pdf.

[2] Darmstadt GL, Kinney MV, Chopra M, Cousens S, Kak L, Paul VK (2014). Who has been caring for the baby?. Lancet.

[3] UBOS, (Uganda Bureau of Statistics), ICF International Inc (2012). Uganda demographic and health survey 2011. http://www.measuredhs.com/pubs/pdf/FR264/FR264.pdf.

[4] Mbonye AK, Sentongo M, Mukasa GK, Byaruhanga R, Sentumbwe-Mugisa O, Waiswa P (2012). Newborn survival in Uganda: a decade of change and future implications. Health Policy Plan.

[5] Thaddeus S, Maine D (1994). Too far to walk: maternal mortality in context. Soc Sci Med.

[6] Mbaruku G, van Roosmalen J, Kimondo I, Bilango F, Bergstrom S (2009). Perinatal audit using the 3-delays model in Western Tanzania. Int J Gynaecol Obstet.

[7] Waiswa P, Kallander K, Peterson S, Tomson G, Pariyo GW (2010). Using the three delays model to understand why newborn babies die in Eastern Uganda. Trop Med Int Health.

[8] Parlato P, Darmstadt L, Tinker A (2004). Saving newborn lives. Tools for newborn health. Qualitative research to improve newborn care practices. http://www.chnri.org/resources/1.%20Learning%20Resource%20Material/Qualitative%20Research/Guides/Neonatal%20methodology.pdf.

[9] JHPIEGO, Maternal and Newborn health program (2004). Monitoring birth preparedness and complication readiness. Tools and indicators for maternal and newborn health. http://www.jhpiego.org/files/BPCRtoolkit.pdf.

[10] Waiswa P, Kemigisa M, Kiguli J, Naikoba S, Pariyo GW, Peterson S (2008). Acceptability of evidence-based neonatal care practices in rural Uganda – implications for programming. BMC Pregnancy Childbirth.

[11] MoH-Uganda, (Ministry of Health Uganda), Save the Child, UNICEF, WHO (2008). Situation analysis of newborn health in Uganda: Current status and opportunities to improve care and survival. http://www.healthynewbornnetwork.org/sites/default/files/resources/Situation%20Analysis%20of%20Newborn%20health%20in%20Uganda.pdf.

[12] Waiswa P, Peterson SS, Namazzi G, Ekirapa EK, Naikoba S, Byaruhanga R (2012). The Uganda Newborn Study (UNEST): an effectiveness study on improving newborn health and survival in rural Uganda through a community-based intervention linked to health facilities – study protocol for a cluster randomized controlled trial. Trials.

[13] Kayemba-Nalwadda C, Guwatudde D, Waiswa P, Kiguli J, Namazzi G, Namutumba S (2013). Community health workers – a resource for identification and referral of sick newborns in rural Uganda. Trop Med Int Health.

[14] Ekirapa-Kiracho E, Waiswa P, Rahman MH, Makumbi F, Kiwanuka N, Okui O (2011). Increasing access to institutional deliveries using demand and supply side incentives: early results from a quasi-experimental study. BMC Int Health Hum Rights.

[15] Nalwadda CK, Waiswa P, Kiguli J, Namazzi G, Namutamba S, Tomson G (2013). High compliance with newborn community-to-facility referral in Eastern Uganda: an opportunity to improve newborn survival. PLoS One.

[16] MoH-Uganda, (Ministry of Health Uganda), Health Systems 20/20, Makarere University School of Public Health (2012). Uganda health system assessment 2011. http://health.go.ug/docs/hsa.pdf.

[17] SPSS-PASW, version 121.

[18] Kabakyenga JK, Ostergren PO, Turyakira E, Pettersson KO (2011). Knowledge of obstetric danger signs and birth preparedness practices among women in rural Uganda. Reprod Health.

[19] Moran AC, Sangli G, Dineen R, Rawlins B, Yameogo M, Baya B (2006). Birth-preparedness for maternal health: findings from Koupela district, Burkina Faso. J Health Popul Nutr.

[20] Ekabua JE, Ekabua KJ, Odusolu P, Agan TU, Iklaki CU, Etokidem AJ (2011). Awareness of birth preparedness and complication readiness in Southeastern Nigeria. ISRN Obstet Gynecol.

[21] Wiegers TA, Boerma WG, de Haan O (2010). Maternity care and birth preparedness in rural Kyrgyzstan and Tajikistan. Sex Reprod Health.

[22] Hailu M, Gebremariam A, Alemseged F, Deribe K (2011). Birth preparedness and complication readiness among pregnant women in Southern Ethiopia. PLoS One.

[23] Turan JM, Tesfagiorghis M, Polan ML (2011). Evaluation of a community intervention for promotion of safe motherhood in Eritrea. J Midwifery Womens Health.

[24] Neonatal Mortality Formative Research Working Group (2008). Developing community-based intervention strategies to save newborn lives: lessons learned from formative research in five countries. J Perinatol.

[25] Agarwal S, Sethi V, Srivastava K, Jha P, Baqui A (2010). Birth preparedness and complication readiness among slum women in Indore city, India. J Health Popul Nutr.

[26] Pembe AB, Urassa DP, Carlstedt A, Lindmark G, Nystrom L, Darj E (2009). Rural Tanzanian women's awareness of danger signs of obstetric complications. BMC Pregnancy Childbirth.

[27] Kabakyenga JK, Ostergren PO, Turyakira E, Pettersson KO (2012). Influence of birth preparedness, decision-making on location of birth and assistance by skilled birth attendants among women in South-Western Uganda. PLoS One.

[28] Dongre AR, Deshmukh PR, Garg BS (2009). Awareness and health care seeking for newborn danger signs among mothers in peri-urban Wardha. Indian J Pediatr.

# Sub article: Strengthening health facilities for maternal and newborn care: experiences from rural eastern Uganda

**DOI:** 10.3402/gha.v8.24271

**Published:** 2015-03-31

**Authors:** Gertrude Namazzi, Peter Waiswa, Margaret Nakakeeto, Victoria K. Nakibuuka, Sarah Namutamba, Maria Najjemba, Ruth Namusaabi, Abner Tagoola, Grace Nakate, Judith Ajeani, Stefan Peterson, Romano N. Byaruhanga

**Affiliations:** 1 School of Public Health, College of Health Science, Makerere University, Kampala, Uganda; 2 INDEPTH, Iganga/Mayuge Health Demographic Surveillance Site, Kampala, Uganda; 3 Global Health, Department of Public Health Sciences, Karolinska Institutet, Stockholm, Sweden; 4 Kampala Children's Hospital, Kampala, Uganda; 5 St. Raphael of St. Francis Hospital, Nsambya, Kampala, Uganda; 6 School of Medicine, Uganda Martyrs University, Nkozi, Kampala, Uganda; 7 Ministry of Health, Kampala, Uganda; 8 International Maternal and Child Health Unit, Department of Women's and Children's Health, Uppsala University, Uppsala, Sweden

**Keywords:** health system strengthening, maternal care, newborn care, neonatal mortality, kangaroo mother care, Uganda

## Abstract

**Background:**

In Uganda maternal and neonatal mortality remains high due to a number of factors, including poor quality of care at health facilities.

**Objective:**

This paper describes the experience of building capacity for maternal and newborn care at a district hospital and lower-level health facilities in eastern Uganda within the existing system parameters and a robust community outreach programme.

**Design:**

This health system strengthening study, part of the Uganda Newborn Study (UNEST), aimed to increase frontline health worker capacity through district-led training, support supervision, and mentoring at one district hospital and 19 lower-level facilities. A once-off supply of essential medicines and equipment was provided to address immediate critical gaps. Health workers were empowered to requisition subsequent supplies through use of district resources. Minimal infrastructure adjustments were provided. Quantitative data collection was done within routine process monitoring and qualitative data were collected during support supervision visits. We use the World Health Organization Health System Building Blocks to describe the process of district-led health facility strengthening.

**Results:**

Seventy two per cent of eligible health workers were trained. The mean post-training knowledge score was 68% compared to 32% in the pre-training test, and 80% 1 year later. Health worker skills and competencies in care of high-risk babies improved following support supervision and mentoring. Health facility deliveries increased from 3,151 to 4,115 (a 30% increase) in 2 years. Of 547 preterm babies admitted to the newly introduced kangaroo mother care (KMC) unit, 85% were discharged alive to continue KMC at home. There was a non-significant declining trend for in-hospital neonatal deaths across the 2-year study period. While equipment levels remained high after initial improvement efforts, maintaining supply of even the most basic medications was a challenge, with less than 40% of health facilities reporting no stock-outs.

**Conclusion:**

Health system strengthening for care at birth and the newborn period is possible even in low-resource settings and can be associated with improved utilisation and outcomes. Through a participatory process with wide engagement, training, and improvements to support supervision and logistics, health workers were able to change behaviours and practices for maternal and newborn care. Local solutions are needed to ensure sustainability of medical commodities.

Neonatal conditions contribute approximately 10% to the global burden of disease, more than three times that of HIV (1). In Uganda this burden is doubled, with perinatal and maternal conditions contributing an estimated 20% of the overall burden of disease (2). While demand for facility deliveries seems to be increasing, many women still deliver in the community with the assistance of unskilled birth attendants such as traditional birth attendants, relatives, or even alone. After delivery, there are a number of traditional and socio-economic barriers to seeking care outside of the home for both healthy and sick newborns, as well as for mothers (3, 4). For those who do seek care at a health facility, substandard obstetric and neonatal care continues to be a factor in poor maternal and newborn outcomes (5, 6).

The importance of an integrated continuum of care from pre-pregnancy through childhood across levels of service delivery from household to hospital is well known (7). However, health system bottlenecks at all levels lead to low coverage of many priority interventions through poor coordination, weak infrastructure, shortage of trained and motivated health workers, low uptake of available capacity, and household resistance to recommended practices (8, 9). Within the World Health Organization Health System Building Blocks Framework, there are six components, namely health workforce, service delivery, information, supplies, financing, and leadership, which allow for the systematic identification of gaps within the system (10). This multidimensional approach helps identify synergistic effects of complementary interventions from facility level to district and up to national level, with careful monitoring and steering of dynamic and interrelated processes.

The Uganda Newborn Study (UNEST) was conceived to adapt a community care package for maternal and newborn health and evaluate its effect on maternal and newborn care outcomes in order to inform policy and scale-up in Uganda. When formative research revealed that poor-quality health facility care was the second leading reason for newborn deaths (4), it was determined that the intervention package needed to go beyond merely introducing a community-level cadre. Health facilities lacked infrastructure, equipment, drugs, supplies and protocols for newborn care, and the majority of health workers lacked knowledge and skills to care for vulnerable neonates (4). An increase in demand for services would not necessarily save lives without commensurate improvements in health facility quality.

Within Ugandan policy, one general hospital is supposed to serve approximately 500,000 people, while health sub-districts administer lower-level health facilities, including health centre (HC) levels II, III, and IV.; Level II HCs are small, outpatient-only units which can provide a first dose of antibiotics to sick newborns and referrals; level III HCs conduct births, manage newborn illness, and provide laboratory services; and Level IV HCs function as small hospitals, which should be equipped for emergency obstetric care. Private and faith-based organisations own 41% of hospitals and 22% of lower-level HCs. Private not-for-profit facilities receive government subsidies to expand care to rural areas (11).

Integration of the targeted interventions into a health system is difficult and complex, especially in a weak health system (12). Yet, strengthening health systems to deliver services equitably and efficiently is crucial for achieving improved maternal and newborn care. This paper describes the health systems strengthening process used to improve quality of care across 20 health facilities in rural eastern Uganda, and assesses its effect on the outcome of high-risk newborn babies. This paper is the fifth in a series on the UNEST.

## Methods

The study was nested within the UNEST randomised control trial whose details and results are described elsewhere (13, 14). Briefly, within UNEST, villages were either randomised to the intervention (a trained community health worker (CHW) making home visits to meet pregnant and newly delivered mothers) or the control (existing standard of care). UNEST was implemented in the Iganga/Mayuge Health and Demographic Surveillance Site with a population of around 70,000.

For the health systems strengthening component, 20 health facilities within the district were targeted from 2009 through 2011, including one hospital, one HC IV, six HC III, and twelve HC II, capturing both public and private health facilities. The health facilities catered to clients from both control and intervention areas, as well as outside of the study districts. For example, the catchment population of the hospital is approximately 1.5 million. The intervention was implemented together with district and Ministry of Health leadership to identify gaps in service delivery and to make changes which would improve the responsiveness of the health system to maternal and newborn complications.

We describe and analyse the health system changes according to the WHO Health System Building Blocks, including health workforce, service delivery, health information systems, equipment and supplies, with leadership and finance combined (10). Quantitative data were collected by mentorship teams from all health facilities from the routine health management information system (HMIS) on a quarterly basis. Baseline data were extracted for the 2 years prior to implementation. Quantitative data were obtained quarterly using a structured tool based on the national standards for newborn healthcare services (15) which was pretested prior to use.

An additional data capture form on service utilisation and care of the sick newborn babies and preterm babies using kangaroo mother care (KMC) was developed for use at the hospital. Key indicators collected included availability of basic essential medicines and equipment (e.g. newborn resuscitation equipment, neonatal weighing scale, thermometer, fetoscope, bulb syringe, blood pressure machine, stethoscope, delivery kit, steriliser, and light source); and availability of essential medicines (e.g. ampicillin, gentamycin, vitamin K, tetracycline eye ointment, Fansidar, oxytocin, and magnesium sulphate). Data on the number of deliveries, stillbirths, neonatal deaths, patients discharged, as well as health worker training and turnover were collected. In instances where routine data were incomplete, stock cards were used to complete the section on medicines. Data were entered into Microsoft Excel spreadsheets and checked for completeness, then exported to Epidata version 3.1 for descriptive analysis.

As part of the quarterly process monitoring, qualitative data were also collected. The study team documented implementation experiences and contextual factors and events (e.g. new facility management, major donor input to a facility or community) that might influence study outcomes. Field notes and reports from the support supervision and mentorship visits were reviewed on a quarterly basis. These activities were the basis for information on performance of service providers.

### Health workforce

Health workers at the targeted facilities were identified and their level of formal training documented. With involvement of facility administration and district leadership, frontline workers attended in-service refresher training in obstetric and newborn care, modified for the level of service delivery. Four training sessions were conducted between July 2009 and June 2010. Each training session lasted 6 days and used an integrated training maternal and newborn manual developed by the study team, reviewed by members of national midwifery, obstetric and paediatric professional associations, and endorsed by local stakeholders and the Ministry of Health. The training package addressed the major causes of maternal and newborn deaths and morbidity in Uganda. Modules included goal-oriented antenatal care, managing maternal complications, infection prevention, managing normal labour and partograph use, neonatal resuscitation, care of the sick newborn, and extra care for the small baby using KMC. Knowledge tests were given before and after training, as well as after 1 year of implementation. In addition, training in maternal and perinatal mortality auditing was provided according to Ministry of Health guidelines (16).

### Service delivery

In order to improve service delivery, the organisation of wards and patient flow were examined for critical bottlenecks. Interventions involved the redesigning and reorganisation of space to cater for labour management and newborn care. Screens were introduced for privacy in the labour ward. In the hospital, space was specifically designated for KMC by screening off two beds and placing posters and job aids on the walls. Later, with district buy-in, a modest special care neonatal unit was built adjacent to the labour and delivery ward to care for high-risk newborns.

Support supervision and mentoring was also a key component of improving service delivery. Teams of national-level and district-based health workers carried out quarterly outreach visits to facilities that were conducting deliveries. The co-opted mentoring team comprised a paediatrician, an obstetrician, a midwife, and district technical officers. The supervision visits included assessment of skills, support for problem solving with frontline staff to identify gaps and solutions, and development and review of health facility work plans. In initial outreach visits, an assessment of the physical layout of the sites was done with recommendations for reorganisation, if needed. Maternal and perinatal death review committees were established with the expectation of biweekly meetings. The minutes were reviewed during supervision visits. Newly introduced interventions, such as neonatal resuscitation using bag and mask, KMC, and treatment of neonatal sepsis, were given particular attention during mentorship visits.

### Equipment and supplies

Sensitisation meetings were held with health administration officials at different levels to highlight maternal and newborn care needs and the importance of timely procurement and dispatch of supplies. Health facility in-charges and district health teams were engaged to ensure future supplies are provided for through the routine channels. A once-off supply of essential medicines, medical supplies, and equipment was provided as a catalyst to address immediate gaps. Supplies included delivery beds, Ambu bags, newborn-size masks, bulb syringes, nasal prongs, cannulas, and oxygen concentrators. Partographs and standard management protocols and guidelines were printed and distributed targeting major causes and solutions of neonatal morbidity and mortality.

### Health information

The status of the patient charts and registers was assessed at baseline. File folders for inpatient care in maternity and paediatric wards approved by the Ministry of Health were reintroduced to standardise record-keeping and to facilitate data availability for the audit sessions. Where lacking, registers and summary sheets were printed and provided. At the hospital, new registers were developed and provided for the KMC unit, the neonatal special care unit, and the neonatal room on the paediatric ward. Forms for maternal death notification and maternal and perinatal mortality audit were also provided. During support supervision, health workers and records staff were consulted about the completeness and accuracy of the information collected. The HMIS data were extracted on a quarterly basis by the support supervision team.

### Leadership/governance and finance

Sensitisation of district leaders, CHWs, and various stakeholders on the importance of maternal and newborn health was an early intervention milestone, with the assumption that local buy-in was crucial. Managers at health units were encouraged to prioritise procurement of basic supplies for obstetric and newborn care, and were given strategies to avoid stock-outs, support for annual budgeting, and training on quality improvement. The identification and development of local champions was a key strategy. Individuals were identified during early trainings and later involved in the co-opted mentorship teams, learning visits, trainings, reviews, and dissemination activities.

## Results

Amongst the facilities assessed, all offered antenatal care, well-baby checkups, and served as a point of contact for sick newborns. Fourteen of the facilities (70%) conducted deliveries. Although current Ugandan policy only mandates delivery services at level III HCs and above, it was found that half of the level II HCs were also conducting deliveries. Only the hospital had facilities for admission and treatment with antibiotics for sick neonates.

During the implementation period, there was an increase in the number of deliveries conducted at health facilities within the study period, from 3,151 at the beginning of the intervention in the second half of 2009, to about 4,115 deliveries (an increase of 30%) at the end of the study (Fig. 1). Despite this increase, there was no change in the proportion of births resulting in caesarean section, which was 12% at the beginning of implementation in 2009 and 13% by the end of implementation. The rate of preterm birth was 8% in deliveries occurring in health units. The number of sick neonates from the community admitted to the neonatal unit also increased. A total of 249 sick newborn babies were admitted to the paediatric neonatal unit during the study period. The in-hospital neonatal mortality rate amongst admitted sick neonates declined from 17% in the first quarter to 9% in the last quarter, although the trend was non-significant.

**Fig. 1:** Health facility deliveries from Iganga/Mayuge Demographic Surveillance Site.

### Health workforce

Over the 2-year implementation period, four in-service training sessions were conducted. Overall, 72% (105/146) of targeted health providers were trained (Table 1). The majority of trainees were midwives, nurses, and nursing assistants. Ten clinical officers and one medical officer also received training (Fig. 2). A higher proportion of the staff at lower-level health units was trained, although absolute numbers were higher for hospitals.

**Fig. 2:** Proportion of health workers trained in maternal and newborn care by cadre.

**Table 1:** Distribution of health facilities by level and eligible health workers trained

Health facility level	Number of health facilities	Total number of health workers	Health workers trained (%)
HC II	12	33	25 (76)
HC III	6	49	34 (69)
HC IV	1	14	12 (86)
Hospital	1	50	34 (68)
Total	20	146	105 (72)

HC *=* Health centre.

Results from pre-training and post-training tests demonstrated improvement in knowledge of maternal and newborn care. The mean pre-training score was 32% compared to a post-training score of 68%. After 1 year of implementation, the mean score was 80%. While skill retention was not systematically assessed, this was documented in supervision notes. Supervision reports also revealed that health workers, midwives in particular, gained confidence and skills in inserting intravenous cannulas for medicines and dextrose, as well as nasogastric tubes for feeding preterm babies expressed breast milk, and for conducting neonatal resuscitation. In terms of identifying and supporting champions, most facilities had specific staff that took up more responsibility and had more enthusiasm for the relatively novel area of newborn care, and used their time to mobilise and train others.

### Service delivery

Infrastructural improvements, particularly the creation of a KMC unit, transformed service delivery (Box 1). While at baseline there were no additional services available for small babies at the hospital or HCs, by the end of the study, 547 preterm babies had been cared for in a KMC unit. Of those admitted to the hospital's KMC unit, 85% were discharged alive, with less than 2% of admissions requiring referral to higher-level care. Three-quarters of mother–baby pairs completed KMC follow-up to ‘graduate’ from the service. A substantial proportion (13%) of women admitted to KMC self-discharged against medical advice. Attempts were made to follow-up these cases in the community, and where possible they were linked to a lower-level health facility for continued care. Other infrastructural adjustments, such as the partitioning of the paediatric ward to allocate space for newborns, resulted in neonates being seen as needing special care apart from maternity and older child services. Differences between public and private facilities were observed, with the practice of essential newborn care services slightly higher in public facilities (17).

Box 1 **Twins benefit from newly introduced kangaroo mother care (KMC) practice**

When KMC was first being introduced at the hospital, during a supervision visit, the mentorship team found a woman admitted to the maternity ward who had delivered premature twins by caesarean section 6 days prior. The babies were weak, hypothermic, and were being fed glucose water. Their mother had painful, engorged breasts and remained weak following surgery.

The supervision team counselled the health workers to admit the mother and babies to the KMC unit. The mother was supported to express breast milk while nasogastric tubes were passed for feeding the babies. The health workers were instructed on how to calculate feeds and do daily routine monitoring, including weighing. The grandmother and the mother of the twins were counselled on carrying the babies skin-to-skin while the mother regained strength and could take over the skin-to-skin care. The mother and twins were discharged after 3 weeks, and came back for weekly reviews.

Treatment protocols on admission, feeding, monitoring, and discharge were developed and posted on the walls of the KMC unit. A motivated midwife was identified to champion KMC amongst staff and mothers and to serve as a point person for the mentorship team. While this single-champion strategy was successful as a short-term solution, high turnover amongst staff means that a broader leadership or steering team for KMC is beneficial.

Supervision visits and learning opportunities were well utilised. Based on competencies observed during supervision visits, staffing adjustments were made by the district health team. Supervision visits lasted 3 days each, with half a day spent at each lower-level HC and 1 full day at the hospital. Exchange visits were provided for hospital staff at the national referral hospital and a general hospital in the capital city of Kampala for additional mentorship. Despite widespread national and local support for the mortality review process, this was not well taken up by facility staff and administration, citing competing demands and excessive workload. Fear of blame for specific failures in care might also have been a reason for discontinuation.

### Equipment and supplies

At the level IV HC and hospital, less than half of the essential equipment was available and/or functional at baseline, with level II and III HCs functioning better for supplies, with 70% being available. Following the initial procurement and distribution of supplies to health facilities, these levels were maintained and even increased in the level IV HC and hospital. Still, only 80% of level II and III HCs had the basic equipment necessary by the end of the implementation period. Due to increasing demand for services and despite support for procurement, stock-outs of essential medicines were common throughout the implementation period and remained below 40%. This was more pronounced in the level IV HC and hospital compared to the lower-level health facilities, both due to increasing demand and budgetary constraints of the ‘pull’ procurement system.

### Health information

The availability and quality of health information improved dramatically over the course of the implementation. Whereas at baseline there were very few indicators captured within the routine system for neonates, outcome data became readily available and were requested by administrators and district leaders through the HMIS. Partograph use and completeness, documentation of weight, feeding, and treatments received all increased. The completeness and accuracy of inpatient and outpatient register documentation improved along with individual patient charts, although these were dependent on continued availability of stationery after the initial seed stock was consumed.

### Leadership/governance and finance

Creating linkages among the health workers, community leaders, and district officials was an important part of ensuring continuity of care from the provider side. Health workers met with CHWs on a monthly basis at the health facility, and once a month they accompanied CHWs on home visits. Through community meetings, challenges at community level and health facility were discussed and raised with leadership, for example, the lack of housing available for health facility staff (Box 2). Managers were key drivers of all of the quality improvement processes employed. However, some of the major health systems bottlenecks (e.g. availability of finances for maintaining the supply of essential medicines) were outside of the control of local managers.

Box 2 **Ensuring availability of essential equipment and supplies: a health centre (HC) III case study**

According to national policy, level III HCs should be able to perform normal deliveries. However, at baseline, it was identified that one HC III had two midwives on its staff but was lacking all basic equipment, including a delivery bed, clean delivery kits, and a surface for resuscitation. Most of the women in the sub-county were delivering at home or at the home of a traditional birth attendant due to the long distance to the next nearest facility.

Upon initiation of UNEST, the HC III received an initial seed stock of supplies, equipment, and essential medicines. Through mentorship visits, the maternity ward was reorganised for efficiency. Staff received support for using equipment and documenting care. While the number of births in the facility steadily increased, the ability to provide around-the-clock services was constrained given the lack of accommodation for midwives at or near the facility. Upon discussion with sub-county leaders and the district health team, it was agreed that housing would be donated by the community and constructed and provided onsite.

## Discussion

The health systems strengthening efforts in this study reflect the limited material input possible within the existing district structure. The selected interventions were identified together with the Ministry of Health, district health team, and local experts, and were within the confines of the Uganda National Minimum Health Service Package. The number of deliveries with a trained, equipped birth attendant increased, with substantial improvements in the care provided to sick and preterm babies, particularly in public sector facilities. A strategy involving simple structural improvements, in-service training opportunities, effective team-based mentoring, and improved documentation has the potential to strengthen the capacity of the providers to care for vulnerable newborn babies. However, the shortage of essential drugs and supplies remains a major bottleneck in the system.

Simple partitioning within the delivery, postnatal and paediatric wards to provide a designated space for newborns likely contributed to improved awareness of newborn care in the community. Interviews with mothers and health providers demonstrated increased confidence in the services they were receiving and providing (14). The rate of self-discharge amongst women admitted to KMC possibly reflects the additional cost to families (e.g. meals, care for children at home) for inpatient care and also lack of exposure to KMC as a treatment regimen. Mechanisms to alleviate this burden and increase demand for KMC services should be explored. Given that KMC was only introduced at the hospital, there is an opportunity for step-down services to be rolled out to lower-level health facilities, and allowing services to be provided closer to home for many families.

Based on feedback following trainings, the in-service training was well accepted. The increase in provider knowledge 1 year after training reflects the importance of mentorship visits and support supervision. Increased frequency of *in situ* training is likely to further improve health worker skills, as demonstrated in Tanzania (18). Recent updates to the national nursing and midwifery training curriculum to cover aspects of newborn care, including KMC, will ensure that future staff have additional exposure to maternal and newborn complications as well as caring for sick and small babies. Ongoing support supervision and mentorship in addition to learning opportunities supported the retention of knowledge and skills of previously trained providers. The development of champions within the hospital and HCs was critical, particularly to support the newly introduced KMC service.

The equipment and medicines provided reflect only the most basic supplies necessary to provide maternal and newborn care. Yet, after the initial catalytic supply, the availability of medicines was erratic. While equipment levels were higher throughout the study period, there were also issues around maintenance and replacement. While administrative officials were encouraged to prioritise procurement, more effort is needed to improve budgeting and governance structures and to engage with the central medical stores to ensure that these are available. With an increasing volume of births in an area of high fertility, accurate planning for the quantity of necessary commodities is essential. Factors such as the availability of accommodation for service providers and the lack of ambulances and other emergency transport services were identified, but are largely beyond the ability of the study to address in the short term.

There are several limitations to this study. First, although data were compiled by study researchers, they were collected within routine systems. Improving data collection was one of the interventions targeted through supervision visits, which may present bias. In addition, the study did not assess the quality of care provided, nor were health worker skills observed and evaluated systematically. While health facility strengthening was introduced alongside the randomised community-based package, it is not likely that the increase in awareness of and demand for services was solely related to the community intervention, given that the catchment area for the facilities, particularly the hospital, is well beyond the implementation districts. The reasons for the increase in utilisation of services are multifactorial, but it is possible that the intervention contributed to this increase as well as to the overall quality of services provided to families.

Finally, given that there was little prior experience with maternal and newborn care services, the quality improvement interventions were introduced stepwise, which may have limited their effect, because there was a limited time period for the new services to take hold and be implemented together. An independent examination of mortality effect and impact on neonatal outcomes would be beneficial to supplement routine data and highlight potential gaps in measurement.

## Conclusion

Improving awareness of and demand for maternal and newborn care services at community level necessitates engagement with health facilities in order to ensure services are available and of sufficient quality. Even amongst higher-level health facilities, availability and quality of maternal and newborn care was low or non-existent. Basic health system strengthening was feasible in this low-resource setting, mostly within existing resources. Alignment with the Ugandan minimum health service package and the national standards for newborn health care demonstrates the potential for replication in other districts. Local leadership support, particularly engaging key champions and ensuring buy-in from frontline health providers, is required from the outset. However, addressing quality of care bottlenecks is a significant challenge and further innovative solutions are needed for resource constrained settings in order to save the lives of mothers and babies and help them thrive.

## References

[1] Lawn JE, Blencowe H, Darmstadt GL, Bhutta ZA (2013). Beyond newborn survival: the world you are born into determines your risk of disability-free survival. Pediatr Res.

[2] MoH (2005). Health sector strategic plan II 2005/06–2009/10.

[3] Kallander K, Hildenwall H, Waiswa P, Galiwango E, Peterson S, Pariyo G (2008). Delayed care seeking for fatal pneumonia in children aged under five years in Uganda: a case-series study. Bull World Health Organ.

[4] Waiswa P, Kallander K, Peterson S, Tomson G, Pariyo GW (2010). Using the three delays model to understand why newborn babies die in eastern Uganda. Trop Med Int Health.

[5] Mbonye AK, Asimwe JB, Kabarangira J, Nanda G, Orinda V (2007). Emergency obstetric care as the priority intervention to reduce maternal mortality in Uganda. Int J Gynaecol Obstet.

[6] Waiswa P, Kemigisa M, Kiguli J, Naikoba S, Pariyo GW, Peterson S (2008). Acceptability of evidence-based neonatal care practices in rural Uganda – implications for programming. BMC Pregnancy Childbirth.

[7] Kerber KJ, de Graft-Johnson JE, Bhutta ZA, Okong P, Starrs A, Lawn JE (2007). Continuum of care for maternal, newborn, and child health: from slogan to service delivery. Lancet.

[8] Okuonzi SA (2004). Learning from failed health reform in Uganda. BMJ.

[9] MoH (2008). Situation analysis of newborn health in Uganda. Current status and opportunities to improve care and survival.

[10] WHO (2007). Everybody's business – strengthening health systems to improve health outcomes: WHO's framework for action.

[11] Lundberg M, Amin S, Das J, Goldstein M (2008). Client satisfaction and the perceived quality of primary health care in Uganda. *Are You Being Served? New Tools for Measuring Service Delivery*.

[12] Atun R, de Jongh T, Secci F, Ohiri K, Adeyi O (2010). A systematic review of the evidence on integration of targeted health interventions into health systems. Health Policy Plan.

[13] Waiswa P, Peterson SS, Namazzi G, Ekirapa EK, Naikoba S, Byaruhanga R (2012). The Uganda Newborn Study (UNEST): an effectiveness study on improving newborn health and survival in rural Uganda through a community-based intervention linked to health facilities – study protocol for a cluster randomized controlled trial. Trials.

[14] Waiswa P, Pariyo G, Kallander K, Akuze J, Namazzi G, Ekirapa-Kiracho E (2014). Effect of the Uganda Newborn Study on care-seeking and care practices: a cluster-randomised controlled trial. Glob Health Action.

[15] Ministry of Health (2010). Newborn implementation framework: standards for newborn health care services.

[16] MoH (2010). Maternal and perinatal death review guidelines.

[17] Waiswa P, Akuze J, Peterson S, Kerber K, Tetui M, Forsberg BC (2014). Differences in essential newborn care at birth between private and public health facilities in eastern Uganda. Glob Health Action.

[18] Mduma E, Ersdal HL, Svensen E, Perlman J (2014). Low-dose high-frequency simulation training reduces early neonatal mortality. International Meeting on Simulation in Healthcare (IMSH).

# Sub article: Differences in essential newborn care at birth between private and public health facilities in eastern Uganda

**DOI:** 10.3402/gha.v8.24251

**Published:** 2015-03-31

**Authors:** Peter Waiswa, Joseph Akuze, Stefan Peterson, Kate Kerber, Moses Tetui, Birger C. Forsberg, Claudia Hanson

**Affiliations:** 1 Global Health, Department of Public Health Sciences, Karolinska Institutet, Stockholm, Sweden; 2 Department of Health Policy, Planning and Management, Makerere University School of Public Health, Kampala, Uganda; 3 Iganga-Mayuge Health Demographic Surveillance Site, Iganga-Mayuge, Uganda; 4 International Maternal and Child Health Unit, Department of Women's and Children's Health, Uppsala University, Uppsala, Sweden; 5 Saving Newborn Lives, Save the Children, Cape Town, South Africa

**Keywords:** newborn health, private health care, public health care, essential newborn care, Uganda

## Abstract

**Background:**

In Uganda and elsewhere, the private sector provides an increasing and significant proportion of maternal and child health services. However, little is known whether private care results in better quality services and improved outcomes compared to the public sector, especially regarding care at the time of birth.

**Objective:**

To describe the characteristics of care-seekers and assess newborn care practices and services received at public and private facilities in rural eastern Uganda.

**Design:**

Within a community-based maternal and newborn care intervention with health systems strengthening, we collected data from mothers with infants at baseline and endline using a structured questionnaire. Descriptive, bivariate, and multivariate data analysis comparing nine newborn care practices and three composite newborn care indicators among private and public health facilities was conducted.

**Results:**

The proportion of women giving birth at private facilities decreased from 25% at baseline to 17% at endline, whereas overall facility births increased. Private health facilities did not perform significantly better than public health facilities in terms of coverage of any essential newborn care interventions, and babies were more likely to receive thermal care practices in public facilities compared to private (68% compared to 60%, *p*=0.007). Babies born at public health facilities received an average of 7.0 essential newborn care interventions compared to 6.2 at private facilities (*p*<0.001). Women delivering in private facilities were more likely to have higher parity, lower socio-economic status, less education, to seek antenatal care later in pregnancy, and to have a normal delivery compared to women delivering in public facilities.

**Conclusions:**

In this setting, private health facilities serve a vulnerable population and provide access to service for those who might not otherwise have it. However, provision of essential newborn care practices was slightly lower in private compared to public facilities, calling for quality improvement in both private and public sector facilities, and a greater emphasis on tracking access to and quality of care in private sector facilities.

Newborn mortality (deaths within the first 28 days of life) remains unacceptably high in sub-Saharan Africa and in Uganda. Every year 2.9 million babies die during the neonatal period (1), with the majority of these deaths occurring in the first week of life. This is also the time of greatest risk for stillbirths and maternal deaths (2). In Uganda, out of 1.5 million births in 2012, 82,000 resulted in a mother or baby dying (3). There are many missed opportunities to improve care and increase the potential to save lives at and immediately after birth. Saving mothers and babies is rarely the result of a single, simple intervention, but a complex and comprehensive set of interlinked services and practices supported by health workers.

Skilled attendance at birth is considered a critically important platform to reduce the burden of maternal and newborn mortality worldwide (4, 5). If backed by a referral level providing comprehensive emergency obstetric care, uptake of skilled attendance will prevent by far the largest part of maternal and newborn mortality as well as many stillbirths (2). However, skilled attendance will only have the promised effect if the different components of it are implemented, and thus quality of care is assured (6). Although quality of care is a complex and multidimensional concept including safety, effectiveness, timeliness, efficiency, equity, and patient-centredness (7), measuring signal indicators may provide some insight into the coverage and quality of care overall (8).

Private for-profit providers, typically small privately owned clinics with a single proprietor, play a significant role in provision of outpatient health care and reproductive health, but involvement in maternity care is a relatively recent phenomenon in sub-Saharan Africa, where most women still deliver at home or choose the public health system (9). Still, in some countries such as Nigeria or Kenya, a larger proportion of deliveries are now taking place in private clinics and hospitals (10–13). One of the milestones of the newly launched Every Newborn Action Plan is coordinated support and effort amongst private sector providers of delivery services and newborn care (14).

Engagement of the private sector to increase accessibility to reproductive and child health care is much debated (15). Some studies have reported that greater participation of the private sector improved access to and equity in care (16, 17), whereas others indicated the opposite (18). Criticisms in regard to private maternity care include late referral to public health facilities in the case of obstetric emergencies, as private maternity facilities are not always equipped to provide 24-h emergency obstetric care services (19, 20). Where operative services are available, the fees charged for a caesarean section delivery might increase caesarean section rates, particularly where third-party fee-for-service reimbursement gives health providers an income from their services.

Families often seek out private health facilities as they perceive the quality of care as better overall (21–28), although a recent systematic review suggests that quality in both public and private provider groups is poor, with the private sector being better in terms of drug availability and aspects of responsiveness to client expectations (29). Results from an investigation into the use of private maternity services from a Nairobi informal settlement indicated that private care was less costly, closer to the home, and providers were more empathic (30, 31). In Nigeria, private maternity care was the preferred place of delivery because of the low quality of government facilities, particularly with respect to absence of staff, poor perceived quality, waiting times, and high costs (11).

Despite increasing prominence of the private sector as a provider of delivery and newborn care, there is a dearth of data on newborn care practices in these facilities. To the best of our knowledge, no information on the quality of delivery care of private providers is available from Uganda. As part of the Uganda Newborn Study (UNEST) (32), we engaged public as well as private for-profit and not-for-profit providers through sensitisation, training, and supervision around childbirth and newborn care. UNEST aimed at improving newborn survival through a community-based intervention using home visits by volunteers linked to health facilities. The intervention included a health system strengthening component and improving linkages between the community-based intervention and the health facilities. Here we present the determinants of use and the quality of public and private maternity care. Furthermore, we assessed the impact of facility strengthening on implementation of essential newborn care interventions among births that occurred in private and public health facilities in rural eastern Uganda. This is the sixth paper in a series on the UNEST results.

## Methods

### Study design and setting

The UNEST design and package has been described elsewhere (32–34). In brief, the study took place in the Iganga-Mayuge Health and Demographic Surveillance Site (HDSS) located in Iganga and Mayuge districts in the eastern region of Uganda, about 120 km east of the capital city of Kampala. The HDSS serves a population size of 70,000 people, at the time of the study, living in 65 villages, with women of reproductive age comprising 23%. The total fertility rate of the HDSS is 4.3. The population is served by 20 facilities including six private facilities (Fig. 1). The public hospital in Iganga is the only comprehensive emergency obstetric care facility. The public facilities charge no fees for services, although there are often informal costs requested of families. Typically, private facilities consisted of a small clinic with less than five staff who could provide essential care for common conditions. Private facilities are more accessible to the population and sometimes to rural areas than public facilities.

**Fig. 1:** Map of the UNEST study area.

Villages were randomised to intervention or control arms. Intervention villages had a community health worker who was trained to provide home visits during pregnancy and the first week after delivery, whereas comparison villages received the standard care as delivered by the facilities in the area. Health facility strengthening including training of health workers on essential maternal-newborn care skills and provision of medicine, basic equipment, and supplies was done in all health facilities with a reasonable client load (more than 15–20 per month) for delivery care, independent of ownership and management or whether the facility was located in the intervention or comparison area. Both public and private health facilities were supported by quarterly supervision as part of the health system strengthening. In addition, linkages between community and health facilities were strengthened.

### Data collection

A standardised tool was adapted and pretested for data collection. Data collectors were experienced HDSS field staff. The baseline census was done between March and August 2007. Women with infants aged 1–4 months (*n*=395) in the HDSS were interviewed through visits to all households (35). At endline census, done between August and November 2011, we interviewed all women of childbearing age who had had a live birth in the previous 12 months (*n*=1,761) (17, 36–38).

### Data analysis

All analyses used Stata software version 12.1. Univariate and bivariate analyses were used to describe background characteristics of women who delivered in a health facility. The chi-square test was used to compare the difference between the private and public facilities as place of delivery. A multiple logistical regression model was constructed to identify determinants of private facility births using all of the explanatory variables which were significant at bivariate analysis. We checked for multicollinearity between the independent variables, and only included non-collinear variables in the analysis. For this study the effect of treatment – overall and within subgroups – and covariates were reported using odds ratios (ORs).

Data on nine essential newborn care practices were collected. These interventions included wrapping the baby immediately after birth using a dry cloth, early skin-to-skin placement, delayed bath at least 6 h after delivery, clean instrument used to cut the umbilical cord, clean device used to tie or clamp the cord, placing nothing on the cord stump, breastfeeding within the first hour after birth; not giving the baby a bottle, and not giving any food or drink other than breast milk. Interventions were combined into composite indicators for thermal care, hygienic cord care, and optimal feeding practices. In addition we assessed how many women received more than one to all nine essential newborn care interventions.

**Fig. 2:** Coverage of babies receiving essential newborn care interventions. * χ^2^prob=0.007

Wealth quintiles were constructed using the Principal Component Analysis based on household assets as used by the Ugandan Bureau of Statistics, including number of sleeping rooms, type of floor material, type of roof material, wall material, type of bed, fuel used for cooking, source of light; and possession of a radio, a sewing machine, an electric flat iron, charcoal flat iron, a bed net, kerosene lamp, kerosene stove, car, tea table, refrigerator, television set, sound stereo, telephone, mattress, wheelbarrow, cell phone, and camera. These gave a Cronbach's alpha of 0.848. Principal component analysis was performed and the first principal component was scored to create an asset index that was used to group all households in the HDSS into wealth quintiles (35). Schooling was assessed using categories of completed education level.

## Results

### Background characteristics

The average age of the women who delivered in a health facility was 26 years, with no significant difference between private and public sector (Table 1). Nine of 10 mothers were married. Slightly more than half of all women had primary education as the highest level attained, and 9% had no education at all. More than one-third of women (38%) had given birth four or more times. Almost all women (99%) attended at least one antenatal care (ANC) visit and 49% attended four or more times. Less than one-fourth (23.6%) of women attended ANC in their first trimester. The rate of caesarean section was 4.4% overall.

**Table 1:** Background characteristics of respondents for endline census

	Total (all facility deliveries)	Private facilities	Public facilities	

Characteristics	N (%)	N (%)	N (%)	*p*
Maternal age (yrs)	*n*=1,358	*n*=299	*n*=1,059	
<19	96 (7.07)	16 (5.35)	80 (7.55)	0.1797
19–25	564 (41.53)	120 (40.13)	444 (41.93)	0.5770
26–30	357 (26.29)	79 (26.42)	278 (26.25)	0.9530
>30	341 (25.11)	84 (28.09)	257 (24.27)	0.1786
Marital status	*n*=1,369	*n*=306	*n*=1,063	
Not married	116 (8.47)	22 (7.19)	94 (8.84)	0.3610
Married	1,253 (91.53)	284 (92.81)	969 (91.16)	0.3610
Education	*n*=1,369	*n*=306	*n*=1,063	
No education	127 (9.28)	33 (10.78)	94 (8.84)	0.3026
Primary	783 (57.20)	198 (64.71)	585 (55.03)	0.0026*
Secondary	407 (29.73)	68 (22.22)	339 (31.89)	0.0011*
University	52 (3.80)	7 (2.29)	45 (4.23)	0.1176
Wealth quintile	*n*=1,036	*n*=240	*n*=796	
1 (Poorest)	154 (14.86)	46 (19.17)	108 (13.57)	0.0326*
2 (Poor)	219 (21.14)	52 (21.67)	167 (20.98)	0.8185
3 (Average)	258 (24.90)	63 (26.25)	195 (24.50)	0.5827
4 (Rich)	207 (19.98)	48 (20.00)	159 (19.97)	0.9919
5 (Richest)	198 (19.11)	31 (12.92)	167 (20.98)	0.0054*
Parity	*n*=1,369	*n*=306	*n*=1,063	
1	262 (19.14)	37 (12.09)	225 (21.17)	<0.001
2–4	585 (42.73)	129 (42.16)	456 (42.90)	0.8176
>4	522 (38.13)	140 (45.75)	382 (35.94)	0.0019*
Number of ANC visits	*n*=1,351	*n*=300	*n*=1,051	
1	46 (3.40)	15 (5.00)	31 (2.95)	0.0842
2–3	646 (47.82)	142 (47.33)	504 (47.95)	0.8496
>3	659 (48.78)	143 (47.67)	516 (49.10)	0.6621
Trimester of first ANC visit	*n*=1,363	*n*=303	*n*=1,060	
1	322 (23.62)	59 (19.47)	263 (24.81)	0.0536*
2	875 (64.20)	198 (65.35)	677 (63.87)	0.6356
3	166 (12.18)	46 (15.18)	120 (11.32)	0.0700
Mode of delivery	*n*=1,369	*n*=306	*n*=1,063	
Spontaneous	1,307 (95.47)	303 (99.02)	1,004 (94.45)	<0.001*
Caesarean	60 (4.38)	3 (0.98)	57 (5.36)	0.001*
Other	2 (0.15)	0 (0.00)	2 (0.19)	0.4454

### Determinants of births in private facilities

Although there was an overall increase in health facility births, from 69.6% at baseline to 77.8% at endline, there was a decrease in private sector deliveries, from 25.1 to 17.3% overall (Table 2). Of the 1,369 women who delivered in a health facility, 22% gave birth in the private sector. Compared to their counterparts who delivered in public health facilities, women delivering in private facilities were significantly more likely to have higher parity, lower socio-economic status, and less education, and were more likely to seek ANC later in pregnancy. They were also more likely to have a normal delivery, associated with the lack of operative capacity in the majority of the private facilities. There was no significant association between the time when women went into labour and the place that they delivered (results not shown).

**Table 2:** Place of delivery

	Baseline		Endline	
	
	*n*=395	%	*n*=1,761	%
Facility delivery	275	69.6	1,369	77.7
Public facility	176	44.6	1,062	60.3
Private facility	99	25.1	306	17.4
Delivered by traditional birth attendant	44	11.1	147	8.3
Delivered at home or elsewhere	110	27.8	245	13.9
Missing	10	2.5	0	0.0

According to the logistical regression analysis (Table 3) women who had two to four previous births compared to those with only one birth were almost twice as likely to deliver in a private health facility (OR 1.86, 95% CI 1.05–3.30). Women with more than four previous births were two times more likely to deliver in a private facility (OR 2.36; 95% confidence interval (CI) 1.34–4.16). In addition, delivery in private health facilities was less likely (although non-significantly so) for mothers of higher wealth quintiles (OR 0.58, CI 0.33–1.02). The odds of women who delivered in private facilities having a caesarean section was 80% lower than in those who delivered in public facilities (OR 0.20, 95% CI 0.48–0.86).

**Table 3:** Determinants of births in private health facilities

Variable	Univariate unadjusted		Multivariate unadjusted	
	
	OR	95% CI	OR	95% CI
Parity				
1	1		1	
2–4	1.72	1.15–2.56	1.72	1.06–2.81
>4	2.22	1.50–3.32	2.01	1.22–3.31
Wealth quintile				
1 (Poorest)	1			
2 (Poor)	0.73	0.46–1.16	0.75	0.46–1.21
3 (Average)	0.76	0.49–1.19	0.75	0.48–1.19
4 (Rich)	0.71	0.44–1.14	0.75	0.46–1.22
5 (Richest)	0.44	0.26–0.73	0.52	0.30–0.90
Education level				
No education	1			
Primary	0.96	0.63–1.48	1.08	0.67–1.76
Secondary or higher	0.56	0.35–0.89	0.93	0.53–1.66
Trimester of first ANC visit				
1	1			
2	1.30	0.94–1.80	1.31	0.89–1.92
3	1.71	1.10–2.66	1.77	1.07–2.95
Mode of delivery				
Normal	1			
Caesarean	0.17	0.05–0.56	0.20	0.48–0.84

### Coverage of essential newborn care practices by place of birth

Amongst all facility births, coverage of essential newborn care practices varied from a low of 58.6% of women practicing dry cord care to 94.8% use of a clean instrument to clamp or tie the umbilical cord. However, the composite indicators of babies receiving all basic essential interventions were much lower, range being 11.5% optimal feeding practices, 46.9% hygienic cord care, and 66.3% receiving thermal protection overall. With the exception of immediate breastfeeding, the coverage of individual essential newborn care practices was higher but not significantly different in public sector facilities compared to private facilities (Table 4). Similarly, the composite essential newborn care indicators of optimal feeding practices, hygienic cord care, and thermal protection were all higher in public facilities, with thermal care practices significantly higher at 68.1% coverage in public sector facilities compared to 59.8% in private facilities (Fig. 2).

**Table 4:** Reported neonatal care practices by place of delivery

	Public health facilities	Private health facilities	
	
Practices	*N* (%)	*N* (%)	*p*
Clean instrument used to cut the cord	*n*=1,063	*n*=306	
	883 (83.07)	252 (82.35)	0.7681
Clean instrument used to tie or clamp the cord	*n*=1,063	*n*=306	
	1,014 (95.39)	285 (93.14)	0.1153
Dry cord care	*n*=1,062	*n*=304	
	631 (59.42)	170 (55.92)	0.2733
Breastfed within first hour	*n*=1,063	*n*=306	
	734 (69.05)	218 (71.24)	0.4633
Baby fed by breast only (no bottle)	*n*=1,063	*n*=306	
	1,026 (96.52)	300 (98.04)	0.1791
Exclusive breastfeeding in the first month	*n*=1,063	*n*=305	
	862 (81.09)	247 (80.98)	0.9655
Baby wrapped after delivery with dry cloth	*n*=1,063	*n*=306	
	1,060 (99.72)	304 (99.35)	0.3428
Baby placed skin-to-skin	*n*=1,063	*n*=306	
	821 (77.23)	218 (71.24)	0.0309*
First bath delayed >6 h	*n*=1,055	*n*=302	
	920 (87.20)	255 (84.44)	0.2146
Hygienic cord care	*n*=1,063	*n*=306	
Clean instrument used to cut cord; clean instrument used to tie cord; nothing placed on cord	502(47.22)	140(45.75)	0.6498
Thermal protection	*n*=1,063	*n*=306	
Baby wrapped after delivery with dry cloth; placed skin-to-skin; bath delayed >6 h	724 (68.11)	183 (59.80)	0.0067*
Optimal feeding practices	*n*=1,063	*n*=306	
Baby breastfed within first hour; no bottle used; exclusive breastfeeding for the first month	128 (12.04)	30 (9.80)	0.2798

Babies born in public health facilities were more likely to receive more individual newborn care practices compared to their private health facility counterparts. Whereas 42.8% of babies born in public facilities received at least eight essential newborn care practices, only 27.5% in private facilities received the same number. Nearly all (98%) babies born in public health facilities received at least three practices, compared to 95% amongst those in private health facilities (Table 5).

**Table 5:** Number and distribution of essential newborn care interventions by place of delivery (%)

	Number of newborn care interventions received										

	9	8	7	6	5	4	3	2	1	0	Mean (SD)
Private facility	8.5	27.4	46.8	68.8	81.0	89.5	95.1	98.2	99.5	100.0	6.16 (1.92)
Public facility	16.4	42.8	67.8	82.9	92.4	96.6	98.4	99.5	99.8	100.0	7.04 (1.59)
*p*											<0.001*

Interventions include: Wrapping the baby using a dry cloth; early skin-to-skin placement; delayed bath at least 6 h after delivery; clean instrument used to cut the umbilical cord; clean device used to tie or clamp the cord; placing nothing on the cord stump; breastfeeding within the first hour after birth; no use of bottle; not giving any food or drink other than breast milk for the first month.

## Discussion

To our knowledge this is the first study to compare the difference in newborn care practices between private and public health facilities in Uganda. We found that there is little difference in newborn care practices in private and public facilities. Private facilities are more likely to be accessed by the poorest families and at-risk women. After health system strengthening, including health worker training, provision of essential supplies, and supervision in both public and private sectors, there was an overall increase in health facility births. During the same period we observed a decline in private sector deliveries.

The increase in facility deliveries in public facilities suggests that the health system strengthening activities had a positive impact on utilisation. Although we cannot make a causal interference as the study is based on two subsequent cross-sectional surveys without any comparison area, the temporal relationship gives some indication that such an association might exist. No other intervention which might confound the association was ongoing in the study area. As the place of delivery is likely to be an indicator which is easily remembered, we do not think that the difference in recall period (4 months at baseline and 12 months at endline) may bias the results; in most surveys recall periods of one year or more are used (39).

Thus, we think that it is likely that the combined supply- and demand-side interventions in UNEST resulted in perceived or real improvements in care, including public sector health workers being more receptive and responsive to clients. Possibly the intervention could have influenced perceived quality of and access to public sector services.

These findings have public health implications for Uganda, and also for other low- and middle-income countries looking to strengthen care at the time of birth.

In this setting, women who delivered at private health facilities had a higher-risk birth profile compared to women who sought care at public facilities. Women who gave birth in private facilities were associated with lower socio-economic status, higher parity, lower education, and were more likely to attend ANC later in pregnancy. Private facility-based delivery care in sub-Saharan Africa is typically associated with the urban rich and more educated segment of the population. However, in our experience, this is only true for the more advanced private health units, not for the majority of private units in rural areas, that are often small and designed to serve the poor.

The use of a private health facility, however, bears the risks of potentially catastrophic costs associated with obstetric care (40, 41), making the pattern of private sector care seeking amongst poorer families potentially harmful. However, a similar pattern as observed in our study in rural Uganda has also been described in Nairobi's informal settlements (31). A plausible explanation could lie in the fact that public health facilities are more distant whereas private care providers are strategically placed to maximise access and to fill a demand gap, especially in more rural areas. In addition, informal payments – which are common in the public sector in Uganda – might have led families to make a rational choice to save on transport costs but pay more for the delivery care (42, 43).

Private health care was not found to be synonymous with better capacity and quality. The private health facilities in the study setting had less capacity in terms of infrastructure, staffing, equipment, and medicines (32). The lack of emergency obstetric services at private health facilities and referral delays pose a real risk to the survival and health of mothers and babies. Through UNEST, private as well as government health facilities were targeted for health system strengthening, with the knowledge that the private ones are often overlooked by development partners and districts in capacity-building efforts such as dissemination of service guidelines; in-service training; provision of equipment, drugs, and supplies; and supervision and mentorship. Although public health facilities performed on par with private health facilities across almost all newborn care practices, coverage overall was not optimal, especially in the case of early and exclusive breastfeeding. These essential newborn care practices are inexpensive and require little if any technology and commodities. The low coverage represents a missed opportunity for all births, regardless of place of delivery.

More information is needed to understand the patterns of care seeking in the public and private sector. There are few disaggregated data available in terms of utilisation as the main source of population-based data, the Demographic and Health Surveys, combines private for-profit and private not-for-profit facilities together, obscuring this potentially important distinction in healthcare provision (9).

This study has some limitations. Newborn care practices were assessed by asking the mother about the care she had received or provided to her newborn. Such responses are subject to recall bias, as some women might not remember interventions implemented during or immediately after birth, particularly for complicated deliveries. However, it can be assumed that such recall bias is similar in women receiving care from a public or private provider. Restricting survey respondents to women who had live births may limit understanding of the potentially different profile of care received by women who experienced stillbirths or early pregnancy loss. However, it was not considered appropriate or feasible to interview such women due to the sensitive nature of their experience. The study did not assess the capacity of private or government health facilities to provide essential newborn care, such as the availability of staff, training received on newborn care, or availability of equipment and supplies. Observation studies would have provided more accurate data, but they consume much time and resources.

## Conclusion

As countries increase attention on improving coverage and quality of maternal and newborn care, and in the context of the post-Millennium Development Goal agenda focusing on universal health care, it is important to also consider the role of private sector providers, especially those in rural and urban poor areas which serve under-reached, vulnerable populations. Financial, geographic, and sociocultural barriers to accessing public sector care should be further explored. As for the public facilities, the private sector also requires accountability mechanisms and capacity-building activities, including training and supervision support and guidance on evidence-based best practices for newborn care.

## References

[1] UN Inter-agency Group for Child Mortality Estimations (2013). Levels and trends in child mortality. Estimates developed by the UN Inter-agency Group for Child Mortality Estimation.

[2] Pattinson R, Kerber K, Buchmann E, Friberg IK, Belizan M, Lansky S (2011). Stillbirths: how can health systems deliver for mothers and babies?. Lancet.

[3] Mbonye AK, Sentongo M, Mukasa GK, Byaruhanga R, Sentumbwe-Mugisa O, Waiswa P (2012). Newborn survival in Uganda: a decade of change and future implications. Health Policy Plan.

[4] Campbell O, Graham W (2006). The Lancet Maternal Survival Series steering group. Strategies for reducing maternal mortality: getting on with what works. Lancet.

[5] WHO (1999). Reduction of maternal mortality. A joint WHO/UNFPA/UNICEF/World Bank statement.

[6] Graham WJ, Varghese B (2011). Quality, quality. quality: gaps in the continuum of care. Lancet.

[7] Institute of Medicine Crossing the chasm: the IOM Health Care Quality Initiative. http://www.iom.edu/Global/News%20Announcements/Crossing-the-Quality-Chasm-The-IOM-Health-Care-Quality-Initiative.aspx.

[8] Gabrysch S, Civitelli G, Edmond KM, Mathai M, Ali M, Bhutta ZA (2012). New signal functions to measure the ability of health facilities to provide routine and emergency newborn care. PLoS Med.

[9] Nguyen H, Snider J, Ravishankar N, Magvanjav O (2011). Assessing public and private sector contributions in reproductive health financing and utilization for six sub-Saharan African countries. Reprod Health Matters.

[10] Okonkwo J, Ibeh C (2006). The contribution of privately owned hospitals in the provision of essential obstetric care in Nigeria. Niger J Clin Pract.

[11] Osubor K, Fatusi A, Chiwuzie J (2006). Maternal health-seeking behavior and associated factors in a rural Nigerian community. Matern Child Health J.

[12] Limwattananon S, Tangcharoensathien V, Sirilak S (2011). Trends and inequities in where women delivered their babies in 25 low-income countries: evidence from demographic and health surveys. Reprod Health Matters.

[13] Peters DH, Mirchandani GG, Hansen PM (2004). Strategies for engaging the private sector in sexual and reproductive health: how effective are they?. Health Policy Plann.

[14] Mason E, McDougall L, Lawn JE, Gupta A, Claeson M, Pillay Y (2014). From evidence to action to deliver a healthy start for the next generation. Lancet.

[15] Berer M (2011). Privatisation in health systems in developing countries: what's in a name?. Reprod Health Matters.

[16] Yoong J, Burger N, Spreng C, Sood N (2010). Private sector participation and health system performance in sub-Saharan Africa. PLoS One.

[17] Brugha R, Pritze-Aliassime S (2003). Promoting safe motherhood through the private sector in low- and middle-income countries. Bull World Health Organ.

[18] Hotchkiss D, Godha D, Do M (2011). Effect of an expansion in private sector provision of contraceptive supplies on horizontal inequity in modern contraceptive use: evidence from Africa and Asia. Int J Equity Health.

[19] Almerie Y, Almerie M, Matar H, Shahrour Y, Al Chamat A, Abdulsalam A (2010). Obstetric near-miss and maternal mortality in maternity university hospital, Damascus, Syria: a retrospective study. BMC Pregnancy Childbirth.

[20] Adisasmita A, Deviany P, Nandiaty F, Stanton C, Ronsmans C (2008). Obstetric near miss and deaths in public and private hospitals in Indonesia. BMC Pregnancy Childbirth.

[21] Kallander K, Kadobera D, Williams TN, Nielsen RT, Yevoo L, Mutebi A (2011). Social autopsy: INDEPTH network experiences of utility, process, practices, and challenges in investigating causes and contributors to mortality. Popul Health Metr.

[22] Waiswa P, Kallander K, Peterson S, Tomson G, Pariyo GW (2010). Using the three delays model to understand why newborn babies die in eastern Uganda. Trop Med Int Health.

[23] Kallander K, Hildenwall H, Waiswa P, Galiwango E, Peterson S, Pariyo G (2008). Delayed care seeking for fatal pneumonia in children aged under five years in Uganda: a case-series study. Bull World Health Organ.

[24] Kalyango JN, Alfven T, Peterson S, Mugenyi K, Karamagi C, Rutebemberwa E (2013). Integrated community case management of malaria and pneumonia increases prompt and appropriate treatment for pneumonia symptoms in children under five years in eastern Uganda. Malar J.

[25] Kalyango JN, Rutebemberwa E, Karamagi C, Mworozi E, Ssali S, Alfven T (2013). High adherence to antimalarials and antibiotics under integrated community case management of illness in children less than five years in eastern Uganda. PLoS One.

[26] Rutebemberwa E, Nsabagasani X, Pariyo G, Tomson G, Peterson S, Kallander K (2009). Use of drugs, perceived drug efficacy and preferred providers for febrile children: implications for home management of fever. Malar J.

[27] Rutebemberwa E, Pariyo G, Peterson S, Tomson G, Kallander K (2009). Utilization of public or private health care providers by febrile children after user fee removal in Uganda. Malar J.

[28] Rutebemberwa E, Kallander K, Tomson G, Peterson S, Pariyo G (2009). Determinants of delay in care-seeking for febrile children in eastern Uganda. Trop Med Int Health.

[29] Berendes S, Heywood P, Oliver S, Garner P (2011). Quality of private and public ambulatory health care in low and middle income countries: systematic review of comparative studies. PLoS Med.

[30] Bazant ES, Koenig MA (2009). Women's satisfaction with delivery care in Nairobi's informal settlements. Int J Qual Health Care.

[31] Bazant ES, Koenig MA, Fotso J-C, Mills S (2009). Women's use of private and government health facilities for childbirth in Nairobi's informal settlements. Stud Fam Plann.

[32] Waiswa P, Pariyo G, Kallander K, Akuze J, Namazzi G, Ekirapa-Kiracho E (2014). Effect of the Uganda Newborn Study on care-seeking and care practices: a cluster-randomised controlled trial. Glob Health Action.

[33] Waiswa P, Peterson SS, Namazzi G, Ekirapa EK, Naikoba S, Byaruhanga R (2012). The Uganda Newborn Study (UNEST): an effectiveness study on improving newborn health and survival in rural Uganda through a community-based intervention linked to health facilities – study protocol for a cluster randomized controlled trial. Trials.

[34] Waiswa P, Namazzi G, Kerber K, Peterson S (2014). Designing for action: adapting and implementing a community-based newborn care package to affect national change in Uganda. Glob Health Action.

[35] Waiswa P, Peterson S, Tomson G, Pariyo GW (2010). Poor newborn care practices – a population based survey in eastern Uganda. BMC Pregnancy Childbirth.

[36] Chanrachakul B, Herabutya Y, Udomsubpayakul U (2000). Epidemic of cesarean section at the general, private and university hospitals in Thailand. J Obstet Gynaecol Res.

[37] Barros FC, Victora CE, Vaughan JP, Huttly SRA (1991). Epidemic of caesarean sections in Brazil. Lancet.

[38] Price M, Broomberg J (1990). The impact of the fee-for-service reimbursement system on the utilisation of health services. Part III. A comparison of caesarean section rates in white nulliparous women in the private and public sectors. S Afr Med J.

[39] Uganda Bureau of Statistics, MEASURE, ICF International (2012). Uganda demographic and health survey 2011.

[40] Arsenault C, Fournier P, Philibert A, Sissoko K, Coulibaly A, Tourigny C (2013). Emergency obstetric care in Mali: catastrophic spending and its impoverishing effects on households. Bull World Health Organ.

[41] Su TT, Kouyate B, Flessa S (2006). Catastrophic household expenditure for health care in a low-income society: a study from Nouna district, Burkina Faso. Bull World Health Organ.

[42] Xu K, Evans DB, Kadama P, Nabyonga J, Ogwal PO, Nabukhonzo P (2006). Understanding the impact of eliminating user fees: utilization and catastrophic health expenditures in Uganda. Soc Sci Med.

[43] McPake B, Asiimwe D, Mwesigye F, Ofumbi M, Ortenblad L, Streefland P (1999). Informal economic activities of public health workers in Uganda: implications for quality and accessibility of care. Soc Sci Med.

# Sub article: Weeping in silence: community experiences of stillbirths in rural eastern Uganda

**DOI:** 10.3402/gha.v8.24011

**Published:** 2015-03-31

**Authors:** Juliet Kiguli, Sarah Namusoko, Kate Kerber, Stefan Peterson, Peter Waiswa

**Affiliations:** 1 Department of Health Policy, Planning and Management, School of Public Health, Makerere University, Kampala, Uganda; 2 Saving Newborn Lives program, Save the Children, Cape Town, South Africa; 3 Global Health, Department of Public Health Sciences, Karolinska Institutet, Stockholm, Sweden; 4 International Maternal and Child Health Unit, Department of Women's and Children's Health, Uppsala University, Uppsala, Sweden

**Keywords:** stillbirth, pregnancy loss, Uganda, maternal health, postpartum depression, traditional birth attendants

## Abstract

**Background:**

Stillbirths do not register amongst national or global public health priorities, despite large numbers and known solutions. Although not accounted in statistics – these deaths count for families. Part of this disconnect is that very little is known about the lived experiences and perceptions of those experiencing this neglected problem.

**Objective:**

This study aimed to explore local definitions and perceived causes of stillbirths as well as coping mechanisms used by families affected by stillbirth in rural eastern Uganda.

**Design:**

A total of 29 in-depth interviews were conducted with women who had a stillbirth (14), men whose wives experienced a stillbirth (6), grandmothers (4), grandfathers (1), and traditional birth attendants (TBAs) (4). Participants were purposively recruited from the hospital maternity ward register, with additional recruitment done through community leaders and other participants. Data were analysed using content analysis.

**Results:**

Women and families affected by stillbirth report pregnancy loss as a common occurrence. Definitions and causes of stillbirth included the biomedical, societal, and spiritual. Disclosure of stillbirth varies with women who experience consecutive or multiple losses, subject to potential exclusion from the community and even the family. Methods for coping with stillbirth were varied and personal. Ritual burial practices were common, yet silent and mainly left to women, as opposed to public mourning for older children. There were no formal health system mechanisms to support or care for families affected by stillbirths.

**Conclusion:**

In a setting with strong collective ties, stillbirths are a burden borne by the affected family, and often just by the mother, rather than the community as a whole. Strategies are needed to address preventable stillbirths as well as to follow up with supportive services for those affected.

Whereas progress in maternal and child mortality rates is tracked in the Millennium Development Goals, stillbirths have not received similar attention (1, 2). This is despite the fact that each year an estimated 2.6 million stillbirths occur globally (3), in addition to the 2.9 million newborn deaths (4) and 289,000 maternal deaths (5).

Of the stillbirths, 1.2 million occur during labour, with the causes and solutions linked to care for mothers and newborns. Maternal conditions associated with stillbirth in low- and middle-income settings include hypertension, diabetes, maternal infection (e.g. syphilis, malaria, HIV), maternal undernutrition, obesity, and smoking (6). An estimated 40,000 stillbirths occur each year in Uganda, making it the country with the 10th highest number of stillbirths in the world. However, stillbirths are often unreported in official statistics and are invisible to health policy makers. Even in high-income countries, there are gaps in recognising the value of a dead baby, thereby dismissing the grief of the family (7). These deaths are not invisible to families – but social stigma around stillbirths and a lack of awareness about both the causes and potential solutions contribute to the silence (7).

During the design phase of Uganda Newborn Study (UNEST) in rural eastern Uganda, which aimed at strengthening health systems in order to reduce neonatal deaths, high rates of stillbirths were observed in the study area with few supporting services (8, 9). We therefore set out to explore community knowledge, attitudes, and practices, and coping of women and families affected by stillbirths, with the aim of this contributing towards designing effective preventive and care programmes. This paper is the seventh in a series on the impact of and findings from UNEST.

## Methods

### Setting

This study was part of the formative research for UNEST, a community-based newborn trial whose details have been published elsewhere (9, 10). The study was conducted in Iganga and Mayuge districts located in rural eastern Uganda, about 120 km east of the city of Kampala. The Basoga people, the predominant ethnic group in the area, make up about 10% of the population of Uganda, but their practices are similar to those of other Bantu ethnic groups in Uganda which comprise the majority. Eighty percent of the population are peasants and live on subsistence farming and retail trading which brings in an income of less than US$1 a day. TBAs are significant actors in the provision of care during pregnancy in the district, but the majority of births take place in health facilities. The main referral hospital delivers approximately 3,500 babies each year, of which 8% are stillbirths.

### Participants and data collection

This was a qualitative study employing in-depth interviews (IDIs) and observation in order to understand the context, lived realities, and interaction with the community in relation to stillbirths (11). There were a total of 29 study participants, including 7 men and 22 women (Table 1). The ages of the 14 women who delivered stillbirths ranged from 18 to 45 years with a mean age of 25 years. The parity range was 1–12 pregnancies. All study participants were of Basoga ethnicity. To recruit participants for the IDIs, we used the hospital maternity ward register to identify mothers of stillborn babies who had either a complete contact address for residential location and/or telephone numbers. Using the register information, we went to communities where these mothers came from. Additional recruitment was done through village community leaders and information gathered from interviewees.

**Table 1:** Number and type of IDI participants

Participants	*N*=29
Women who had at least one stillbirth	14
Men whose wives had at least one stillbirth	6
Grandmothers	4
Grandfathers	1
Traditional birth attendants	4

Interview guides were developed and pre-tested outside the study communities. IDIs were conducted in the local language of Lusoga with a note-taker present, and a Lusoga-speaking medical doctor supervised the research. Both the interviewer and note-taker were degree holders with over 5 years of experience in qualitative interview techniques. All of the interviews were conducted in Lusoga, tape-recorded, and later transcribed into English by the interviewer and the note-taker.

When interviewing participants, the interviewers used an exploratory technique and would probe to clearly understand and broaden the knowledge acquired. Interviewers used observation to document participants’ expressions and behaviours as they narrated their stories. Where relevant and feasible, participants went with interviewers to the place where the baby was buried to add context to the experience and enhance understanding. We explored what participants knew theoretically about stillbirths as well as their personal experiences of loss.

### Data analysis

Following transcription and translation into English, data were systematically coded and analysed manually by content analysis. The first three authors rigorously read the scripts independently and came up with codes, following what Granheim and Lundman refer to as condensation to unit analysis, content area (a process of shortening while still preserving the core), and meaning units (12). Thereafter, they met to compare and agree on the codes. Themes were identified and organised into meaningful categories, with frequencies and differences in responses by respondent type noted. Expressive and illuminating responses are presented as verbatim quotes.

Ethical approval was provided by the institutional review board of the Makerere University School of Public Health and Uganda National Council of Science and Technology. Given the emotional sensitivity around the topic, interviews took place no sooner than 6 weeks after the stillbirth event, and we used experienced research assistants from the same cultural group. Verbal consent was obtained from study participants given the low literacy rates.

## Results

The results of the IDIs reflect perceptions of the burden, definition, and causes of stillbirths as well as the coping mechanisms used by families to overcome their loss. Table 2 summarises the categories developed from broad themes of knowledge arising from the interviews.

**Table 2:** Community awareness, causes, perceptions, and beliefs about stillbirths

Condensed meaning unit	Code	Category	Theme
The dead baby is referred to as *ekintu*	A stillbirth is a thing, not fully human	How are stillbirths described	Definition of stillbirth
The baby is without life, does not breathe/no breadth of life	A stillbirth shows no sign of life	How stillbirths are understood medically	
The baby has no movements inside the mother's womb			
The baby has a soft head	Physical descriptions of stillbirths		
The baby has a long head		
The baby was disfigured/not well formed		
The baby comes out without the skin due to infection by the mother		
Maternal infections and diseases such as syphilis and malaria	Maternal infection	Perceived medical causes	Causes of stillbirth are varied
Baby is killed in the womb and syphilis pierces the baby			
There is *denyi*, much heat in the womb, and the uterus cannot hold			
Baby is pierced by syphilis, especially when syphilis is in advanced stage			
Tangled umbilical cord	Problems *in utero*		
Narrow birth canal			
Weak uterus			
Too young/women's bodies are not yet ready to hold a pregnancy	Age of mother		
Too old/some mothers feel tired after many births			
Sex during the pregnancy makes the baby come out before the time for birth	Activity while pregnant		
Falling while pregnant			
Heavy workload			
Intention to abort	Induced abortion		
Being poor and not able to afford to go for care	Poverty	Perceived societal causes	
Being kicked in the stomach	Domestic violence		
Bewitching by co-wife	Witchcraft	Spiritual causes	
Curses			
Attend antenatal care and go to hospital early	Changes in care-seeking activities	Solutions exist	
Attend a family planning clinic and space children			
Saving money; having good financial status	Changes at and around home		
Not doing heavy work and carrying heavy things			
Avoiding the use of certain herbs			
Using herbs such as *mubwa* to drink and others to rub on the stomach			
Witchcraft to block curses	Spiritual activities		
Religion and seeing a diviner			
Avoid telling other people of the pregnancy to as they may bewitch you			

### Perceived magnitude of the burden

Some participants reported that stillbirths were a common occurrence. Mothers who had experienced a stillbirth were likely to know about other women in their family or community who were similarly affected. However, it was thought that community members who were not affected by a stillbirth in their immediate family might not be aware of specific cases in the village:

We have so far had three stillbirths in this village and mine is the fourth. (Mother of a stillborn baby)

Since it is ‘*empuna*’ [*a thing*] which has not cried, they sometimes just announce to the immediate neighbours only. The rest of the village doesn't get to know about this curse. (TBA)

### Defining ‘stillbirth’

A stillbirth was locally referred to as *empuna* or *ekintu*, meaning ‘a thing’. Several definitions were used to describe stillbirths. The majority of women interviewed defined a stillbirth as a baby born dead, with no breath of life and no movement. Others described physical characteristics, such as a deformed baby with skin peeled off. A few could not define a stillbirth, even some of the women who had experienced it directly:

What we know is that a baby comes out dead, doesn't cry, and doesn't breathe. That shows us that the baby is a stillbirth. (Father of a stillborn baby)

The head was long, as if they had added another head, it was so soft and the baby was disfigured and not well formed. (Mother of a stillborn baby)

The difference between antepartum, or macerated stillbirths, and intrapartum, or fresh stillbirths, appeared to be important. Women were said to ‘weep in silence’, particularly if their baby appeared macerated or deformed. In these cases, few members of the family would be informed. Women who described this silence longed to speak about their experiences, but there was more stigma attached to these deaths than any other. The fathers of stillbirths reportedly did not cry, but expressed sadness for themselves and their wife.

### Medical and underlying causes of stillbirth

Causes of stillbirth were described firstly mainly in biomedical terms. These included maternal infections such as syphilis and malaria during pregnancy. Although syphilis was the most commonly mentioned condition, other reported physical causes of stillbirths included *denyi* (meaning there was ‘heat in the womb’), the baby becoming tired, the mother having a narrow birth canal, tying of the cord around the baby (which led to strangling of the baby) and having a ‘weak uterus’ which could not support the growth of the foetus. Particularly with maternal infection, like syphilis, the burden and responsibility seemed to fall heavily on the mother:

We know this disease [*syphilis*] can kill the baby in the womb. What I know is that they die before coming out, when syphilis is at its advanced stage. It can even come out as a miscarriage and at times the babies come out with spots on their bodies. At the end, it also causes too much heat in the body. The babies come out when already dead because of heat. (Father of a stillborn baby)

This is the third time. The first stillbirth happened at seven months, then another one followed at the eighth month at X hospital. On this one, I travelled up to until when I delivered. Everywhere I went, they told me it was syphilis. (Mother of three stillborn babies)

*Ekintu* does not make any movements when inside the uterus and … comes out without a skin due to the infection by the mother as this is transmitted to the baby. (Grandmother of a stillborn baby)

Age, body shape, and marital status were also identified as playing a role. TBAs mentioned that young mothers tended to have stillbirths as their bodies were not fully ready and prepared for child-bearing, while others, like mothers-in-law, mentioned that old mothers also lost children due to many children and having them one year after another. Some respondents noted that having sexual intercourse during pregnancy could result in a stillbirth. Several other causes were mentioned with links to societal factors and poverty. These included domestic violence, being poor and not being able to afford care, and stress from overwork: ‘A pregnant woman loses her baby if the husband kicks her in the stomach. For the husband comes home drunk and asks for his food and she delays to bring it over’ (TBA).

It was reported that if women had intentions to abort the baby, this could also result in a stillbirth. Witchcraft was commonly emphasised by mothers, especially in relation to their co-wives in a polygamous household. Some grandmothers said they would encourage their sons to marry new young women: ‘Sometimes, when one is pregnant, she does not tell other people for fear of losing the baby. One is afraid that someone may be jealous, for if you have a co-wife she will bewitch you’ (mother of a stillborn baby).

When asked about what contributes to babies dying before birth, participants mentioned delays in accessing health services. Financial barriers and lack of knowledge about when to seek care were cited as the source of the majority of delays. Another factor was the negative attitude of health workers, who were described as rude, proud, negligent, and vulgar. The midwives were quoted as often telling mothers in labour: ‘Who told you to become pregnant? Do not disturb us.’ Some young midwives were also said to abuse mothers, especially if the mother never attended antenatal care or if she had many previous pregnancies:

The health worker was only one and mothers were many, yet some health workers had gone to rest. By the time the health worker came from where she had gone to deliver another baby, she reached me when I had finished delivering a dead baby. Even the afterbirth had come out. (Mother of a stillborn baby)

Maybe if I had gone to the hospital on Friday, maybe they would have found out the baby's poor condition and tried to save it. But there was no solution. I would think the cause was the baby being tired because I delayed. (Mother of a stillborn baby)

Participants advanced several ways in which stillbirths could have been prevented, such as attending antenatal care early, saving money for emergencies, practising family planning, and avoiding a heavy workload. Divine intervention, protective witchcraft and the use of herbs during pregnancy were also regarded as important measures: ‘If I had known about my co-wife's witchcraft, then I would have consulted witchdoctors and saved my baby. My husband would have told you more of this story if he was around’ (mother of a stillborn baby).

### Effects of stillbirths on mothers, fathers, and families

The reported effects of experiencing a stillbirth on mothers and families and ways of coping are summarised in Table 3. Mothers who had a stillbirth said they were grieved because ‘every woman's happiness is giving birth’. Motherhood, and to a lesser degree fatherhood, confers societal value and is considered to be a respectful position in society. Participants noted that a stillbirth steals happiness from a family and may cause social disintegration or separation, even if followed by a live birth. Women who had multiple or consecutive stillbirths revealed that they felt ridiculed by their in-laws and community.

**Table 3:** Effect of a stillbirth on mothers and fathers and methods of coping

Participant	Response
Affected women	Mothers felt profound grief which increased with more than one stillbirth or pregnancy loss Women with consecutive stillbirths were considered cursed and lost the respect of their families and the community If it was known widely that a woman had a stillbirth, her worth may be questioned and her contributions in village meetings may not be taken seriously The woman is condemned and isolated, especially by in-laws, who encourage their son to remarry
Affected men	Experienced grief but did not cry; unlikely to mourn publicly Supportive to wives, especially in the case of a single stillbirth Blamed wife in the case of multiple stillbirths, although one man who had experienced multiple losses reported supporting his wife and not giving in to family pressure to remarry
Coping mechanisms	Support from the immediate family Older women in the extended family Trying to conceive again Counselling from sympathetic health workers Religion, belief in God

Men reported that they felt the loss strongly but reacted differently to women. Their reaction also differed between single and multiple stillbirth experiences. If consecutive stillbirths occurred, husbands were reported to reject and separate from their wives. The situation was worsened by pressure from the husband's family, who would encourage their son to leave his wife:

The men feel [*the pain of one stillbirth*], maybe [*the wife*] has some demons at her place of birth; two, maybe she is a woman with bad luck …. It can cause divorce, especially if the mother has had stillbirths twice yes … it can cause breakage of marriage and also anxiety. (Father of a stillborn baby)

However, dissolution of marriage following multiple stillbirths was not inevitable. Pressure from the community and family was further determined by the kind of relationship shared between husband and wife. It was argued that if the relationship was very good, it would not matter whether a woman had more than one stillbirth or whether the man's family had pressed him to get a divorce.

It was revealed that some family members were supportive to the mother while others would advocate for separation, in most cases regarding the woman as either a curse or a detraction to the man. Out of the 14 women who delivered stillbirths, half reported that family members were supportive; for the other half, their in-laws and co-wives were discouraging. In one report the condemnation extended to in-laws, who said the bereaved mother was filling the clan land with small graves:

Now my co-wife just tells me that ‘this time if you get pregnant again … You will be a woman.’ Otherwise she will say ‘Let her stop giving birth, the woman keeps burying every other time, she puts the family at a loss’. (Mother of a stillborn baby)

The reaction amongst those who knew about a stillbirth in the community was similar to the families: understanding and supportive if it was the first and only time a woman was having a stillbirth, but if a woman experienced more than one stillbirth, it was considered a bad omen. Such a woman's contributions in village meetings would not be taken seriously, and other women might be more likely to question her worth.

### Coping mechanisms and support structures

Regardless of whether a woman experienced one or multiple stillbirths, support from her husband and extended family was thought to be critical to recovery. Participants reported that if a woman was supported by her husband, she became stronger quickly and even conceived again quickly. Older women helped with household chores so the mothers could recover. Mothers revealed that believing in God and comfort and counselling from sympathetic health workers were sources of strength. However, participants reported that mothers of stillbirths were encouraged by families and health workers to conceive immediately to help them cope with the loss, which was not always welcome advice. Some reported the fact that they themselves survived death helped them to cope with the grief and pain, especially in the case of those with other children at home.

### Practices that affect stillbirth visibility as a community and public health concern

Study participants reported that compared to other deaths in the community, even the death of a child that lived for a short time, stillbirths are not given a proper burial, and that mock graves are used to taunt families rather than honour the baby's memory. It was frequently stated that the baby who did not cry cannot be buried in the same graveyard with adults or babies that cried, because it cannot communicate while in the spirit world. Burial practices for stillbirths ranged from clan to clan and rituals carried out when a stillbirth was brought home differed. However, it was reported that most religions did not acknowledge unbaptised or uninitiated babies (including stillbirths and newborn deaths), which limited formal application of funeral rituals. A baby would be named or given a name at a special ceremony if born alive, but a dead baby would not undergo this ritual. Other differences highlighted include digging the grave using a stick instead of a hoe if the baby did not cry. In some clans, the stillbirth was reported to be buried in a hole dug on the veranda where water flows down from iron sheets, to level the grave with the ground to make it unnoticeable. Burial of a stillborn baby was reported to take place quickly and was more often conducted by women only, although in some cases men and other community members took responsibility:

When a pregnant woman loses a baby, she is left at home, and people nearby quickly gather and bury the dead baby. It is buried in the family compound. A grave is dug using a stick. No one wails as they do for an older child, but they feel sorry for the mother as she stays home without saying goodbye to her dead baby. (TBA)

It was reported that stillbirths are not announced in the village, and in most cases people are not widely informed. However, affected women do mark the event. After a stillbirth, it was reported that a mother may not bathe for many days and will use traditional herbs to cleanse her household. In some clans the mother of a stillborn baby was prohibited from going back to her parent's home for a long period, and she was not supposed to use a type of wood, *nkandwa*, for cooking. If a mother was to go to her parents she was supposed to throw a certain herb in the fire before setting foot in the house.

## Discussion

These interviews reveal that definitions, causes of death, and solutions have biomedical and spiritual dimensions. Although community members are aware that pregnancy loss occurs, a stillbirth is primarily considered an issue affecting the woman who has directly experienced the loss. Women and families in this context have limited options for support within existing structures.

One could not miss the repetitive use of the words *empuna* and *ekintu*, both referring to ‘a thing’ in reference to stillbirths. Similar to the use of the English word stillborn as a synonym for useless or ineffectual (7), it is unsurprising that the local term makes no reference to the life that has been lost. Defining the stillbirth as a thing that does not cry provoked different burial practices. In Tanzania, the differences in burial practices for ‘immature’ babies (most stillbirths) are due to the fact that they are not considered fully human and therefore potentially threatening (13).

We found that participants first described stillbirth to be medically related, followed by societal and spiritual causes. Syphilis in particular was described as a major factor, even though it is less likely to be a cause of stillbirth than malaria in this setting (6). Malaria was not commonly mentioned as an associated cause. Notably, domestic violence was raised as an important contributor to stillbirths and was reportedly widespread. One study in rural western Uganda found that up to 70% of men and 90% of women believed that violence against a female partner was justifiable in certain circumstances (14).

The issues that lead to delays in care-seeking and receiving care are well known in the literature (15, 16), but we were surprised at the level of knowledge in this community. A possible explanation could be learning from the many sessions women attend during pregnancy and the search for an explanation for their own loss. While unknown causes were associated with superstition, there was no indication that participants thought these stillbirths were inevitable, a finding seen in other African settings (17). The fear of being accused of having an induced abortion has been reported by women in other settings (13), but respondents in this study were concerned that even an intention to terminate the pregnancy could cause a stillbirth.

It is culturally assumed that a community will not grieve the loss of ‘a thing’. The burial of a stillborn baby is done in private, mainly by women, with little opportunity for public mourning or condolences. Women who experienced antepartum stillbirths in particular reflected the need to be ‘weeping in silence’ to avoid further stigmatisation for themselves and their families. Respondents did not mention peer support or seeking out other women who had experienced stillbirth as a mechanism to cope with the loss, although older women were mentioned as a source of physical help with chores and household responsibilities.

These findings reaffirm that despite the magnitude of stillbirths and the intersection with maternal and child health, the health system and community support structures have been largely unresponsive both in terms of prevention efforts and providing support to affected families (7, 13). To avoid blame and potential divorce, the couple was often counselled, even by health workers, to have another baby to ‘replace’ the baby who was stillborn, despite risks of conceiving quickly after loss (18). In addition to efforts to prevent stillbirths within the context of maternal and newborn care, there is a need for education to lessen the stigma and create supportive spaces for families to grieve. Experiencing a stillbirth is not an acute event; literature from high-income countries demonstrates that the trauma of stillbirths may remain up to 18 years after the loss (19). Such efforts include incorporating bereavement support in health worker training as well as engaging community structures, and sensitising communities to the problem beyond the individual woman or family (7).

A limitation of our study is the small number of women interviewed who had experienced a stillbirth, only one of whom had delivered outside a health facility. Mothers who delivered stillborn babies at home without accessing the healthcare system, or those experiencing additional barriers to disclosing stillbirths, were difficult to locate and their views may be under-represented. However, we triangulated data across interviews with mothers who experienced stillbirth, and interviews with other key actors who could speak to the experience of home deliveries and social norms, such as TBAs.

## Conclusion

In Uganda, as elsewhere, stillbirths represent a massive loss in terms of mortality but also an additional emotional burden to be borne by women. Stillbirth is also felt by the father and immediate family members, but in most cases it is not considered a community concern or a priority issue for health programming. The capacity of the health system needs to be built not only to account for and prevent stillbirths but also to follow up and provide physical, psychological, and emotional support to women and families who have experienced loss.

## References

[1] Lawn JE, Lee AC, Kinney M, Sibley L, Carlo WA, Paul VK (2009). Two million intrapartum-related stillbirths and neonatal deaths: where, why, and what can be done?. Int J Gynaecol Obstet.

[2] UNICEF (2008). UNICEF, state of the world's children 2008.

[3] Lawn JE, Blencowe H, Oza S, You D, Lee AC, Waiswa P (2014). Every newborn: progress, priorities, and potential beyond survival. Lancet.

[4] UNICEF, WHO, The World Bank, United Nations (2013). Levels and trends in child mortality: report 2013.

[5] WHO, UNICEF, UNFPA, The World Bank, United Nations Population Division (2014). Trends in maternal mortality: 1990 to 2013.

[6] Lawn JE, Blencowe H, Pattinson R, Cousens S, Kumar R, Ibiebele I (2011). Stillbirths: where? when? why? how to make the data count?. Lancet.

[7] Froen JF, Cacciatore J, McClure EM, Kuti O, Jokhio AH, Islam M (2011). Stillbirths: why they matter. Lancet.

[8] Waiswa P, Namazzi G, Kerber K, Peterson S (2014). Designing for action: adapting and implementing a community-based newborn care package to affect national change in Uganda. Glob Health Action.

[9] Waiswa P, Pariyo G, Kallander K, Akuze J, Namazzi G, Ekirapa-Kiracho E (2014). Effect of the Uganda Newborn Study on care-seeking and care practices: a cluster-randomised controlled trial. Glob Health Action.

[10] Waiswa P, Peterson SS, Namazzi G, Ekirapa EK, Naikoba S, Byaruhanga R (2012). The Uganda Newborn Study (UNEST): an effectiveness study on improving newborn health and survival in rural Uganda through a community-based intervention linked to health facilities – study protocol for a cluster randomized controlled trial. Trials.

[11] Pope C, Mays N (1995). Reaching the parts other methods cannot reach: an introduction to qualitative methods in health and health services research. BMJ.

[12] Graneheim UH, Lundman B (2004). Qualitative content analysis in nursing research: concepts, procedures and measures to achieve trustworthiness. Nurse Educ Today.

[13] Haws RA, Mashasi I, Mrisho M, Schellenberg JA, Darmstadt GL, Winch PJ (2010). These are not good things for other people to know’: how rural Tanzanian women's experiences of pregnancy loss and early neonatal death may impact survey data quality. Soc Sci Med.

[14] Koenig MA, Lutalo T, Zhao F, Nalugoda F, Wabwire-Mangen F, Kiwanuka N (2003). Domestic violence in rural Uganda: evidence from a community-based study. Bull World Health Organ.

[15] Thaddeus S, Maine D (1994). Too far to walk: maternal mortality in context. Soc Sci Med.

[16] Waiswa P, Kallander K, Peterson S, Tomson G, Pariyo GW (2010). Using the three delays model to understand why newborn babies die in eastern Uganda. Trop Med Int Health.

[17] Cecil R (1996). The anthropology of pregnancy loss: comparative studies in miscarriage, stillbirth and neonatal death (cross-cultural perspectives on women).

[18] Conde-Agudelo A, Rosas-Bermudez A, Kafury-Goeta AC (2006). Birth spacing and risk of adverse perinatal outcomes: a meta-analysis. JAMA.

[19] Christiansen D, Elklit A, Olff M (2013). Parents bereaved by infant death: PTSD symptoms up to 18 years after the loss. Gen Hosp Psychiatry.

# Sub article: ‘As soon as the umbilical cord gets off, the child ceases to be called a newborn’: sociocultural beliefs and newborn referral in rural Uganda

**DOI:** 10.3402/gha.v8.24386

**Published:** 2015-03-31

**Authors:** Christine K. Nalwadda, Peter Waiswa, David Guwatudde, Kate Kerber, Stefan Peterson, Juliet Kiguli

**Affiliations:** 1 School of Public Health, College of Health Sciences, Makerere University, Kampala, Uganda; 2 Health System Policy, Department of Public Health Sciences, Karolinska Institutet, Stockholm, Sweden; 3 Uganda Newborn Study, Iganga-Mayuge Health Demographic Surveillance Site, Kampala, Uganda; 4 Saving Newborn Lives, Save the Children, Cape Town, South Africa; 5 International Maternal and Child Health Unit, Department of Women and Children Health, Uppsala University, Uppsala, Sweden

**Keywords:** care-seeking, newborn, neonatal, qualitative, referral, sociocultural influences, Uganda

## Abstract

**Background:**

The first week of life is the time of greatest risk of death and disability, and is also associated with many traditional beliefs and practices. Identifying sick newborns in the community and referring them to health facilities is a key strategy to reduce deaths. Although a growing area of interest, there remains a lack of data on the role of sociocultural norms and practices on newborn healthcare-seeking in sub-Saharan Africa and the extent to which these norms can be modified.

**Objective:**

This study aimed to understand the community's perspective of potential sociocultural barriers and facilitators to compliance with newborn referral.

**Method:**

In this qualitative study, focus group discussions (n=12) were conducted with mothers and fathers of babies aged less than 3 months. In addition, in-depth interviews (n=11) were also held with traditional birth attendants and mothers who had been referred by community health workers to seek health-facility-based care. Participants were purposively selected from peri-urban and rural communities in two districts in eastern Uganda. Data were analysed using latent content analysis.

**Results:**

The community definition of a newborn varied, but this was most commonly defined by the period between birth and the umbilical cord stump falling off. During this period, newborns are perceived to be vulnerable to the environment and many mothers and their babies are kept in seclusion, although this practice may be changing. Sociocultural factors that influence compliance with newborn referrals to seek care emerged along three sub-themes: community understanding of the newborn period and cultural expectations; the role of community health actors; and caretaker knowledge, experience, and decision-making autonomy.

**Conclusion:**

In this setting, there is discrepancy between biomedical and community definitions of the newborn period. There were a number of sociocultural factors that could potentially affect compliance to newborn referral. The widely practised cultural seclusion period, knowledge about newborn sickness, individual experiences in households, perceived health system gaps, and decision-making processes were facilitators of or barriers to compliance with newborn referral. Designers of newborn interventions need to address locally existing cultural beliefs at the same time as they strengthen facility care.

The neonatal period is only 28 days and yet accounts for 44% of all deaths in children younger than the age of 5 years globally (1). In 2012, approximately 35,000 newborns in Uganda died during their first month of life (1). Three-quarters of these deaths occurred during the first 7 days of life (2), making the first week of life the most risky across the lifespan. The main causes of death during the newborn period are different from those in later childhood (3). More than any other age group, sick newborns can rapidly deteriorate and may die in a matter of hours. Yet, newborn health issues have only recently begun to receive specific attention in policy and programmes (4). Understanding local practices and beliefs during the first month and the first week are critical to optimise delivery of care and promote healthy behaviours to ensure newborn survival.[Fn FN0001_24386]

A period of confinement or seclusion following delivery has been described in different settings, including Tanzania, Senegal, and Bangladesh, and is believed to protect the newborn from ancestral spirits and bad omens as well as infections and the cold (5–7). This seclusion period may pose a barrier to seeking care. Recognising illness and making a decision to seek care was identified as the main avoidable factor in 50% of newborn deaths in one study in eastern Uganda (8). The World Health Organization currently recommends home visits during the first week of life (9) to improve newborn survival (9). Depending on national policy, home visit activities range from preventive and promotive care to curative care. In addition, counselling, assessing for danger signs and referring sick newborns for health-facility-based care, is conducted.

The Uganda Newborn Study (UNEST) is a cluster randomised controlled trial designed to test a community-based care package for maternal and newborn health, including home visits by community health workers (CHWs) during pregnancy and the first week of life (4). While newborn-care practices have been evaluated in UNEST (10, 11), there is little data on the importance of local sociocultural norms and practices related to timely care seeking. This qualitative research sought to understand the community's perspective of potential cultural barriers and facilitators to compliance with newborn referral. This paper is the eighth in a series on UNEST.

## Methods

### Study setting and design

The study was conducted in Iganga and Mayuge districts in eastern Uganda, within the Iganga–Mayuge Health Demographic Surveillance Site (HDSS) (12). The HDSS consists of 65 villages that are predominantly rural. Thirteen of these villages are peri-urban and form Iganga Town Council. The HDSS population was 70,000 at the time of the study, with the main economic activity being subsistence farming. Other occupations include small-scale businesses such as grain milling, market vending, motorcycle transport, and civil service employment. The predominant ethnic group in the HDSS is the Basoga, whose indigenous language is Lusoga. The HDSS is served by one 100-bed hospital, six health facilities offering out- and in-patient services, and seven outpatient-only facilities (13). In Uganda, CHWs are integrated into national Village Health Teams which serve as volunteers at village level (14).

Within UNEST, locally recruited CHWs worked in their respective villages and visited households twice during pregnancy and thrice during the first week after birth, on days 1, 3, and 7, for counselling on health behaviours and danger signs, and newborn assessments (11). The CHWs were trained and expected to identify the following signs of severe illness: convulsion, hotness or coldness, failure to breastfeed, rapid breathing in a calm baby, grunting, lack of movement on stimulation, yellowing, in-drawing of chest, umbilical cord with pus, and skin papules with pus, with special attention to babies who were born preterm and/or of low birth weight. During the home visit, the CHWs advised the mothers to seek care from a health facility immediately if a danger sign was noticed. In addition, CHWs also referred babies born outside health facilities for immunisation and postnatal care. In all the referral cases, a CHW wrote a referral note and gave it to the mother to present at the health facility for faster assistance than if they were to seek care without a referral.

This qualitative study was conducted between January and September 2012. Homogeneous focus group discussions (FGDs) and in-depth interviews (IDIs) with mothers, fathers, and traditional birth attendants (TBAs) were used to explore the community's perspectives in terms of knowledge of norms, perceptions, and practices during the newborn period which might impact on danger sign recognition, care-seeking practices, and decision-making processes, as well as referral compliance. The TBAs were considered important informants with unique perspectives on newborns and healthcare-seeking, since many women in the study area seek care from traditional healthcare providers, even if they do end up delivering at the health facility.

The field research team comprised a public health specialist and a medical anthropologist with social scientists as research assistants.

### Sampling frame and sampling procedures

The selection of study participants was purposive, targeting community members believed to have rich information about sociocultural factors that could influence compliance with newborn referral advice. Eight rural and four peri-urban villages were randomly selected from the 65 villages in the study area to host the FGDs and IDIs. FGDs were categorised by geographical location, sex (male/female), age (18- to 25-year-old and 26- to 49-year-old females were split), and place of residence (rural/peri-urban). The mothers who participated in IDIs were further categorised according to compliance with referral (complied/did not comply). The rationale behind categorisation was to obtain maximum variation in the views of the study participants (15). Attributes such as geographical location and mother's characteristics have been documented as influencing utilisation of healthcare services (16).

### Recruitment of study participants

Male and female participants for both the FGDs and IDIs were purposively selected from the villages with the assistance of the local village head. Participants were required to have children aged less than 3 months in order to identify individuals with knowledge and experience of the current norms and practices surrounding newborn care in this setting. UNEST staff and local CHWs provided information used to identify the homes of mothers who participated in the IDIs. None of the persons invited for interviews or discussions declined to participate.

### Data collection and processing

Research assistants whose mother tongue is Lusoga and who were experienced in qualitative data collection methods participated in data collection either through note-taking or moderating the discussions. They were trained on the study objectives and familiarised with the discussion and interview guides. Two pilot FGDs and one IDI were conducted in a village neighbouring the study area to test the flow of the discussions and interviews and how long each would last. Thereafter, some questions were rephrased for clarity and others rearranged to facilitate logical flow of the discussion.

A total of 12 FGDs were conducted. Each FGD comprised 5–10 participants and lasted no longer than 2 h (17). In addition, IDIs were conducted with TBAs (n=3) and women who had a sick newborn and subsequently received a referral by a CHW to seek further care (n=8). This was done to triangulate the issues expressed during the FGDs and deepen understanding of the social and cultural factors influencing care-seeking.

The FGD guide was based on questions formulated according to themes from previous research findings, but remained flexible to address emerging issues during the discussions. IDI participants were asked similar introductory questions as in the FGDs, but were also asked and probed for information such as why they complied with referral or why they did not comply, and who was responsible for this decision making. In the case of TBAs, their role in caretakers’ compliance and decision making was also ascertained. The FGDs and IDIs were held at venues agreed upon by the participants and free from interruption such as from onlookers and traffic.

All interviews and discussions were conducted in Lusoga, the main local language in the study area. They were all tape-recorded and notes were taken. The note takers immediately expanded the notes after the interviews and discussions, in preparation for supplementing the recordings at a later date during the verbatim transcription in cases where recordings were not audible enough. Emerging issues were identified and explored further in subsequent interviews and discussions.

The research assistants provided verbatim transcriptions of the recordings with direct translation into English. Local terms that could not easily be translated into English were kept in Lusoga. The data were analysed using content analysis (18). The conventional content analysis approach was used, where codes are directly derived from the data text (19). The FGD and IDI scripts were independently read by three people: the principal investigator, a medical anthropologist, and a social scientist. These readings were used to generate codes that were discussed and agreed upon during a review meeting and used for data analysis. The principal investigator developed the final code book, closely guided by the medical anthropologist. The code book was accessible to all research team members.

The text data were systematically synthesised. First, they were coded into meaning units, which were grouped into categories, followed by sub-themes, and then the final themes as agreed upon by the study team. This analysis was led by the medical anthropologist. The ATLAS.ti version 7.0 software program was used for qualitative data management and analysis. Further development of meaningful units and interpretation was done by all of the authors. Disagreements were resolved through consensus discussions.

### Ethical considerations

The protocol for this study was reviewed and approved by Makerere University College of Health Sciences, School of Public Health Higher Degree Research Committee, and the Uganda National Council of Science and Technology. The research team members explained the purpose of the study to the participants and emphasised that enrolment into the study was voluntary. Individual written consent was sought from both FGD and IDI participants. Illiterate participants were verbally informed of the terms of the study and provided consent through thumbprints. Only participants aged 18 years and above were recruited for interviews and discussions. No money or incentives were offered to the study participants, although a soft drink was given to each participant during the discussions. Interviews and discussions took place in privacy, and to ensure confidentiality, FGD participants were assigned identification numbers during discussions and transcription. In addition, all files were password protected.

## Results

The sociodemographic profile of the participants is presented in Table 1. A total of 48 women participated in the eight FGDs and eight IDIs, with a mean age of 28.9 years and range of 18–44 years. The majority of participants were married (n=44), had attained primary education (n=34), and were engaged in subsistence farming (n=42). Three TBAs were interviewed, aged 60, 50, and 48 years. A total of 23 men participated in four men's FGDs, with a mean age of 39.7 years and range of 22–57 years. The mean number of children fathered by these participants was 5 with a range of 1–14. The men were mainly subsistence farmers (n=9); an equal number (n=9) had attained ordinary-level secondary education. Amongst the eight women interviewed for IDIs, four mothers had sought referral care for their newborns (complied), and four women had not.

**Table 1:** Sociodemographic characteristics of men and women who participated in FGDs and IDIs

Variables	Men N=23 n (%)	Women N=48 n (%)
District		
Iganga	12 (52)	16 (33)
Mayuge	11 (48)	32 (67)
Residence		
Peri-urban	10 (43)	14 (29)
Rural	13 (57)	34 (71)
Marital status		
Single/never married	5 (22)	4 (8)
Married	18 (78)	44 (92)
Education status^a^		
Primary	9 (39)	34 (71)
Secondary ordinary	6 (26)	8 (17)
Secondary advanced	2 (9)	1 (2)
Tertiary	3 (13)	0 (0)
No education	3 (13)	5 (10)
Occupation^b^		
Peasant	14 (61)	40 (83)
Businessman	6 (26)	4 (8)
Catering	0 (0)	1 (2)
Housewife	0 (0)	2 (4)
Tailor	0 (0)	1 (2)
Head teacher	1 (4)	0 (0)
Primary teacher college tutor	1 (4)	0 (0)
Student	1 (4)	0 (0)

a Years of formal education: primary (1–7 years), ordinary-level secondary (8–11 years), advanced level secondary (12–13 years), and tertiary (14+ years).

b Categories for men and women do not add up to 100% due to rounding off of the values.

The results of the FGDs and IDIs revealed three broad themes that may influence care-seeking and compliance with referral: community understanding of the newborn period and cultural expectations; the complexity of community health actors; and caregiver knowledge, experience and decision-making autonomy (Table 2).

**Table 2:** Qualitative data analysis of the FGD and IDI feedback transcribed verbatim

Text/verbatim extract	Meaning unit	Condensed unit	Sub-theme	Themes
We call the baby ‘nakaghwele’. ‘Nakaghwele’ is the newborn and ‘omwibo’ is the mother. Because she is still in the postnatal period ‘akali mwibo’	‘Nakaghwele’ is the newborn and ‘omwibo’ is the newly delivered mother	Cultural terms for newborn and mother	Newborn and mother cultural terms	Understanding newborn definition
‘Nakaghwele’ starts at 1 day up to: 6 weeks …, 3 months …, 6 months when he/she starts eating and drinking …, starts to crawl/walk …, ends at 2 years	Newborn period defined in various ways	Varying definitions of newborn in community	No common newborn definition	Understanding newborn definition
Now, we the Basogas have cultural norms …, A baby with an umbilical cord does not come out of the house and doesn't cross the road unless when sick	A baby with an umbilical cord does not come out of the house	Newborn kept in house till umbilical cord detaches	Seclusion period	Understanding newborn period and cultural expectation
It's to all, irrespective of boy or girl; the cord must go off before he/she is brought out of the house	Both female and male newborns are kept inside until cord detaches	Male and female babies treated the same	Seclusion norm – no differentiation by sex	Understanding newborn period and cultural expectation
If Sande (CHW) gives me a referral form, I don't line up; when I reach there, I just show them the referral form and they will work on me; you don't have to wait like the one without a referral form	Mothers referred by CHWs with a referral note are attended to quickly at health facilities	CHWs’ referrals recognised by health workers	CHWs link mothers to health facilities	Role of community health actors
No, we go there because clinics are near and easy to access. And still it depends on the baby's situation; you may just go and get first aid from clinics before you proceed to the hospital	Seek care from private clinics due to easy access and for first aid	Seek care from private clinics	Private clinics offer services	Role of community health actors
There are times when you give birth to a child and he/she develops what they call ‘bidama’ in Lusoga, you have to take the child for traditional intervention	Local/traditional diseases are treated at traditional healers	Traditional illness requires traditional treatment	Care from traditional sector	Role of community health actors
At Musawo Monica's clinic in the trading centre and that is where she was immunised from because they send vaccines from Magada every end of the month	Mothers are aware and immunise children at outreaches	Private clinics used for immunisation outreaches	Public–private partnership	Role of community health actors
When the body changes and becomes yellow, child has a problem …, if the baby has been breastfeeding well, the feeding pattern reduces or it cannot suckle	Baby has a problem when he/she turns yellow …, if he/she reduces or stops breastfeeding	Yellowing, failure to breastfeed show baby is unwell	Knowledge of danger signs	Caregiver knowledge
Sometimes if the past experience from Nakavule (Hospital) was very bad so you are forced to go to clinics	Go to clinics due to bad experience at hospital	Bad experience at hospital	Past experience at health facility	Caregiver experience
When you are a husband at home, yet you don't have money, the one who has money decides	The one who has money is the one who decides	Decision depends on availability of money	Both mother and father can decide	Decision-making autonomy

### Community understanding of the newborn period and cultural expectations

All the FGD and IDI participants mentioned and agreed that the local term for a newborn in this setting is *nakaghele* while the mother is known as *omwibo*, meaning ‘one who has just delivered’. There was no single common definition of the newborn period. Few respondents conveyed the epidemiological definition, that is, from birth through 28 days of life. Rather, often participants described different time-points as marking the end of the newborn period. These included commencement of supplementary feeding, attainment of specific growth milestones such as walking and ability to care for self. For example, one woman in the FGD said ‘A newborn can go up to 6 months because that is when the child starts eating and drinking’, while a man in another FGD said that ‘a newborn stops at 8 months because when my child starts walking it has come out of newborn size’.

The trained CHWs reportedly provided a consistent definition of the newborn period as that from birth to 1 month of age, as narrated by an informant during an interview: ‘The village health worker (*mentions the name of the CHW*) said a newborn is a 1-month-old baby’. However, the community's most common indicator of the end of the newborn period was umbilical cord detachment. The participants frequently reported that the umbilical cord detachment took place within a week after delivery, and this marked the end of the newborn period, as explained by one male FGD participant:

A child ‘nakaghwere’ [*the young one*] is the one who is kept in the house and the cord has not yet got off …, when it gets out of the house it stops being a newborn after 7 days, then it ceases to be a newborn. It is our cultural practice, as long as the cord gets off it ceases to be called a newborn.

In many discussions and interviews, a newborn was consistently described as ‘a child who is delicate’ and needed extra care and protection, including shielding from sunshine, cold, and disease vectors such as mosquitoes and rats. One man in the FGD explained that ‘you cannot just hold the newborn anyhow, you must be very careful with extra care …, not putting the baby in the sun and cold …, keep baby away from insects, even rats …, laughter …’. In another FGD, a woman explained that ‘A newborn is defined from day 1 of birth up to 1 year because you have to breastfeed, care about him all the time … and if left to the cold, it might get this fever pneumonia that comes with the cold’.

One of the distinctive norms is the postnatal seclusion or confinement period known as *ekisanda* (dry banana leaves). The practice does not have a specific number of days but it begins immediately after birth and continues until the newborn's umbilical cord falls off. This was explained by one male FGD participant: ‘The mother and *nakaghwele* (newborn) when still in *ekisanda* (seclusion) cannot move out until after the umbilical cord drops off … and at that moment it will be called *omwana wawa* (a grown-up child)’.

Culturally, mothers are expected to strictly observe this seclusion period and stay indoors, irrespective of their place of delivery. However, this was reported to be changing, especially in the peri-urban areas, as explained by one informant: ‘For us here in town we can't stay in house without moving out, you have to go to the market and buy food every day, even take the baby if sick …’ (IDI mother who complied with referral advice).

A woman in one peri-urban FGD said that it was possible to violate the seclusion period: ‘But nowadays for me …. I have to take my baby to the hospital when sick, whether the umbilical cord still on or not’ (mother, FGD). Similarly, a man in the FGD held in the peri-urban area also reported that health workers instruct them to take babies for immunisation within the first week after delivery: ‘The health workers teach us to take the child to the health centre for polio prevention, even though it has not yet made 7 days’ (father, FGD).

Traditionally, during this period, a woman who has just delivered is supposed to sleep on the floor on dry banana leaves or on a mattress with a few dry bananas leaves underneath. During this period, the baby is not supposed to be touched by outsiders, not even siblings. This seclusion period is intended to protect the baby against evil spirits and infections, and the cold that may lead to sicknesses such as pneumonia. There does not appear to be a difference in gender regarding the period of seclusion, as mentioned by one informant: ‘Whether a boy or a girl, they are not taken out so long as the umbilical cord is not yet off’ (IDI mother who did not comply with referral advice).

However, several participants indicated that in the case of a critical situation (e.g. a funeral of a close family member), a woman could be allowed to attend the funeral and burial. Often participants made provisions to violate the confinement and seek healthcare for sick babies and those who needed routine immunisation. This was one practice reported that was in conflict with seclusion practices. However, a few participants reported the desire to strictly observe the seclusion period unless a misfortune happened, as one woman said: ‘I cannot go out before the umbilical cord drops off – maybe if my mother or father died’ (mother, FGD).

After the umbilical cord falls off, a number of rituals, *okukuza omwana* (recognising that the baby is a grown-up), are performed, such as the naming ceremony. Other practices include ritual bathing, throwing away the dry banana leaves from the seclusion room, parents of the newborn engaging in sexual intercourse for the first time since delivery (or a ritual to symbolise the same), and feasting, as participants described:

Ceremonies are performed after the baby's cord has dropped off. We call it *okukuza omwana*. The mother throws away the dry banana leaves, the father and mother of the baby sleep together for the first time after delivery, and depending on the clan of the family, different foodstuffs like sim-sim sauce and goat's meat are prepared for the family members to eat. (IDI, TBA)

When the baby's umbilical cord is off, [the mother and baby] move outside, pour water on the roof and it comes and falls on the child. (father, FGD)

The umbilical cord is kept secure by the mother or given to the father of the newborn. The umbilical cord is kept to be used in another ceremony of confirming paternity at a later date.

### The role of community health actors

Community care providers such as CHWs, private drug sellers, and TBAs play various roles with regard to maternal and newborn care. Two TBAs reported that they refer sick newborns and pregnant mothers who are first-time mothers and those who have had five or more deliveries, as one explained: ‘I once sent a mother to take the baby who had a cord with pus to Iganga Hospital; the mother had put powder on it’.

Many of the FGD participants reported that CHWs encourage community members to seek care from health facilities, and also agreed that families comply with referral advice given by the CHWs, as mentioned by one of the women in the FGD: ‘Our village health worker told us that before we buy drugs for the baby from shops, we should first take them to Government health centres. So we also follow her advice’.

The participants continued to mention that CHWs offered them health education about newborn care and motivated them to seek referral care by giving them referral notes, as one woman in the FGD stated: ‘When the CHWs are teaching us, they tell us about the bad signs, when you realise that the baby has some of those bad signs, you go to the CHW …, the CHW gives you a note (*referral slip*) and you go to the health facility’.

However, for some community members, the first point of care-seeking following referral may not be the health facility, even when they have been counselled. One informant reported that some caretakers first seek care from drug shops and traditional providers, given their proximity, especially during the seclusion period. Depending on the baby's outcome after receiving initial care, they may decide to proceed to a health facility, as stated by a mother during an interview: ‘Some people first go to drug shops and traditional healers and then to the Government health facilities if the baby does not get well’.

Another reason given for not immediately seeking formal care following the CHWs’ referral advice was the belief that some conditions are best managed by traditional healers or community specialists. Compliance with referral can be based on whether the cause of the sickness is believed to have a traditional or biomedical solution. One mother stated during the interview: ‘… when the baby has a strange disease like the yellow body, you go to a traditional healer’ (IDI mother who did not comply with referral).

### Caretaker knowledge, experience, and decision-making autonomy

#### Caretaker knowledge of newborn danger signs

FGD and IDI participants often mentioned symptoms that indicate severe illness, such as failure to breastfeed, convulsions, and umbilical cord with pus. These symptoms are in agreement with those classified as newborn danger signs by biomedical health workers. However, signs which do not qualify as danger signs were occasionally mentioned by participants, and these included ‘false teeth’ (20), ‘extra digits’, and ‘tongue tie’. The participants agreed among themselves that newborns with danger signs should be taken for facility-based care. The community's knowledge of danger signs seemed to influence compliance with referral. Caregivers who could describe the danger signs promoted by CHWs were positively inclined towards complying and seeking referral care, as stated by one woman in the FGD: ‘A baby may have a problem with the umbilical cord, like you see blood or pus coming out of the cord. You go to the CHW if the CHW is able to help; she can help and thereafter can send you to a health facility’.

#### Caretaker experiences

During the FGDs and IDIs, participants mentioned and narrated negative experiences from previous interactions with the health system. These reflected poor quality of care at the health facilities, such as rude health workers, stock-outs of medicines, and absenteeism from work, especially on weekend days. These were highlighted as possible barriers to newborn referral compliance. Participants expressed the view that some health workers were rude, and thus discouraged caretakers from seeking referral care. It was also reported that health workers at lower-level facilities did not work during evening hours or weekends, so that if a newborn is referred during a weekend, the caretaker has few options. Similarly, perceived poor quality of care, including lack of drugs and supplies, deters caretakers from seeking a higher level of care, especially if there is much distance to the facility.

Some participants reported that there were drug stock-outs at health facilities and that they were told to buy medicine for their newborns. With such experiences, some caretakers may opt for other alternatives to treat the sick newborns. Acknowledging these constraints and perhaps reflecting pervasive beliefs in traditional remedies, health workers were also reported to have advised parents to seek care in the informal health sector, as one mother reported: ‘Sometimes health workers when they don't have that medicine they tell you to go back and (get) local medicine’ (mother, FGD).

Experiences with stock-outs in particular were raised as potential barriers to future compliance with referral. At the same time, positive experiences such as the presence of qualified personnel and high technology such as blood transfusion services were possible motivators to seeking care from health facilities, as expressed by one informant mother who complied with referral: ‘I took the baby to Nakauvle (district hospital) because there are doctors who can put in blood’.

Informants who did not comply with referral reported experiences related to the health status of the mother and family members, poverty, and weather conditions as factors that prevented them from complying with referral. One of the informants explained: ‘I was unwell, still bleeding and could not walk to take the baby’. Another mother said ‘I did not go because it had rained the whole day and I had no money for transport’, and another that ‘I was caring for another sick child. I decided to wait and go another day’.

#### Decision making

According to most FGD participants, the decision to seek referral care when recommended rested primarily with the father or mother rather than other family or community members. They were in agreement with a TBA who reported that as an outsider to the family, she was not responsible for making any decisions when the baby was referred by the CHWs; her role ends at delivering the babies. She explained: ‘When the CHW (*mentions CHW's name*) tells the mother to take the baby to Nakavule (Hospital), it is for the family to decide if they take the baby, not me – my job is to help the mother deliver the baby’.

Occasionally, some women expressed that while the mother can take a decision to seek care, she often does not have the finances to be able to do so. Other participants were of the view that since the father provides money, he ultimately makes the decision, as narrated by one woman in the FGD: ‘You a woman may decide to take the baby after being referred, but if you don't have the money you find yourself unable to go’. Another woman in the FGD added: ‘Now if the baby refuses to breastfeed or has a high temperature, I told you, our village health workers write letters for us and tell us to take the babies to the health facility, but it's for the husband to decide’. During the interview, another mother emphasised that: ‘He has to decide because he is the one to pay the hospital bills, so you cannot force him’ (IDI mother who did not comply with referral).

## Discussion

Sociocultural factors influencing community newborn referral compliance in eastern Uganda were broadly categorised into those related to the community understanding of the newborn period and expected practices around this time, the varying and overlapping roles of community health actors, and caretaker knowledge, experience, and decision making. These were the main themes that emerged linked to whether or not families comply with community referrals. In general, caretakers portrayed willingness to comply with newborn referrals, for example, from CHWs, but there were also barriers to their ability to seek care.

We found a discrepancy between the biomedical and community definitions of a newborn. While some of the study participants described the newborn period as lasting through the first year, the predominant definition limited the newborn period until the baby's umbilical cord stump fell off. The newborn period was linked to a number of strongly held traditional practices, including seclusion for mother and baby in some families, which may affect care and care-seeking. A seclusion period is well described in the literature from other settings (7, 21–23), although in this setting the time was shorter than the commonly held 40 days of life (5, 6). While this practice provides an opportunity for the mother to rest and regain strength, establish breastfeeding, and bond with her baby, it might also limit the ability to seek care outside of the home during the time of greatest risk (5, 6, 24). However, participants in this setting reported a possibility of breaking the seclusion and seeking referral care if the illness is severe enough. Participants expressed that the seclusion period is a traditional practice, but that if necessary the baby can be taken for facility-based care.

Recognition of danger signs and perceiving certain symptoms as important, eliciting seeking of care, may have been due to counselling by the CHWs. For example, a bleeding cord or one with pus was associated with the baby having severe illness, and prompted caretakers to comply with referrals. CHWs are recognised in their capacity as health promoters for women and newborns in this setting, a finding that has been also been echoed in western Uganda, where the integrated community case management programme for children under 5 years of age has been rolled out (25).

Both mothers and health workers noted that the referral slips given to women by CHWs to present at health facilities increase legitimacy and facilitate prompt treatment by the health workers (25). However, families do not always go directly to formal health services after referral. Traditional providers and local private clinics and drug sellers were more likely to be the first point of care, especially in rural areas and if the baby is referred in the evening or on weekends. This has implications for newborn survival, despite the increased care-seeking, since such places have limited capacity for and quality of sick newborn management, and may lead to delays in receiving appropriate care. Referrals from community level were not just given by CHWs, and the reported referral by TBAs is important emerging information that needs more study.

In one review of home-based newborn care in Bangladesh, the use of traditional care and home remedies was preferred to formal health services (26). Only when the baby did not improve at home did the caretaker consider seeking care from the health facilities. In Ghana, only 61% of newborns with a serious illness were taken outside the home to seek care (27). This phenomenon of seeking facility-based care after failure to improve has also been reported amongst older children in Tanzania (28). Informal healthcare providers play an important role in offering health care because of their proximity and availability in the communities. Involving them early on in community-based interventions should be explored, and it should also be examined how they can support care-seeking from the formal health sector for newborn illness and reduce delays in receiving newborn care.

Decisions to seek referral care ultimately depend on the availability of finances, which are usually controlled by the husband, who is most often the head of the household. In general we found that the source of the decision to seek care was mixed. In other families the father was viewed both as an overall decision-maker in the home in addition to providing the money. This is similar to a finding reported in a study that explored how decisions are made in seeking maternal care in Burkina Faso (29). The same opinion that men dominate in decision making at household level was reported by Mbizvo and Adamchak in Zimbabwe (30). However, we also found that mothers often believed that they had a right and that it was their role to make a decision in relation to the baby's health, although sometimes they may not have had the money to go for referral care.

Past experiences with seeking care from the formal sector and perceived quality of care seemed to have a role to play in compliance with the CHWs’ newborn referral advice. Past experience with drug stock-outs, health work absenteeism, and rudeness could lead to non-compliance in this setting, as reported by Waiswa et al. (31). This experience is not unfounded; previous studies show that families in Uganda may not receive high-quality care for their sick children once they do seek care (25, 32, 33). The health system quality gaps thus need to be overcome to further motivate caregivers to comply with referral advice.

This study has some limitations. Transferability of some of the findings may only apply to the study setting and areas with similar cultural norms. However, findings regarding decision making and health system gaps as barriers to compliance with newborn referral may be more widely applicable. The use of FGDs first followed by IDIs may not be considered the normal approach, but in this study we need to understand the broader picture before narrowing down to the participants who experienced the newborn referrals.

We aimed at achieving trustworthiness in this study by triangulation of the data collection methods, providing a description of quotes from the participants for accuracy of representation, describing the data analysis process to show that the findings emerged from the data, with the team comprising experienced researchers in the method and topic of study.

## Conclusion

In this setting a discrepancy exists between the biomedical and community definition of the newborn period. There were a number of sociocultural factors that could potentially affect compliance to newborn referral. Despite the widely practised norm of a seclusion period, acceptance to seek referral care during this period seems feasible, particularly if the illness in the newborn is perceived as severe. Social factors, including knowledge about newborn sickness, individual experiences at households, perceived health system gaps and decision making processes, were facilitators of or barriers to compliance with newborn referral.

Designers of interventions for newborns need to address locally existing cultural beliefs at the same time as they strengthen facility care. Additional emphasis on all danger signs should be made during CHW counselling sessions in pregnancy and the early postnatal period. The differences between illnesses with a perceived biomedical solution compared to those considered suitable for treatment by traditional remedies and the subsequent impact on care-seeking patterns requires more investigation. Similarly, more research is needed to identify factors that can reduce delays in care-seeking, including how to better engage trusted community health actors such as TBAs.

## Footnotes

§ Senior authors contributed equally.

## References

[1] UNICEF, WHO, The World Bank, UNFPA (2013). Levels and trends in child mortality, report 2013. http://www.who.int/maternal_child_adolescent/documents/levels_trends_child_mortality_2013.pdf?ua=1.

[2] Lawn JE, Cousens S, Zupan J (2005). 4 million neonatal deaths: when? where? why?. Lancet.

[3] Liu L, Johnson HL, Cousens S, Perin J, Scott S, Lawn JE (2012). Global, regional, and national causes of child mortality: an updated systematic analysis for 2010 with time trends since 2000. Lancet.

[4] Mbonye AK, Sentongo M, Mukasa GK, Byaruhanga R, Sentumbwe-Mugisa O, Waiswa P (2012). Newborn survival in Uganda: a decade of change and future implications. Health Policy Plan.

[5] Winch PJ, Alam MA, Akther A, Afroz D, Ali NA, Ellis AA (2005). Local understandings of vulnerability and protection during the neonatal period in Sylhet district, Bangladesh: a qualitative study. Lancet.

[6] Mrisho M, Schellenberg JA, Mushi AK, Obrist B, Mshinda H, Tanner M (2008). Understanding home-based neonatal care practice in rural southern Tanzania. Trans R Soc Trop Med Hyg.

[7] Niang C (2003). Formative research on peri/neonatal health in the Kébémere health district, Senegal.

[8] Waiswa P, Kallander K, Peterson S, Tomson G, Pariyo GW (2010). Using the three delays model to understand why newborn babies die in eastern Uganda. Trop Med Int Health.

[9] WHO, UNICEF, USAID, Save the Children (2009). WHO-UNICEF joint statement on home visits for the newborn child: a strategy to improve survival.

[10] Waiswa P, Peterson S, Tomson G, Pariyo G (2010). Poor newborn care practices – a population based survey in eastern Uganda. BMC Pregnancy Childbirth.

[11] Waiswa P, Pariyo G, Kallander K, Akuze J, Namazzi G, Ekirapa-Kiracho E (2014). Effect of the Uganda Newborn Study on care-seeking and care practices: a cluster-randomised controlled trial. Glob Health Action.

[12] Turyatemba J (2011). Iganga-Mayuge DSS at the Helm of Research.

[13] Namazzi G, Waiswa P, Nakakeeto M, Nakibuuka VK, Namutamba S, Najjemba M (2014). Strengthening health facilities for maternal and newborn care: experiences from rural eastern Uganda. Glob Health Action.

[14] Health Education and Promotion Division (2010). Village health team strategy and operational guidelines.

[15] Coyne IT (1997). Sampling in qualitative research. Purposeful and theoretical sampling; merging or clear boundaries?. J Adv Nurs.

[16] Molyneux CS, Mung'Ala-Odera V, Harpham T, Snow RW (1999). Maternal responses to childhood fevers: a comparison of rural and urban residents in coastal Kenya. Trop Med Int Health.

[17] Krueger AR (1998). Analysing and reporting focus group results.

[18] Graneheim UH, Lundman B (2004). Qualitative content analysis in nursing research: concepts, procedures and measures to achieve trustworthiness. Nurse Educ Today.

[19] Hsieh H-F, Shannon SE (2005). Three approaches to qualitative content analysis. Qual Health Res.

[20] Nuwaha F, Okware J, Hanningtone T, Charles M (2007). False teeth “Ebiino” and Millet disease “Oburo” in Bushenyi district of Uganda. Afr Health Sci.

[21] Almedom AM (1991). Infant feeding in urban low-income households in Ethiopia: I. the weaning process. Ecol Food Nutr.

[22] Thairu L, Pelto G (2008). Newborn care practices in Pemba Island (Tanzania) and their implications for newborn health and survival. Matern Child Nutr.

[23] Mrisho M, Obrist B, Schellenberg JA, Haws RA, Mushi AK, Mshinda H (2009). The use of antenatal and postnatal care: perspectives and experiences of women and health care providers in rural southern Tanzania. BMC Pregnancy Childbirth.

[24] Mesko N, Osrin D, Tamang S, Shrestha BP, Manandhar DS, Manandhar M (2003). Care for perinatal illness in rural Nepal: a descriptive study with cross-sectional and qualitative components. BMC Int Health Hum Rights.

[25] Nalwadda KC, Naamala SH, Ssekitooleko J, Kerber K, Kallander K, Waiswa P (2012). Introduction of newborn care within integrated community case management in Uganda. Am J Trop Med Hyg.

[26] Darmstadt GL, Syed U, Patel Z, Kabir N (2006). Review of domiciliary newborn-care practices in Bangladesh. J Health Popul Nutr.

[27] Bazzano AN, Kirkwood BR, Tawiah-Agyemang C, Owusu-Agyei S, Adongo PB (2008). Beyond symptom recognition: care-seeking for ill newborns in rural Ghana. Trop Med Int Health.

[28] Simba DO, Kakoko DC, Warsame M, Premji Z, Gomes MF, Tomson G (2010). Understanding caretakers’ dilemma in deciding whether or not to adhere with referral advice after pre-referral treatment with rectal artesunate. Malar J.

[29] Somé DT, Sombié I, Meda N (2013). How decision for seeking maternal care is made-a qualitative study in two rural medical districts of Burkina Faso. Reprod Health.

[30] Mbizvo MT, Adamchak DJ (1991). Family planning knowledge, attitudes, and practices of men in Zimbabwe. Stud Fam Plann.

[31] Waiswa P, Kemigisa M, Kiguli J, Naikoba S, Pariyo GW, Peterson S (2008). Acceptability of evidence-based neonatal care practices in rural Uganda–implications for programming. BMC Pregnancy Childbirth.

[32] Kallander K, Hildenwall H, Waiswa P, Galiwango E, Peterson S, Pariyo G (2008). Delayed care seeking for fatal pneumonia in children aged under five years in Uganda: a case-series study. Bull World Health Organ.

[33] Peterson S, Nsungwa-Sabiiti J, Were W, Nsabagasani X, Magumba G, Nambooze J (2004). Coping with paediatric referral–Ugandan parents’ experience. Lancet.

# Sub article: Improving newborn care practices through home visits: lessons from Malawi, Nepal, Bangladesh, and Uganda

**DOI:** 10.3402/gha.v8.23963

**Published:** 2015-03-31

**Authors:** Deborah Sitrin, Tanya Guenther, Peter Waiswa, Sarah Namutamba, Gertrude Namazzi, Srijana Sharma, KC Ashish, Sayed Rubayet, Subrata Bhadra, Reuben Ligowe, Emmanuel Chimbalanga, Elizabeth Sewell, Kate Kerber, Allisyn Moran

**Affiliations:** 1 Save the Children, Washington, DC, USA; 2 School of Public Health, Makerere University, Kampala, Uganda; 3 Save the Children, Kathmandu, Nepal; 4 UNICEF, Kathmandu, Nepal; 5 Save the Children, Dhaka, Bangladesh; 6 National Institute of Population Research and Training, Ministry of Health and Family Welfare, Dhaka, Bangladesh; 7 Save the Children, Lilongwe, Malawi; 8 Department of Neonatology, Children's National Medical Center, Washington, DC, USA; 9 Save the Children, Cape Town, South Africa

**Keywords:** newborn, neonatal, postnatal, home visit, community-based, community health worker

## Abstract

**Background:**

Nearly all newborn deaths occur in low- or middle-income countries. Many of these deaths could be prevented through promotion and provision of newborn care practices such as thermal care, early and exclusive breastfeeding, and hygienic cord care. Home visit programmes promoting these practices were piloted in Malawi, Nepal, Bangladesh, and Uganda.

**Objective:**

This study assessed changes in selected newborn care practices over time in pilot programme areas in four countries and evaluated whether women who received home visits during pregnancy were more likely to report use of three key practices.

**Design:**

Using data from cross-sectional surveys of women with live births at baseline and endline, the Pearson chi-squared test was used to assess changes over time. Generalised linear models were used to assess the relationship between the main independent variable – home visit from a community health worker (CHW) during pregnancy (0, 1–2, 3+) – and use of selected practices while controlling for antenatal care, place of delivery, and maternal age and education.

**Results:**

There were statistically significant improvements in practices, except applying nothing to the cord in Malawi and early initiation of breastfeeding in Bangladesh. In Malawi, Nepal, and Bangladesh, women who were visited by a CHW three or more times during pregnancy were more likely to report use of selected practices. Women who delivered in a facility were also more likely to report use of selected practices in Malawi, Nepal, and Uganda; association with place of birth was not examined in Bangladesh because only women who delivered outside a facility were asked about these practices.

**Conclusion:**

Home visits can play a role in improving practices in different settings. Multiple interactions are needed, so programmes need to investigate the most appropriate and efficient ways to reach families and promote newborn care practices. Meanwhile, programmes must take advantage of increasing facility delivery rates to ensure that all babies benefit from these practices.

Nearly all deaths in newborns (99%) occur in low- or middle-income countries (1). While there has been global progress towards United Nation's (UN) Millennium Development Goal 4 to reduce mortality in children younger than age 5, only 23 of 75 priority countries are on track to meet their goals (2). Reducing the number of deaths during the newborn period is increasingly important to reaching those targets, because neonatal mortality now accounts for 43% of under-5 mortality (3).

In 2011, the World Health Organization (WHO) led a review of evidence of the impact of various reproductive, maternal, newborn, and child health interventions and recommended essential interventions for scale-up. Several interventions for newborns were recommended for delivery at community level, including promotion and provision of thermal care, early and exclusive breastfeeding, and hygienic cord care (4). Table 1 outlines ways in which these practices benefit the newborn.

**Table 1:** Benefits of newborn care practices promoted at community level

Practices	Specific behaviours	Benefits to newborn
Breastfeeding	Initiation of breastfeeding within 1 hour after birth	Reduced risk of death, particularly from infectious disease due to protective factors in colostrum and breast milk and protection
	Exclusive breastfeeding for 6 months	from ingestion of contaminants found in water, other fluids, and
	Continued breastfeeding with safe complementary foods after 6 months	food with exclusive breastfeeding (5) Reduced risk of morbidity, including gastro-intestinal infections and respiratory infections (6)
Cord care	Cord cut with sterile blade and cord care with sterile tie/clamp	Reduced risk of death and morbidity due to tetanus and sepsis, omphalitis, cord infection (7–10)
	Nothing harmful applied to cord (topical antibiotic/antiseptic may be recommended in some settings)	
Thermal care	Immediate drying	Reduced risk of hypothermia and sequelae including (11–16)
	Delayed bathing	∘Death
	Wrapping in clean cloth and/or skin-to-skin care	∘Infection ∘Central nervous system depression leading to bradycardia, apnoea, and poor feeding ∘Coagulation defects, haemorrhage

One method for promoting these practices in the community is through home visits, and there have been a number of studies demonstrating that home visits by trained community health workers (CHWs) can change newborn care practices (17–20). Countries with high neonatal mortality have increasingly adopted postnatal home visit policies (2) in response to the 2009 release of the WHO and United Nations Children's Fund Joint Statement recommending home visits during pregnancy and soon after delivery to reduce newborn deaths (21). Save the Children's Saving Newborn Lives (SNL) programme supported ministries of health and partners in Malawi, Nepal, Bangladesh, and Uganda to develop and test newborn programmes that use existing health system structures to deliver services for newborns, including home visits by community-based employees or volunteers (collectively called CHWs) affiliated with the Ministry of Health or other government agencies (22–25). Pilot areas were selected for implementation and learning in all countries. In pilot areas of Malawi, Nepal, and Bangladesh existing CHWs were given in-service training and support to do home-based maternal and newborn care and counselling, and were encouraged to integrate these home visits into their work. In Uganda, Ministry of Health guidelines were used to select new volunteer CHWs (called Village Health Teams or VHTs) because the VHT policy was in place, but the programme had not yet been scaled up. Selected CHWs were trained in a maternal and newborn care package.

The purpose of this analysis was to evaluate population-level changes in selected newborn care practices over time in pilot areas in four countries with different health system contexts and to learn whether women who received home visits from CHWs were more likely to report use of these practices.

## Methods

### Settings and programme descriptions

Programmes in all four countries included a package of community-based care for women and newborns delivered through a series of home visits during pregnancy and after birth. To deliver these services, programmes used existing (or sanctioned) community-based health workers or volunteers and support systems, with SNL and other partners providing training in maternal and newborn care; supplies required to deliver maternal and newborn services; and support for supervision, monitoring, and community engagement. In addition to home visits, programmes also included facility strengthening, mainly through training, supplies, and supervision, although the extent of this varied across countries. Likewise, community engagement efforts differed: CHWs in Malawi organised ‘core groups’ for planning and decision making; CHWs in Nepal worked with existing mothers’ groups; community leaders in Bangladesh attended orientation sessions; and research staff in Uganda sensitised district staff, traditional birth attendants, health workers, and private providers about the interventions and the study.

Approximately 600 CHWs in Malawi, 850 in Nepal, 100 in Bangladesh, and 61 in Uganda were trained on maternal and newborn care in pilot intervention areas. Duration of trainings ranged from 5 to 9 days. CHWs were instructed to make 2–4 home visits during pregnancy and 2–4 home visits after the birth (the number of visits varied across countries). Existing supervision systems were strengthened to support and encourage visits. The type of compensation that CHWs received was different and included salaries, stipends, and small travel allowances. CHWs in Nepal received compensation tied to the number of visits (see Table 2). As a result of these and other factors, coverage of visits varied widely across countries (26).

**Table 2:** Community worker cadre, characteristics, training, and incentives

	Bangladesh		Malawi	Nepal	Uganda
Cadre name	• Family Welfare Assistant (FWA) • Health Assistant (HA) – Females only • Community Nutrition Promoter (CNP)		Health Surveillance Assistant (HSA)	Female Community Health Volunteer (FCHV)	Village Health Team (VHT)
Characteristics	FWA/HA:	CNP:			
• Gender	• Female	• Female	• Mostly male	• Female	• Mixed
• CHW: pop. ratio	• 1:6,000–7,000	• 1:1,250	• 1:1,000–2,000	• 1:400^a^	• 1:1,000
• Education level	• Secondary education	• Primary (not strict)	• Secondary	• Literate, primary preferred	• Literate
• Employment status	• Govt-salaried employee	• Volunteer (stipend)	• Govt-salaried employee	• Volunteer	• Volunteer (travel allowance)
• Recruitment	• From communities	• From communities	• Centrally recruited	• From communities	• From communities
• Pre-service training	• 21 days (FWA) 6 weeks (HA)	• 24-day training	• 12-week training	• 18-day training	• None
Training in maternal newborn community package	5 days		9 days (+6 days community mobilisation)	6 days	6 days
Paid incentives based on number of visits	No		No	Yes	No

a Catchment area population size varies in Nepal depending on terrain; 1:400 ratio is applicable to Terai region such as Bardiya.

Home visits included promotion of optimal maternal and newborn care and use of routine facility services; counselling on danger signs and care-seeking for mother and baby; screening for newborn danger signs by a physical assessment including checking the baby's temperature, breathing, and weight; and referral to a health facility when needed. Although coverage of home visits varied across countries, most mothers reported that key functions were likely to be performed when the visits occurred (26). The package in Nepal included curative care limited to CHW administration of one dose of oral antibiotics to newborns with possible severe bacterial infection. They then referred the sick newborn and caregiver to a health post to complete treatment with injectable antibiotics. In Nepal, CHWs were also expected to attend home deliveries and conduct resuscitation, if needed.

To help promote healthy newborn care practices, CHWs were equipped with counselling cards to use during home visits. Table 3 shows the visit schedule and the messages CHWs were trained to give women and families related to the newborn care practices evaluated in this study.

**Table 3:** Home visit schedule and messages given during home visits on newborn care practices

		Malawi	Nepal	Bangladesh	Uganda^a^

		Chitipa, Dowa, Thyolo districts	Bardiya district	Faridpur district	Iganga & Mayuge districts
# Pregnancy visits		3 (1st, 2nd and 3rd trimester)	4 (4th, 6th, 8th, 9th months)	2 (2nd and 3rd trimester)	2 (early as possible and 3rd trimester)
# Postnatal visits		2–3 (day 1 – home births only, 3, 8)	4 (day 1, 3, 7, 29)	3 (day 1, 2–3, 4–7)	2 (day 1–3, 5–7)
Early initiation of breastfeeding	Messages	Initiate breastfeeding within 30 min after delivery depending on feeding option chosen by mother	Initiate breastfeeding within 1 hour after birth	Initiate breastfeeding immediately and no later than 1 hour after birth	Put baby on the breast within 1 hour of birth
	When delivered	2nd and 3rd pregnancy visit 1st postnatal visit (home births)	3rd and 4th pregnancy visit 1st postnatal visit (home births)	All pregnancy visits	1st postnatal visit
Delayed bathing	Messages	Delay bathing the baby for 24 hours after birth	Newborn should not be bathed for at least 24 hours after birth	No bath within 3 days of birth	Delay bathing until after the 1st day
	When delivered	2nd and 3rd pregnancy visit 1st postnatal visit (home births)	3rd and 4th pregnancy visit 1st postnatal visit (home births)	All pregnancy visits	1st postnatal visit
Skin-to-skin care	Messages	Place the baby on the mother's abdomen for skin-to-skin contact to promote bonding	Place the newborn close to mother and make him/her warm	Place naked baby on mother's bare chest or abdomen within 24 hours of birth Mother and infant should be covered with a clean, dry cloth	Dry the baby. Then put the naked baby between your breasts. Cover him or her lightly with a dry cloth and keep in this position as much as possible during day and night.^b^
	When delivered	2nd and 3rd pregnancy visit 1st postnatal visit (home births)	3rd and 4th pregnancy visit 1st postnatal visit	All pregnancy visits	2nd postnatal visit
Cord care	Messages	Avoid applying traditional herbs or substances on the umbilical cord	Keep the cord dry and clean Do not put oil or anything onto the cord	Cord should be kept clean and dry after cutting Apply nothing to cord stump If cord is very soiled it may be cleansed gently with boiled water	Do not put anything on the cord, keep it clean and dry Let the cord stump dry and fall off on its own
	When delivered	All 3 postnatal visits	3rd and 4th pregnancy visit 1st postnatal visit (home births)	All pregnancy visits	1st postnatal visit

a Counselling cards indicate that counselling on these behaviours should occur during the first postnatal visits. According to the study PIs, the CHWs were encouraged to deliver messages during all pregnancy and postnatal visits during training and supervision.

b In the VHT manual, skin-to-skin care recommended for low birthweight babies. Study PI says VHTs in study area trained to recommend skin-to-skin care for all newborns.

The evaluation designs differed. In Malawi and Nepal, a before–after design was used with no comparison area. In Bangladesh, a before–after design was used with a comparison area. In Uganda, a cluster randomised controlled trial was done. In Malawi implementation areas were parts of three districts (Chitipa, Dowa, Thyolo) with a population of 711,000. In Nepal, implementation was in Bardiya district in the Terai area with a population of 460,000. In Bangladesh, implementation was in four rural unions in Madhukhaliupazila (sub-district) in Faridpur district with a population of 98,000, and an additional four unions in Nagarkandaupazila in Faridpur district were used as comparison areas. In Uganda, 65 villages in Iganga and Mayuge districts with a total population of 70,000 were randomly allocated – 33 to the intervention arm and 32 to the control arm. Additional details on programme design and evaluation have been published previously (26, 27).

### Data collection

Household surveys were conducted before implementation (baseline) and after at least 1 year of implementation (endline). Women with a live birth in the previous 12 months (past 3–12 months at endline in Bangladesh) were interviewed. Start of full implementation to endline data collection was 12 months in Malawi (June 2010–June 2011), 17 months in Nepal (January 2010–June 2011), 14 months in Bangladesh (April 2009–June 2010), and 25 months in Uganda (August 2009–September 2011). The data collection methods have also been described previously (26, 27). In Bangladesh and Uganda, data were also collected in comparison areas.

Caesarean deliveries were excluded from analysis for Malawi, Bangladesh, and Uganda since certain newborn care practices may be delayed after such births and the procedure may affect mothers’ recall. Caesarean delivery rates were 6% or lower in Malawi and Uganda during the whole study period and in intervention and comparison areas. In Bangladesh, caesarean delivery rates were 7.6% in intervention areas and 6.3% in comparison areas at baseline, but jumped to 15.1% in intervention areas at endline while only increasing to 8.1% in comparison areas. The doubling of caesarean delivery rates may reflect the doubling of facility delivery rates in intervention areas in Bangladesh. It was not possible to exclude caesarean deliveries from the Nepal dataset as the mode of birth was not recorded; the rate of caesarean delivery is less than 5% in the sub-region where the study was implemented, according to the 2011 Demographic and Health Survey (28).

### Variables

#### Changes in newborn care practices over time and cross-country comparisons

To examine changes in newborn care practices after roll-out of community-based programmes, we selected key breastfeeding, thermal protection, and cord care practices over which mothers are most likely to have some control, regardless of where the birth takes place, and could thus be influenced by counselling and messages. In addition, practices were selected based on comparability over time and across countries and the mother's ability to respond. Based on these criteria, the following behaviours were included: early initiation of breastfeeding (≤1 hour after birth); delayed bathing (≥6 hours after birth); skin-to-skin contact after birth (data on skin-to-skin only collected at endline); and nothing being applied to the cord. Cord-cutting practices were collected, but could not be included in analysis due to large numbers of women who did not know what was used to cut the cord. WHO recommendations on postnatal care (29) advise delayed bathing for 24 hours after birth, but if not possible due to cultural reasons, delaying at least 6 hours. We used 6 hours for analysis as per these recommendations.

In Nepal only women who gave birth at home were asked about substances applied to the cord. Since few women interviewed in Nepal delivered at home, the percentage reporting nothing being applied to the cord may not be representative and, therefore, was not included in analysis for this country. In Bangladesh, all women were asked about breastfeeding initiation, but only those who gave birth outside a facility were asked about the other practices. However, the majority of women gave birth outside a facility (74%), so analysis for Bangladesh included only non-facility births. In Uganda, the question on timing of first bath was added to the questionnaire during baseline survey implementation, leading to high numbers of missing values, so baseline data are not reported. Also, interviewers in Uganda recorded a categorical response on the timing of breastfeeding initiation at baseline, while the time in hours or days was recorded at endline. This change made the endline questionnaire comparable to other countries, but may have affected internal comparability over time. For the purpose of evaluating changes over time, missing values were excluded from the denominator to avoid skewing the absolute percentage change over time.

#### Pregnancy home visits and newborn care practices

A composite indicator of newborn care practices was created to determine if women who received home visits from CHWs during pregnancy were more likely to report use of healthy newborn care practices at endline. The composite indicator and main outcome variable for this analysis includes early initiation of breastfeeding bathing delayed at least 6 hours, and skin-to-skin contact. Applying nothing to the cord was not included in the composite indicator because we wanted to include only those indicators for which we had endline data from all countries. As explained above, we could not report endline data from Nepal on cord care because only women who gave birth at home were asked the appropriate questions and the numbers were small. For the purpose of creating a composite, missing values for any of the practices were treated as if the woman reported that the practice was not used or responded ‘I don't know’.

The main independent variable was home visits during pregnancy, which was defined categorically (0, 1–2, and 3+ visits) to learn if receiving more visits had a greater association with use of key newborn care practices. Analyses controlled for receipt of antenatal care (0 versus at least 1 visit), place of birth (facility versus non-facility), maternal age (<20, 20–29, 30 + ), and maternal education (any versus none). For Bangladesh, the analysis excluded women who gave birth at a facility since data were not collected from these women due to skip patterns in the questionnaire, as previously mentioned.

### Statistical analyses

The Pearson chi-squared test, corrected for the survey design with second-order correction of Rao and Scott (30), was used to assess changes in practices over time. Generalised linear models (GLM) were used to assess the relationship between the main independent variable, home visit from a CHW during pregnancy, and the composite outcome indicator. Relative risks (RR) and 95% confidence intervals (CI) were obtained. Clustering was controlled for in all analyses; appropriate standard error estimates were produced for the GLM using the Taylor linearisation method (31). Stata version 11 was used (32).

### Ethical considerations

All programmes were implementing national policy through routine systems. Ethical clearance was obtained from the National Health Sciences Research Committee in Malawi, the Bangladesh Medical and Research Council, Makerere University School of Public Health in Uganda, and the Uganda National Council of Science and Technology. As per approved applications to the institutional review boards, women gave verbal consent to participate in the survey in Bangladesh and Malawi and written consent in Uganda. In Nepal, data collection was completed as part of routine programme activities. Relevant district authorities granted permission, and all respondents provided verbal consent upon being informed of the purpose of data collection.

## Results

### Changes in newborn care practices

Table 4 shows newborn care practices at baseline and endline in each country, and the absolute percentage change over time. Missing values were less than 3% for each practice, unless otherwise noted in the table footnotes.

**Table 4:** Newborn care practices at baseline and endline and absolute percent change over time

		Malawi intervention			Nepal intervention			Bangladesh^a^ intervention			Bangladesh^a^ comparison			Uganda intervention			Uganda comparison		
					
		Pre (%)	Post (%)	% change	Pre (%)	Post (%)	% change	Pre (%)	Post (%)	% change	Pre (%)	Post (%)	% change	Pre (%)	Post (%)	% change	Pre (%)	Post (%)	% change
Early initiation of breastfeeding	Yes	74	95	21*	64	90	26*	68	75	8	69	80	11*	54	86	32*	47	85	38*
	No/DK	26	5		36	10		32	25		31	20		46	14		53	15	
Delayed bathing ≥6 hours	Yes	64	91	27*	78	95	17*	67	87	20*	63	73	10*	NC	90		NC	84	
	No/DK	36	9		22	5		33	13		37	27			10			16	
Skin-to-skin care	Yes	NC	68		NC	76		NC	80		NC	73		NC	82		NC	77	
	No/DK		32			24			20			27			18			23	
Nothing applied to cord	Yes	72	71	−1	NA	NA		48	79	31*	57	63	6	44	64	20*	58	52	−6
	No/DK	28	29					52	21		43	37		56	36		42	48	

a Non-facility births only.

* Statistically significant change from baseline to endline at *p*<0.05.

NC=not collected; DK=don't know; NA=not available (questions on substances applied to the cord were only asked for non-facility births in Nepal).

Missing values <3% except: 16.7% missing early initiation of breastfeeding at baseline in Malawi, 9.2% missing delayed bathing at baseline in Malawi, 10.8% missing delayed bathing at endline in Malawi, 6.2% missing delayed bathing at baseline in Nepal. Missing values excluded from denominator to calculate absolute percentage change. Shaded portion distinguishes data from comparison areas for the 2 countries that collected data from non-intervention areas.

In intervention areas there were statistically significant improvements in all practices, except applying nothing to the cord in Malawi and early initiation of breastfeeding in Bangladesh. Statistically significant changes in intervention areas ranged from 17% (delayed bathing in Nepal) up to 32% (early initiation of breastfeeding in Uganda).

In Bangladesh and Uganda, where data were collected from comparison areas, most practices increased in both the intervention and comparison areas. However, in Bangladesh endline proportions were higher in the intervention than the comparison areas for delayed bathing, skin-to-skin contact, and nothing applied to the cord (*p*<0.05). The increase in early breastfeeding was borderline significant in intervention areas (*p*=0.06), while it was statistically significant in comparison areas (*p*=0.01), but the endline proportions were not statistically significantly different between intervention and comparison areas. In Uganda, endline proportions of delayed bathing and nothing applied to the cord were higher in intervention areas than comparison areas (*p*<0.05). The increase in early breastfeeding was greater in comparison areas, but there was no difference between areas at endline.

### Number of practices applied

The number of women who reported use of 0, 1, 2, or all 3 of the newborn care practices included in the composite indicator at endline is shown in Table 5, as well as the average number of practices used. In all countries, half or more of interviewed women reported that all three practices were done. Very few women reported none of these practices being applied.

**Table 5:** Number of practices used at endline, among the three practices included in composite indicator

	Malawi (N=844)		Nepal (N=615)		Bangladesh^a^ (N=293)		Uganda (N=869)	
			
No. of practices	n	%	n	%	n	%	n	%
0	6	1	8	1	3	1	9	1
1	49	6	33	5	33	11	51	6
2	367	44	155	25	93	32	252	29
3	422	50	419	68	164	56	557	64
Mean	2.43		2.60		2.43		2.56	

a Non-facility births only.

### Association between pregnancy home visits and newborn care practices

Table 6 shows the distribution of independent variables for women interviewed in intervention areas at endline and asked about practices included in the composite outcome indicator (early initiation of breastfeeding, delayed bathing, and skin-to-skin contact). Nearly all women in Nepal and Bangladesh reported at least one home visit during pregnancy from a CHW and the majority reported 3 or more visits. In Malawi, 62.1% of women reported no home visit while only 7.6% reported 3 or more. In Uganda, about a third of women fell into each category – no visit, 1–2 visits, and 3 or more visits. Nearly all women in Malawi and Uganda received antenatal care (95.3 and 95.7%), but fewer did in Nepal (65.5%) and Bangladesh (42.3%). Facility birth rates were highest in Malawi (92.1%), followed by Nepal (81.5%), then Uganda (77.3%). These rates were higher than 2010/2011 national averages (28, 33, 34). Facility delivery rates were much lower in Bangladesh (26.4%); only data from non-facility births are included in the analysis.

**Table 6:** Distribution of independent variables for women asked about the three practices included in composite indicator at endline

	Malawi (N=844)		Nepal (N=615)		Bangladesh^a^ (N=293)		Uganda (N=869)	
			
	n	%	n	%	n	%	n	%
Pregnancy home visit								
0	524	62	18	3	26	9	288	33
1 or 2	248	29	89	14	78	27	300	35
3+	64	8	508	83	189	65	279	32
Missing	8	1	0	0	0	0	2	<1
Antenatal care								
None	32	4	212	34	169	58	36	4
At least one visit	804	95	403	66	124	42	832	96
Missing	8	1	0	0	0	0	1	<1
Place of delivery								
Home	54	6	114	19			197	23
Facility	777	92	501	81			672	77
Missing	15	2	0	0			0	0
Maternal age (years)								
<20	121	14	96	16	41	14	109	13
20–29	473	56	453	74	186	63	485	56
30+	230	27	66	11	66	23	275	32
Missing	20	2	0	0	0	0	0	0
Maternal education								
None	103	12	258	42	54	18	97	11
Any	736	87	357	58	239	82	772	89
Missing	5	1	0	0	0	0	0	0

a Non-facility births only.

Table 7 shows the unadjusted and adjusted RR that all three practices were used. In Malawi, Nepal, and Bangladesh women visited once or twice during pregnancy were more likely to report that all three practices were used, but the difference was not statistically significant. In Malawi, women were 34% more likely to report that their newborns were breastfed early, bathed ≥6 hours after birth, and given skin-to-skin contact if visited at least three times during pregnancy, controlling for other independent variables (*p*<0.05). In Bangladesh, where only non-facility births were included in the analysis, women were 88% more likely to report that their newborns received all three recommended practices if visited at least three times during pregnancy (*p*<0.05). The same trend was seen in Nepal, but very few women received no visits during pregnancy and there was no statistically difference between women who received at least three visits and those that received none. In Uganda women who received pregnancy home visits were not more likely to report that their newborns received all three practices compared to women that did not receive a home visit. However, when we did separate analysis for each behaviour, delayed bathing was statistically significantly associated with three or more pregnancy home visits in Uganda.

**Table 7:** Generalised linear models for four countries with a composite indicator of early initiation of breastfeeding, delayed bathing, and skin-to-skin contact at endline as the dependent variable

	Malawi				Bangladesh^a^				Nepal				Uganda			
			
	n	Crude RR	Adjusted (95% CI)	*p*	n	Crude RR	Adjusted (95% CI)	*p*	n	Crude RR	Adjusted (95% CI)	*p*	n	Crude RR	Adjusted (95% CI)	*p*
Pregnancy home visits																
None (Ref)	489	1.00	1.00		26	1.00	1.00		18	1.00	1.00		287	1.00	1.00	
1 or 2	235	1.07	1.07 (0.91–1.27)	0.410	78	1.30	1.36 (0.77–2.39)	0.278	89	1.52	1.37 (0.89–2.12)	0.143	299	0.99	1.01 (0.90–1.14)	0.819
3+	62	1.36	1.34 (1.11–1.62)	0.003	189	1.83	1.88 (1.06–3.35)	0.032	508	1.55	1.45 (0.95–2.20)	0.082	277	1.00	1.01 (0.89–1.15)	0.840
ANC^b^ visit																
None (Ref)	29	1.00	1.00		169				212	1.00	1.00		36	1.00	1.00	
At least one	757	1.06	0.97 (0.65–1.45)	0.893	124	1.12	1.14 (0.90–1.44)	0.265	403	1.05	1.00 (0.88–1.13)	0.977	827	1.38	1.29 (0.90–1.85)	0.153
Place of delivery																
Non-facility (Ref)	46	1.00	1.00		0	1.00	1.00		114	1.00	1.00		196	1.00	1.00	
Facility	740	2.20	2.25 (1.28–3.93)	0.005	293	–	–	–	501	2.10	2.11 (1.56–2.84)	0.000	667	1.48	1.47 (1.30–1.66)	0.000
Maternal age																
<20 (Ref)	114	1.00	1.00		41	1.00	1.00		96	1.00	1.00		109	1.00	1.00	
20–29	453	1.23	1.22 (0.99–1.51)	0.056	186	1.21	1.25 (0.82–1.89)	0.287	453	1.00	1.00 (0.87–1.16)	0.962	480	1.15	1.16 (1.01–1.33)	0.041
30+	219	1.29	1.30 (1.02–1.66)	0.034	66	1.06	1.03 (0.68–1.56)	0.886	66	0.90	0.94 (0.77–1.14)	0.516	274	1.04	1.11 (0.93–1.32)	0.252
Maternal education																
None (Ref)	90	1.00	1.00		54	1.00	1.00		258	1.00	1.00		95	1.00	1.00	
Any	696	1.11	1.09 (0.84–1.41)	0.534	239	1.10	1.00 (0.77–1.31)	0.996	357	1.03	0.94 (0.83–1.06)	0.312	768	0.99	0.98 (0.85–1.12)	0.711
Total	786				293				615				863			

a Non-facility births only.

b Antenatal care (at LEAST one visit with a skilled provider).

<10% missing one or more independent variable and excluded from model (approximately 7% missing values in Malawi, 1% in Uganda and 0% in Nepal and Bangladesh).

Mothers were more likely to report use of all three practices if the birth happened at a facility. In Malawi older mothers were also more likely to have reported use of all three practices. In Uganda women aged 20–29 years were more likely to have reported these practices, but not mothers over 30 years.

## Discussion

This multi-country study provides important information on the extent to which community-based packages can be expected to influence care for newborns. The packages examined in this analysis all included home visits by CHWs to promote and support evidence-based newborn care practices, with varying emphasis on other inputs such as facility strengthening.

There were sharp increases in most selected newborn care practices over a short period of time, in all countries and in both intervention and comparison areas. Changes cannot be completely attributed to the community-based interventions, and there are a number of other factors that may have contributed, including the rapid rise in facility delivery rates. Whether or not the birth took place at a facility appears to be the most important contributor to use of the selected practices. Yet use of all three practices (early initiation of breastfeeding, delayed bathing, and skin-to-skin contact) was still moderate in Malawi, Nepal, and Uganda (49, 68, and 64%, respectively) where facility birth rates were high. Thus increasing the promotion and use of these practices is still needed at health facilities.

As noted, changes in behaviours over time need to be interpreted with caution, given the dynamic environment and changes in measurement from baseline to endline. However, it is interesting to note the lack of change in dry cord care in Malawi, where mothers who deliver at a facility may have little control over this practice. Further, the CHW training manual included messages about appropriate cord care under items to discuss during postnatal home visits, when it may be too late to influence practice. Timing of family counselling about newborn care practices is important to consider when designing delivery approaches. Since many of these practices need to be applied soon after birth, messages need to be given to women and families during pregnancy and reinforced after birth. Early initiation of breastfeeding did not significantly increase in intervention areas in Bangladesh, suggesting that the programme needs to examine how to improve promotion and support of breastfeeding. However, it is important to note that the rates of early breastfeeding initiation were high at baseline and there were existing nutrition interventions under the National Nutrition Program.

Home visits during pregnancy were associated with a greater likelihood of using selected practices in two countries – Malawi and Bangladesh – and a similar although non-significant trend was seen in Nepal, possibly due to inability to detect a statistically significant difference given the very small number of women reporting no home visits during pregnancy. In Bangladesh, most women received pregnancy visits, and there was a strong association between home visits and use of practices. High coverage of visits could have helped change norms, facilitating widespread uptake of these practices. Also, only home births were included in the analysis of Bangladesh data, and counselling may be more likely to change practices when a woman delivers at home.

In Malawi, home visit coverage was low and the strength of the relationship between home visits and practices is weaker. In both these countries, there may be a dose-response – while one or two home visits was associated with an increased likelihood that newborn care practices were applied, statistically significant associations were only seen when more than three antenatal home visits were received. Thus, it appears that a sufficient number of visits are needed to reinforce and support behaviours. However, it may not be feasible in all contexts for CHWs to visit homes three or more times during pregnancy, and some of the programmes were not even designed to visit women that many times. There is a need for closer examination into how programmes can deliver messages and promote behaviour change, in addition to home visits. These programmes incorporated community mobilisation and mass media messages to different extents; there is a need to understand the influence of such components and how they interact with home visits, although data were not available in these pilot areas for such analysis.

Uganda was the only country where home visits did not appear to have an association with the composite indicator. It is possible that the effect of home visits on these practices was diluted by other intervention elements, including facility quality improvement efforts and community mobilisation. The rapid changes in comparison areas suggest that other programmes and secular trends were influencing these practices, and possibly a spill-over effect from intervention areas. Practices can change rapidly and programmes come and go, so health managers need to monitor what is happening on a regular basis to ensure additional efforts to promote practices as necessary and appropriate.

In addition, it is difficult to tease out the importance of CHW characteristics on the quality of counselling and the likelihood that women and families will heed counselling advice, although they are probably important. In Malawi, where coverage of home visits was low but there was an association between home visits and practices, CHWs are trained, government-employed workers who also work at facilities, so community members recognise them as health professionals and an extension of the formal health system. That may have increased the influence of their home-based counselling on practices. In Uganda, CHWs are community members and part-time volunteers with substantially less training that may or may not be seen as part of the health system. Therefore, their home-based counselling may not be as effective in changing practices.

Although existing government structures, systems, and authorised employees or volunteers were used, implementation required additional inputs. Ministries of health in these countries are in the process of rolling out community-based newborn care programmes that were informed by the pilot programmes described here (22–25), and the amount of external implementation support varies within and across countries. Even where there is support, it has been difficult to reach every woman and newborn with home visits (26), and there are implementation challenges including the difficulty of identifying all pregnant women, ensuring and maintaining skills for large numbers of workers, and tracking programme-relevant data collected at community level without overburdening community workers (35).

Programmes such as those described here are often embedded in a long programmatic history. For example, in Nepal there was a safe delivery incentive programme to increase institutional delivery with Female Community Health Volunteers conducting pregnancy home visits that included counselling on the newborn care practices examined in this paper (36, 37). Previous efforts can have an important effect on the current programme of interest. More importantly, we should not assume that positive programme effects mean high performance continues after outside partner attention and inputs are removed. Although programme performance is often measured at the height of implementation efforts, experience demonstrates that performance often declines and most or all of the observed benefit may be lost.

Analytical limitations include differences in household survey questionnaires; variations that may affect comparability have been noted. Use of a composite indicator masks the association between home visits and each individual behaviour, some of which were not statistically significant. Questionnaires were administered to women with a live birth in the previous year and may have introduced recall or reporting bias. Research shows that women have been able to accurately recall some behaviours (particularly skin-to-skin care) but may have difficulty recalling others (38). There is the possibility that women falsely report behaviours that are believed to be socially desirable; this bias may have been stronger among women exposed to the intervention. Finally, there are many factors that contribute to newborn care practices which may not have been captured by the confounding factors in the model. Newborn care practices could have been influenced by the community engagement activities done under these and other programmes, mass media messages, other actors, and shifting social and cultural norms, which we were not able to measure and incorporate into these analyses.

Data collection efforts were not powered to measure impact on mortality. We also could not estimate the impact using modelling programmes such as the Lives Saved Tool because the effectiveness and coverage of several of the interventions included in this analysis are not currently included in the tool. Efforts to expand the Lives Saved Tool to include more of these healthy behaviours will help countries estimate mortality changes based on changes in coverage of evidence-based interventions.

## Conclusion

This analysis shows that newborn care practices can rapidly change along with the context in which they are used. Home visits by CHWs during pregnancy can play a role in improving practices in different settings. However, multiple interactions are probably needed to change behaviour, so programmes need to investigate the most appropriate and efficient ways to reach families and promote newborn care practices. The magnitude of the association between home visits and use of key practices was greatest in Bangladesh, where only non-facility births were included in the model, indicating that the impact of home visit programmes on newborn care practices may be most profound in areas with high rates of home delivery. At the same time, facility delivery appears to be the most important predictor for ensuring newborn care practices, so programmes need to take advantage of increasing facility delivery rates to ensure that all babies benefit from these practices.

## References

[1] Lawn JE, Cousens S, Zupan J (2005). 4 million neonatal deaths: when? where? why?. Lancet.

[2] WHO, UNICEF (2012). Countdown to 2015: building a future for women and children.

[3] UNICEF, WHO, World Bank, United Nations Population Division (2012). Levels and trends of child mortality: report 2012.

[4] The Partnership for Maternal, Newborn & Child Health (2011). A global review of the key interventions related to reproductive, maternal, newborn and child health (RMNCH).

[5] Mullany LC, Katz J, Li YM, Khatry SK, LeClerq SC, Darmstadt GL (2008). Breast-feeding patterns, time to initiation, and mortality risk among newborns in southern Nepal. J Nutr.

[6] Leon-Cava N, Lutter C, Ross J, Martin L (2002). Quantifying the benefits of breastfeeding: a summary of the evidence.

[7] Blencowe H, Cousens S, Mullany LC, Lee AC, Kerber K, Wall S (2011). Clean birth and postnatal care practices to reduce neonatal deaths from sepsis and tetanus: a systematic review and Delphi estimation of mortality effect. BMC Public Health.

[8] Mullany LC, Darmstadt GL, Khatry SK, Katz J, LeClerq SC, Shrestha S (2006). Topical applications of chlorhexidine to the umbilical cord for prevention of omphalitis and neonatal mortality in southern Nepal: a community-based, cluster-randomized trial. Lancet.

[9] Arifeen SE, Mullany LC, Shah R, Mannan I, Rahman SM, Talukder MR (2012). The effect of cord cleansing with chlorhexidine on neonatal mortality in rural Bangladesh: a community-based, cluster-randomised trial. Lancet.

[10] Soofi S, Cousens S, Imdad A, Bhutto N, Bhutta ZA (2012). Topical applications of chlorhexidine to neonatal umbilical cords for prevention of omphalitis and neonatal mortality in a rural district of Pakistan: a community-based, cluster-randomized trial. Lancet.

[11] Mullany LC (2010). Neonatal hypothermia in low-resource settings. Semin Perinatol.

[12] Kumar V, Shearer JC, Kumar A (2009). Neonatal hypothermia in low resource settings: a review. J Perinatol.

[13] El-Radhi AS, Jawad M, Mansor N, Jamil I, Ibrahim M (1983). Sepsis and hypothermia in the newborn infant: value of gastric aspirate examination. J Pediatr.

[14] El-Radhi AS, Jawad MH, Mansor N, Ibrahim M, Jamil II (1983). Infection in neonatal hypothermia. Arch Dis Child.

[15] Dincsoy MY, Siddiq F, Kim YM (1990). Intracranial hemorrhage in hypothermic low-birth-weight neonates. Childs Nerv Syst.

[16] World Health Organization (1997). Thermal protection of the newborn: a practical guide. Maternal health and safe motherhood programs.

[17] Darmstadt GL, Choi Y, Arifeen SE, Bari S, Rahman SM, Mannan I (2010). Evaluation of a cluster-randomized controlled trial of a package of community-based maternal and newborn interventions in Mirzapur, Bangladesh. PLoS ONE.

[18] Baqui AH, El-Arifeen S, Darmstadt GL, Ahmed S, Williams EK, Seraji HR (2008). Effect of community-based newborn-care intervention package implemented through two service delivery strategies in Sylhet district, Bangladesh: a cluster-randomised controlled trial. Lancet.

[19] Kumar V, Mohanty S, Kumar A, Misra RP, Santosham M, Awasthi S (2008). Effect of community-based behaviour change management on neonatal mortality in Shivgarh, Uttar Pradesh, India: a cluster-randomised controlled trial. Lancet.

[20] Bhutta ZA, Soofi S, Cousens S, Mohammad S, Memon ZA, Ali I (2011). Improvement of perinatal and newborn care in rural Pakistan through community-based strategies: a cluster-randomised effectiveness trial. Lancet.

[21] WHO, UNICEF (2009). WHO-UNICEF Joint Statement on home visits for the newborn child: a strategy to improve survival.

[22] Rubayet S, Shahidullah M, Hossain A, Corbett E, Moran AC, Mannan I (2012). Newborn survival in Bangladesh: a decade of change and future implications. Health Policy Plan.

[23] Pradhan YV, Upreti SR, Pratap KCN, Ashish KC, Khadka N, Syed U (2012). Newborn survival in Nepal: a decade of change and future implications. Health Policy Plan.

[24] Zimba E, Kinney MV, Kachale F, Waltensperger KZ, Blencowe H, Colbourn T (2012). Newborn survival in Malawi: a decade of change and future implications. Health Policy Plan.

[25] Mbonye AK, Sentongo M, Mukasa GK, Byaruhanga R, Sentumbwe-Mugisa O, Waiswa P (2012). Newborn survival in Uganda: a decade of change and future implications. Health Policy Plan.

[26] Sitrin D, Guenther T, Murray J, Pilgrim N, Rubayet R, Ligowe R (2013). Reaching mothers and babies with early postnatal home visits: the implementation realities of achieving high coverage in large-scale programs. PLoS ONE.

[27] Waiswa P, Peterson SS, Namazzi G, Ekirapa EK, Naikoba S, Byaruhanga R (2012). The Uganda Newborn Study (UNEST): an effectiveness study on improving newborn health and survival in rural Uganda through a community-based intervention linked to health facilities – study protocol for a cluster randomized controlled trial. Trials.

[28] Ministry of Health and Population (MoHP) [Nepal], New ERA Ltd., ICF International Inc (2012). Nepal demographic and health survey 2011.

[29] World Health Organization (2014). WHO recommendations on postnatal care of the mother and newborn.

[30] Rao JNK, Scott AJ (1984). 2014. On chi-squared tests for multiway contingency tables with cell proportions estimated from survey data. Ann Statist.

[31] Wolter KM (2003). Introduction to variance estimation.

[32] StataCorp (2010). Stata Statistical Software: Release 11.

[33] NSO Malawi, ICF Macro (2011). Malawi demographic and health survey 2010.

[34] Uganda Bureau of Statistics (UBOS), ICF International Inc (2012). Uganda demographic and health survey 2011.

[35] Nalwadda KC, Naamala SH, Ssekitooleko J, Kerber K, Kallander K, Waiswa P (2012). Introduction of newborn care within integrated community case management in Uganda. Am J Trop Med Hyg.

[36] McPherson RA, Khadka N, Moore JM, Sharma M (2006). Are birth-preparedness programmes effective? Results from a field trial in Siraha district, Nepal. J Health Popul Nutr.

[37] Powell-Jackson T, Morrison J, Tiwari S, Neupane BD, Costello AM (2009). The experiences of districts in implementing a national incentive programme to promote safe delivery in Nepal. BMC Health Serv Res.

[38] Stanton CK, Rawlins B, Drake M, dos Anjos M, Cantor D, Chongo L (2013). Measuring coverage in MNCH: testing the validity of women's self-report of key maternal and newborn health interventions during the peripartum period in Mozambique. PLoS One.
